# Taxonomy and phylogeny of the epiphytic sooty molds in family *Metacapnodiaceae* (class *Eurotiomycetes*, subclass *Chaetothyriomycetidae*)

**DOI:** 10.3897/mycokeys.129.178067

**Published:** 2026-03-04

**Authors:** Faezeh Aliabadi, Ludovic Le Renard, Mary L. Berbee

**Affiliations:** 1 Department of Botany, University of British Columbia, 6270 University Blvd, Vancouver, BC V6T 1Z4, Canada Department of Botany, University of British Columbia Vancouver Canada

**Keywords:** *
Capnodiales
*, fossil, ITS barcodes, *
Metacapnodium
*, phylogeny, *
Pleostigmataceae
*, *
Sorocybe
*, *
Verrucariales
*

## Abstract

*Metacapnodiaceae* is one of several sooty mold families in *Ascomycota*. Its species grow as dense, black, spongy mats on the surfaces of plant leaves and twigs, typically in association with insect honeydew or plant leachates. Studying *Metacapnodiaceae* has been challenging because multiple species intermingle in the same small patches of mycelium, and they are difficult to culture or to distinguish by their morphology. Prior to this research, DNA sequences were available for only two species in the family. Here, with the goal of better characterizing species diversity, we determined complete or partial DNA sequence barcodes for ribosomal internal transcribed spacer (ITS) regions for 16 collections of *Metacapnodium* using a *Metacapnodium*-specific primer, followed by phylogenetic and morphological analyses. Tapering, moniliform hyphae, cells wider than long, and a distinctive phialidic asexual state were good predictors of membership in a well-supported *Metacapnodium* clade. Sequences from the 16 collections represent 9 named species of *Metacapnodium*. Barcoded species include: *M. stanhughesii***sp. nov**., *M. vancouverensis*, **sp. nov**. and *M. australis***comb. nov**. Based on morphological characters, we propose *M. atro-olivaceus***comb. nov**., *M. novae-zelandiae***comb. nov**., and *M. pacificus***comb. nov**. We provide a key to identification of 15 species studied. To investigate the deeper phylogenetic relationships of *Metacapnodiaceae*, we sequenced partial nuclear ribosomal large subunit (LSU) gene regions from five specimens and elongation factor 1-alpha (*ef1-α*) gene regions from two specimens. In our analysis of concatenated sequences from ribosomal DNA, *ef1-α*, and from *rpb2*, the gene encoding the 2^nd^ largest subunit of RNA polymerase II protein, *Metacapnodium* appeared within the subclass *Chaetothyriomycetidae*, class *Eurotiomycetes* with strong support, and as the sister group to *Pleostigmataceae* but without strong statistical support. Our study adds *Metacapnodiaceae* to the clades of enigmatic, slow growing fungi of harsh environments with lichenized, lichenicolous, resinicolous and rock-inhabiting niches. Resolving family relationships is relevant to age estimates of *Ascomycota*, as fossilized *Metacapnodium* specimens in amber potentially contribute to calibration of divergence times.

## Introduction

**Figure 1.**
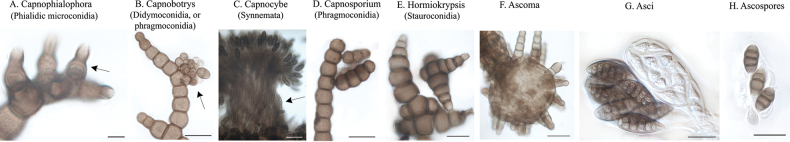
Sexual and asexual forms of *Metacapnodiaceae*. **A**. Phialides with a globose to subglobose venter (arrow) and a collarette at the neck, *Metacapnodium ericophilum*DAOM 234182; **B**. Globose to subglobose conidiogenous cells produce successive blastic conidia, *M. stanhughesii*UBC F35817; **C**. Synnema (arrow) of tightly compacted conidiophores, bearing conidiogenous cells and conidia at their tips, *M. spongiosum* (OSC61673); **D**. Capnosporium conidia with 2–4 septa, *M. stanhughesii*UBC F35817; **E**. Hormiokrypsis conidia with a straight, multicellular main axis and two multicellular, tapered arms, hormiokrypsis form of *Ophiocapnocoma phloiophilia* OSC61673; **F**. Ascoma with appendages, *M. moniliforme*DAOM 226251; **G**. Asci, *M. adamantinum* OSC 169460; **H**. Ascospores, *M. moniliforme*DAOM 226251. Scale bars: 5 µm (**A**); 20 μm (**B, D–H**); 50 µm (**C**).

*Metacapnodiaceae* is one of several families of sooty mold fungi that inhabit plant leaves, twigs, and trunks as black spongy subicula composed of loosely tangled hyphae (Hughes 1972, 1976). Its species occur mainly on the surfaces of plants that are covered by insect honeydew or plant leachates (Auclair 1963), although they are sometimes found on plants that have neither of these substances present. Sooty molds thrive in moist coastal temperate forests of the southern hemisphere, but have also been observed in tropical, subtropical, and north temperate environments (Chomnunti et al. 2014). Fig. 1 illustrates reproductive features of representative species in *Metacapnodiaceae*. The family is characterized by dark brown hyphae, tapering at their tips, and hyphal cells that are broader than long (Fig. 1B) (Hughes 1972, 1976). Constrictions at septa give hyphae a bead-like or moniliform appearance (Fig. 1B).

The same *Metacapnodium* species may have two or more asexual forms. Historically, the names of the conidial forms were, as explained below, applied to different asexual genera. Currently, the names are used to convey distinctive combinations of morphological character states that are useful in species recognition (Hughes 1976). Names of forms convey the shapes, sizes, and arrangements of conidia and the cells that produce them.

The capnophialophora asexual form consists of phialides with a brownish, globose, subglobose, or hemispherical venter and a prominent collarette (Fig. 1A) (Hughes 1966). Conidia from phialides are aseptate, hyaline, and inconspicuous. The phialides often emerge from the sides or tips of somatic hyphal cells, but they may also bud from perithecial wall cells, or from ascospores or other conidia. Most *Metacapnodium* species produce a capnophialophora state.

The capnobotrys asexual form consists of conidia that appear in clusters, budding from conidiogenous cells, which arise from adjacent somatic cells or occasionally, from one another (Hughes 1981). Capnobotrys conidia are two-celled in most but not all species. The conidia are commonly inequilateral, narrowing towards their tips, with an apical cell wall thinning that may serve as a germ pore. Proliferation of successive conidia is sympodial, each conidium budding from a new growing point on its conidiogenous cell. Upon secession, the conidium may leave a denticulate scar on its conidiogenous cell. Several *Metacapnodium* species produce a capnobotrys state.

The capnocybe (Hughes 1966) asexual form is synnematal (Fig. 1C). Synnema are usually 1.0–8.0 mm tall with stalks consisting of closely appressed, ascending hyphae (Hughes 1966; Hawksworth and Boluda 2020). Heads consist of hyphae with penicillate branching, which, at their tips, produce a sympodial succession of transversely septate, ovoid conidia.

The capnosporium-type conidia (Hughes 1976) (Fig. 1D) have two or more transverse septa. They bud from a narrow pore in a conidiogenous cell. Aside from the pore, the conidiogenous cell is an apparently undifferentiated apical or lateral somatic hyphal cell, or an older capnosporium conidium. The hormiokrypsis-type conidia (Fig. 1E) similarly bud from a narrow pore and are multiseptate, but develop into distinctive, cruciate structures when one or two lateral arms bud from their main axes (Hughes 1967).

Fragments of somatic hyphae may also play a role in asexual reproduction in *Metacapnodium*. However, the fragments are not usually given names, and unlike conidia, they are not released at a pre-formed point of secession visible as pore or as a noticeable narrowing at a septum.

### Systematic history

Past controversy about relationships of *Metacapnodiaceae* was partially resolved by increasingly sophisticated observations on the development of the ascomata, and reinforced more recently by molecular systematics. Hughes (1972) first erected *Metacapnodiaceae* S. Hughes & Corlett in order *Pleosporales* on the basis of what Corlett (1970) interpreted as a *Pleospora*-type centrum, characterized by pseudoparaphyses that grew from the ceiling of the reproductive cavity downward among the asci. This character, together with tapering hyphal tips and superficial conidiogenous cells separated *Metacapnodiaceae* from other sooty molds in other families, notably *Capnodiaceae*, which have a *Dothidea*-type centrum, cylindrical hyphae, and pycnidial asexual forms (Corlett et al. 1973). However, Corlett (1970) noted that the pseudoparaphyses of the *Metacapnodium juniperi* centrum were slightly atypical for *Pleosporales* because they did not reach the bottom of the cavity and instead persisted only at the top of the locule. Barr (1979) considered that these short pseudoparaphyes, or paraphysoids indicated deep phylogenetic separation from *Pleosporales*, and erected *Chaetothyriales*, a new order, for the perithecioid fungi that have them. Winka et al. (1998) used ribosomal DNA evidence to show that members of the *Chaetothyriales* were indeed distantly related to *Pleosporales*. Hawksworth and Boluda (2020) determined the first ITS sequences for *Metacapnodium ericophilum*, and their analysis placed *Metacapnodium* in *Eurotiomycetes*, somewhere near *Phaeomoniellales* and *Chaetothyriales*. Based on nrSSU phylogenetic analysis, Sugiyama et al. (2020) inferred that *Metacapnodium neesii* was the sister to all of their examined taxa in *Chaetothyriales*, with high bootstrap support. Having established *M. neesii* in pure culture facilitated sequencing not only of its 479 bp ITS region but also of 900 bp from the 5’ end of the ribosomal large subunit gene (nuLSU), 1074 bp of the 5’ end of the ribosomal small subunit gene (nuSSU), 777 bp of the gene encoding the polymerase II second largest subunit (*rpb2*), and 892 bp of the gene encoding the elongation factor 1–alpha (*ef1-α*) (Sugiyama et al. 2020). These Sugiyama et al. (2020) sequences contributed to our ability to select or design *Metacapnodium* specific primers in this study.

After decades of confusion, integration of names of sexual and asexual forms of *Metacapnodium* has largely been resolved. Rossman et al. (2016) recommended conserving *Metacapnodium* Speg. 1918 against *Antennularia* Rchb. 1828. Following this recommendation, *Metacapnodium* has been approved for protected status under Article F.2.1 of the International Commission on the Taxonomy of Fungi and the Nomenclature Committee for Fungi against *Antennularia*, and against other names for the sexual or asexual forms that were not formally considered (May 2024). This leaves open a biological question of whether all species once classified only as asexual forms including *Capnobotrys*, *Capnocybe*, *Capnophialophora*, and *Capnosporium* should be transferred to *Metacapnodium*.

Investigating systematics of *Metacapnodiaceae* still poses significant challenges. Firstly, multiple species of sooty molds can inhabit the same subiculum (Hughes 1972; Hughes and Seifert 2012). Isolating sooty molds in *Metacapnodiaceae* into pure culture has been challenging. The lack of living cultures has made the resolution of phylogenetic relationships in *Capnodiales* and *Metacapnodiaceae* difficult (Crous et al. 2009). Because of these challenges, much of the richness in diversity of *Metacapnodiaceae* has yet to be discovered and the phylogenetic positions of the family and its species remain only partially resolved.

Clarifying the phylogeny of *Metacapnodiaceae* will improve interpretation of the fossil record of sooty mold fungi. Fossil specimens found in amber (Rikkinen et al. 2003; Schmidt et al. 2014) are attributed to the genus *Metacapnodium* and used as evidence for divergence times in molecular dating analyses (Beimforde et al. 2014; Samarakoon et al. 2019). The reliability of dating will be improved when fossils are interpreted with additional information about the phylogeny and morphology of extant taxa.

The aims of this project are to clarify the phylogenetic position of *Metacapnodiaceae* among ascomycetes using sequences of SSU, LSU, *ef1*–α and *rpb2* regions; to test the monophyly of *Metacapnodium* and the asexual forms predicted to be its members, and to provide DNA barcodes and a morphology-based key to aid in species identification.

## Methods

### Biological material – specimens examined, culturing method, and separation of individual species from multi-species tangles

We selected specimens of *Metacapnodiaceae*, collected more recently than 1990 where possible using the Mycology Collections Portal (Miller and Bates 2017). Table 1 contains accession information for the 20 specimens sequenced or appearing in figures. We collected three species of *Metacapnodium*, one from leaves of *Pieris japonica*, one from the stem of cultivated *Taxus* sp. from plants cultivated at the University of British Columbia campus, Vancouver, BC, and one species from Kauai’i, Hawaii, from leaves of *Metrosideros cf. polymorpha* (Table 1). Of the fresh collections, only the specimen from Hawaii grew in pure culture. Successful isolation of the specimen began by pipetting sterile potato dextrose broth over sterile Kimwipe tissues (Kimberly-Clark, Texas, USA) in glass Petri dishes (Keith Seifert 2015, Botanical Society of America Annual Meeting, personal communication). We quickly scraped conspicuous *Metacapnodium* thalli from fresh leaves of *Metrosideros cf. polymorpha* over the open Petri dish with a sterile needle, spreading fragments of hyphae. After 7–10 days, we could observe signs of hyphal growth under a dissecting microscope in uncontaminated areas of the wet tissue. We transferred promising colonies that occurred on larger fragments onto fresh BD DIFCO potato dextrose agar media (Becton Dickinson, Mississauga, Ontario) using a sterile insect pin. Several isolates showed signs of slow growth for a week before they stopped or were overgrown by contaminants. We obtained pure cultures from the isolates that grew faster. Of two isolates that were repeatedly subcultured, only one strain remained alive after six months. All plates and flasks of the second strain stopped growing within 6 months. The successful isolate took about 6 months to reach approximately 5 mm in diameter at room temperature.

**Table 1.** *Metacapnodiaceae* specimens illustrated or sequenced in this study.

Voucher specimens	Species*	Host	Locality	Collector	Nuclear rDNA ITS accession	Nuclear rDNA LSU/SSU accession**	*ef1-α* accession
*Metacapnodium adamantinum*	OSC 169460	*Arctostaphylos* sp.	USA, Oregon	B. McCune	OR532928 150 bp	PP140682 502 bp	–
*M. atro-olivaceus*	DAOM 106914 (split of holotype PDD 36102; consists of subiculum and microscope slides)	*Nothofagus fusca*	New Zealand, Westland	S.J. Hughes	–	–	–
*M. australis*	DAOM 106914 (mixed specimen, a split of holotype of *M. atro-olivaceus*)	*N. fusca*	New Zealand, Westland	S.J. Hughes	OR532937 181 bp	–	–
*M. dingleyae*	O F201720	*Taxus baccata*	Norway	D. Holton	OR532929 666 bp	–	–
*M. dingleyae*	O F281492	*T. baccata*	Norway	J.I. Johnson	OR532930 411 bp	–	–
*M. dingleyae*	O F293899	*T. baccata*	Norway	D. Holtan, P.G. Larsen	OR532931 631 bp	–	–
*M. ericophilum*	DAOM 234182	*Erica arborea*	Spain, Comunidad de Madrid	D.L. Hawksworth	OR532934 448 bp	PP140683 878 bp	–
*M. juniperi*	E 01043363	*Juniperus* sp.	Scotland	S. Furze	OR532938 590 bp	–	–
*M. moniliforme*	DAOM 226251	*Unknown leaves*	Australia, New South Wales	K. Seifert	OR532935 181 bp	–	–
*M. moniliforme*	DAOM 234317	*Cheirodendron trigynum*	USA, Hawaii	C. Inada	OR532936 162 bp	–	–
*M. cf. moniliforme*	UBC F33050; cultured as DAOMC 252865, CCCM F128	*Metrosideros cf. polymorpha*	USA	J. Dee	OR532924 1144 bp	PP140680 1161 bp PP836272 1306 bp	OR820948 253 bp
*M. aff. moniliforme*	E 01043362	*Rhododendron* sp.	Scotland	R. Yahr	OR532939 281 bp	PP140684 860 bp	–
*M. novae-zelandiae*	DAOM 97302a (split of holotype, PDD24925)	*N. fusca*	New Zealand	S.J. Hughes	–	–	–
*M. pacificus*	DAOM 117157c (Ex holotype: PDD 25871; microscope slide only)	*Coprosma tenuifolia*	New Zealand, Wellington	J.M. Dingley	–	–	–
*M. spongiosum*	OSC 135428	*Calocedrus decurrens*	USA, Oregon	R. Rippy	OR532927 385 bp	–	–
*M. stanhughesii*	UBC F35817 **holotype**	*Taxus* sp.	Canada	F. Aliabadi, L. Le Renard	OR532926 560 bp	PP140681 1129 bp	OR820949 253 bp
*M. vancouverensis*	UBC F35816 **holotype**	*Pieris japonica*	Canada	F. Aliabadi	OR532925 555 bp	–	–
*M.* sp.	DAOM 239041	*Betula papyrifera*	Norway	J.L. Crane	OR532933 181 bp	–	–
*M.* sp.	O F201613	*T. baccata*	Norway	D. Holton	OR532932 410 bp	–	–
*Ophiocapnocoma phloiophilia*	OSC 61673	*C. decurrens*	USA, Oregon	J. Trappe	–	–	–

*Herbaria, culture collections holding vouchers: CCCM = Canadian Centre for the Culture of Microorganisms; DAOM = Canadian National Mycological Herbarium; DAOMC = Canadian Collection of Fungal Cultures; E = Royal Botanic Garden Edinburgh; O = University of Oslo; PDD = New Zealand Fungarium, New Zealand Institute for Bioeconomy Science; UBC = University of British Columbia. **Single accessions are for LSU regions; second accession, if given, is for SSU.

In all work with sooty molds, it was essential to be attentive to the possibility that multiple fungal species were intermingled on the same leaf or twig surface. For morphological study, we carefully untangled small pieces of the sooty mold subiculum, searching for shiny, tapering, moniliform hyphae that characterize *Metacapnodium* as a genus, and paying close attention to any patterns of cell size, shape, and ornamentation that distinguish somatic hyphae of different species. We teased apart a small piece of sooty mold subiculum from each fresh or herbarium specimen in distilled water on a slide, then mounted the mycelia in 85% lactic acid on slides. Repeated observations from the same specimen were usually necessary to find reproductive structures among the dark colored, densely branching hyphae. We observed and measured any asexual or sexual structures using a Leitz DMRB DIC Microscope (Germany) and photographed structures using a Leica DFC420 Digital Color Camera (Germany).

### DNA extraction, amplification and sequencing

For a phylogenetic analysis of *Metacapnodium*, we extracted DNA from herbarium specimens, from fresh collections, and from the culture of the unidentified *Metacapnodium* sp. from Hawaii (DAOMC 252865, CCCM F128, corresponding to dried voucher UBC F33050). To harvest the target species from each specimen, we carefully selected small patches of mycelium under the dissecting microscope, moistening the subicula with a drop of water. We teased hyphae of *Metacapnodiaceae* apart from other sooty molds or other fungi using a dissecting needle, and placed them on a microscopic slide. Under the compound microscope, more than one fungal taxon was in some cases still evident. When this happened, we re-examined the target specimen under the dissecting microscope and selected several small patches from different parts of the subiculum. From each small patch, we teased hyphae with *Metacapnodium* spp. morphology apart and transferred them onto at least five slides, to be examined under the compound microscope. If a patch had an individual *Metacapnodiaceae* species or was at least dominantly occupied by one species, we harvested hyphae from the part of subiculum where the patch had originated for DNA extraction.

We extracted DNA from the mycelium using the DNeasy, Plant Mini Kit (QIAGEN Inc., Canada), following the manufacturer’s protocol. To check for consistent detection of the same species and to rule out laboratory contamination, we repeated extraction, amplification, and sequencing three times, unless the amount of subiculum in the herbarium specimens was limiting. PCR amplifications targeted ITS, SSU, LSU, and *ef1*–α regions using universal and specific primers (Table 2). We designed *Metacapnodium* specific ITS and *ef1*–α primers (Table 2) based on alignments including sequences from *Metacapnodium neesii* (Sugiyama et al. 2020) and from our cultured specimen from Hawaiian leaves (voucher UBC F33050). For PCR, each 25 μl reaction contained a master mix mixture of 10× Taq reaction buffer (2.5 μL), MgSO4 20 mM (2.5 μl), dNTPs 2 mM (2.5 μl), Taq DNA polymerase 5U/µL (0.125 µl) (Bio Basic Inc., USA), Milli-Q water (2.375 μl), 10 μM forward primer (1.25 μl), 10 μM reverse primer (1.25 μl), and genomic DNA diluted 1:1 or 1:9 in water. We performed amplifications as follows: 40 PCR cycles, 2 min at 94 °C (initial denaturation step), 10 s at 94 °C for denaturation, 20 s at about 53 °C for annealing, 30 s at 72 °C, and 7 min at 72 °C as a final elongation step. Annealing temperatures varied depending on primers. Amplifications of RPB2 using primers from Sugiyama et al. (2020) were unsuccessful so will not be described further.

**Table 2.** Primers used in this project.

Primer	Sequence	Locus	Reference
ITS1F (F)	CTTGGTCATTTAGAGGAAGTAA	*ITS*	(Gardes and Bruns 1993)
ITS1 (F)	TCCGTAGGTGAACCTGCGG	*ITS*	(White et al. 1990)
ITS3 (F)	GCATCGATGAAGAACGCAGC	*ITS*	(White et al. 1990)
BMB–CR	GTACACACCGCCCGTCG	*SSU*	https://sites.duke.edu/vilgalyslab/files/2017/08/rDNA-primers-for-fungi.pdf
CTB6 (F)	GCATATCAATAAGCGGAGG	*LSU*	(Garbelotto et al. 1997)
ITS2_MET (R)	GTTGCACCGAGGGTCGATC	*ITS*	This paper
ITS4 (R)	TCCTCCGCTTATTGATATGC	*ITS*	(White et al. 1990)
TW13 (=NL4) (R)	GGTCCGTGTTTCAAGACG	*LSU*	(O’Donnell 1993)
LR3_MET (R)	CCGTGTTTCAAGACGGGTC	*LSU*	This paper
LR0R (F)	ACCCGCTGAACTTAAGC	*LSU*	(Hopple Jr and Vilgalys 1994)
LR5 (R)	TCCTGAGGGAAACTTCG	*LSU*	(Hopple Jr and Vilgalys 1994)
LR8 (R)	CACCTTGGAGACCTGCT	*LSU*	(Hopple Jr and Vilgalys 1994)
nu–LSU–401 (R)	CCTTTCAACAATTTCACGT	*LSU*	(Döring et al. 2000)
EF1–983_FA (F)	GCYCCYGGYCATCGTGAYTTCAT	*EF1-α*	This paper
EF1–1567_FA (R)	ATGGGGACGAARGSAACRGHCTT	*EF1-α*	This paper

We purified PCR products using the QIAquick PCR Purification Kit (QIAGEN Inc., Canada) according to the manufacturer’s protocol. Each 10 μL sequencing reaction contained 2 μL of the DNA (10 ng/μL) (depends on the product length), 2 μL of 2.5 μM primer, 3 μL water, and 3 μL of diluted BigDye® Terminator v3.1 Sequencing Chemistry (Applied Biosystems, Canada). We set cycling conditions for sequencing reactions as follows: 1 min at 96 °C, followed by 25 cycles of 10 s at 96 °C, 5 s at 50 °C and 4 min at 60 °C. Using the DyeEx 2.0 Spin Kit (QIAGEN Inc., Canada) according to the manufacturer’s protocol, we purified the sequencing reactions and then submitted them to the Sequencing + Bioinformatics Consortium, UBC, Vancouver BC Canada for electrophoresis. We assembled and edited sequences using Sequencher 4.10.1 (Gene Codes Corp., USA).

### Phylogenetic taxon sampling

Sampling of the ITS regions included 16 newly determined *Metacapnodium* sequences, two previously determined *Metacapnodium* sequences, and two sequences (GenBank MH930326 and MW376663) that are highly similar to *Metacapnodium* species, even though they were not originally identified as such. We selected outgroups from order *Verrucariales* and family *Pleostigmataceae*, based on preliminary BLAST searches and on previously published studies (Gueidan et al. 2007; Gueidan et al. 2014; Chen et al. 2015; Crous et al. 2020; Cometto et al. 2023).

For analysis of the SSU, LSU, *ef1-α* and *rpb2* regions, we selected sequences to represent the diversity of classes *Dothideomycetes* and *Eurotiomycetes*, with members of *Leotiomycetes* included as the outgroup based on previous publications (Gueidan et al. 2007; Gueidan et al. 2014; Chen et al. 2015; Crous et al. 2020; Cometto et al. 2023) or on BLAST search results (Suppl. material 1). Most of the ribosomal RNA gene sequences were from GenBank. Most of the *rpb2* and *ef1-α* sequences were from whole-genome projects at the Joint Genome Institute (JGI) (Grigoriev et al. 2011; Grigoriev et al. 2014). Where possible, we used sequences from the same fungal isolate to represent each locus.

For many of the ascomycetes closest to *Metacapnodiaceae*, only partial LSU sequences were publicly available. In its sequenced genomes, JGI does not include transcriptome sequences for ribosomal RNA genes, but we sometimes found LSU sequences by searching JGI EST clusters or genome assemblies. Because LSU sequences are more easily accessed through GenBank, after finding LSU sequences in JGI, we used a sequence with a GenBank accession number instead of a JGI scaffold or EST cluster name, providing that the JGI and GenBank sequences were 100% identical over the aligned region (Suppl. material 1).

For alignments, we used MAFFT (http://mafft.cbrc.jp/alignment/server/index.html) with the L–INS–I (iterative refinement) method (Katoh et al. 2019). We edited the alignments manually using Mesquite version 3.81 (Maddison and Maddison 2023). For the two protein-coding genes, we applied minor edits to the alignment in Mesquite to maximize the similarity of aligned, predicted amino acids (Suppl. material 2). Initially, we analyzed each sequence region separately (Suppl. material 3: table SS2). The LSU sequences included unalignable regions, which we removed using GBlocks at its least stringent settings (Castresana 2000; Dereeper et al. 2008). We concatenated the SSU, LSU, *ef1-α* and *rpb2* regions for combined analysis, and phylogenetic analysis was carried out on data partitioned by region, and for *ef1-α* and *rpb2* regions, also by codon position (Suppl. material 3: table SS2).

### Phylogenetic analyses

To compute a matrix of uncorrected distances of the aligned ITS regions, we used the program distmat (http://emboss.open-bio.org/) (Rice et al. 2000). We analyzed individual and concatenated regions with raxmlGUI ver. 2.0.14 (Edler et al. 2021), after selecting appropriate evolutionary models (Darriba et al. 2020). We used 20 replicate searches with different starting trees and estimated support for branches using transfer bootstrap expectations (Zaharias et al. 2023) and Felsenstein branch support (Felsenstein 1985). We applied Bayesian analysis, running on MrBayes 3.2.7a, on Access, CIPRES (Miller et al. 2010) to the ITS alignment and to the concatenated alignment. Using 10,000,000 generations with 25% burn-in was sufficient for convergence of posterior probabilities, based on standard measures (Suppl. material 3: table SS2). We considered branches in the resulting trees to be well supported when they had at least 90% Felsenstein bootstrap support, 95% support from transfer bootstrap expectations, and a Bayesian posterior probability of 1.0.

## Results

### Species phylogeny

**Figure 2.**
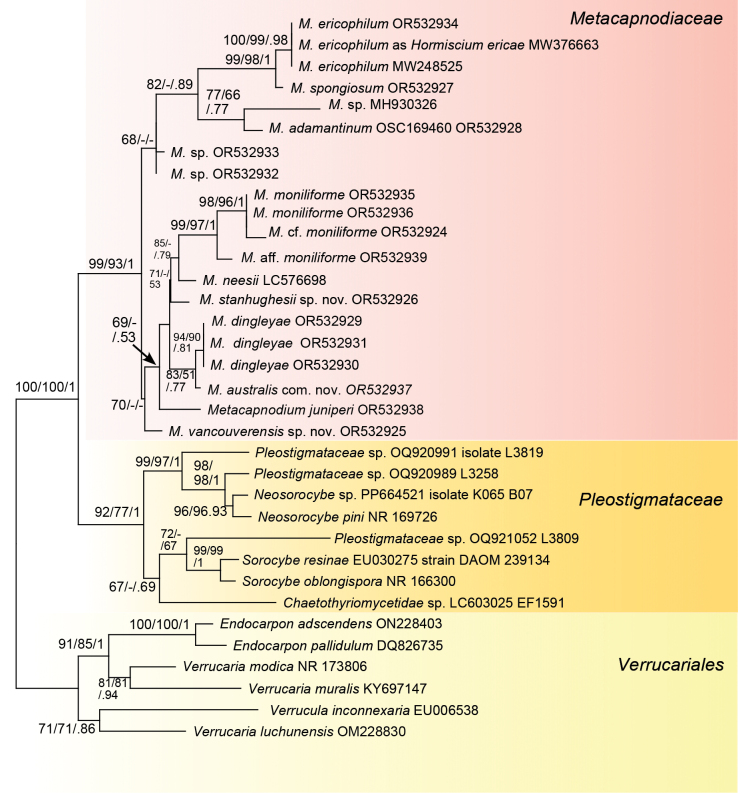
Maximum likelihood analysis of ITS sequences supports the monophyly of *Metacapnodium*. Numbers above branches are support values, as transfer bootstrap expectations, Felsenstein bootstrap number and Bayesian posterior probabilities. *Verrucariales* were selected as the outgroup.

We recognize 13 putative species of *Metacapnodiaceae*, based on ITS sequence divergence or morphology. The newly determined ITS sequences from 16 specimens represent at least 10 species, two described herein as new and 8 identified under existing species names. Other sequences may represent new species, but we lacked enough information to describe them here. Bayesian and likelihood analyses produced similar topologies, although support for branching order within *Metacapnodium* was low to moderate (Fig. 2). In BLAST searches, the closest taxa to *Metacapnodiaceae* were from *Neosorocybe*, *Sorocybe*, and *Pleostigmataceae* sp., all nested within *Pleostigmataceae*, or from *Verrucariales*. When the phylogeny was rooted with *Verrucariales*, then *Pleostigmataceae* and *Metacapnodiaceae* appeared as sister groups with moderate to strong support (Fig. 2). The monophyly of *Metacapnodiaceae*, including the family’s type species *Metacapnodium juniperi*, was strongly supported (Fig. 2).

The sequences of proposed new species *M. stanhughesii* and *M. vancouverensis* differed through the ITS regions from all other available sequences (Suppl. material 3: fig. S1). The ITS regions of *M. stanhughesii* differed from other *Metacapnodium* species by percentages varying from 2.2% (vs. *M. australis*) to 12.1% (vs. *M. ericophilum*MW376663). In *Metacapnodium vancouverensis*, the ITS regions similarly differed from other *Metacapnodium* species by percentages varying from 2.8% (vs. *M. australis*) to 11.6% (vs. *M. ericophilum*MW3766631) (Suppl. material 3: fig. S1).

**Figure 3.**
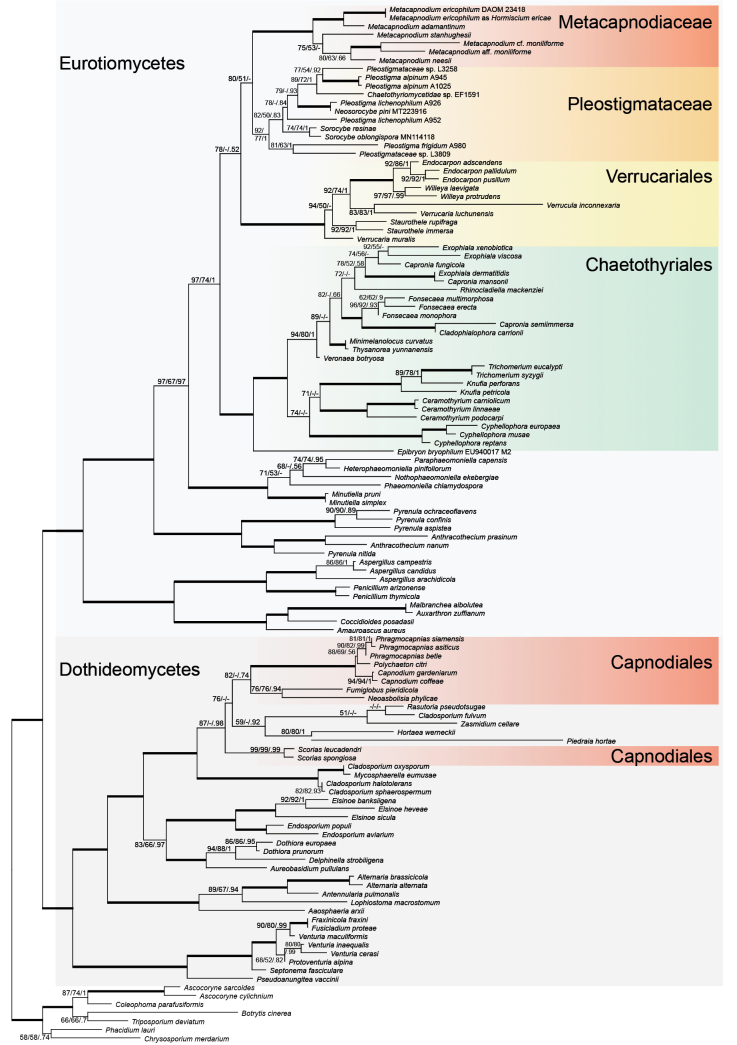
Maximum likelihood from four concatenated loci shows *Metacapnodiaceae* in class *Eurotiomycetes*, clustering weakly as sister to *Pleostigmataceae*, near clades *Verrucariales* and *Chaetothyriales*. Branches are in bold if strongly supported by transfer bootstrap expectations above 95%, Felsenstein bootstrap proportions over 90%, and Bayesian posterior probabilities of 1. Otherwise, numbers above branches indicate support and they are listed in the order above.

In the ITS phylogeny, neither of the proposed new species appeared closely related to any other species. Although morphologically similar to *M. moniliforme*, *M. stanhughesii* did not fall phylogenetically within the *M. moniliforme* clade. Its exclusion did not receive strong branch support, but it was consistent across loci. In the phylogenies from the ITS (Fig. 2), from concatenated gene regions (Fig. 3) and from the *ef1-α* (Suppl. material 3: fig. S4), *M. stanhughesii* fell outside of a clade comprising *M. moniliforme* and *M. neesii*.

*Metacapnodium ericophilum* appeared monophyletic with *M. spongiosum*OR532927 (Fig. 2). Two *M. moniliforme* specimen sequences along with *Metacapnodium cf. moniliforme*OR532924 formed a clade with strong support. The sequence from *M. aff. moniliforme*OR532939 appeared as sister to *M. moniliforme*. The *M. aff. moniliforme* specimen is only a small colony on a leaf of *Rhododendron* (Table 1), but future consideration of additional specimens may support recognizing this as a distinct species. Sequences of three specimens of *M. dingleyae* were monophyletic and formed a sister clade to *M. australis*, consistent with their morphological similarities (described below). Sequence OR532932 from specimen O F201613 and sequence OR532933 from DAOM 239041 may represent one or two new *Metacapnodium* species, but little material is available for either, and neither specimen displays the full range of characters that would distinguish them based on morphology. Sequence MH930326, from FLAS-F-64980 from the Florida Museum of Natural History also represents a probable new species. Although originally identified as *Scorias* sp., it bears typical *Metacapnodium* moniliform hyphae and a capnophialophora asexual form, consistent with its phylogenetic position within *Metacapnodium*.

Due to challenges with PCR amplification and sequencing from *Metacapnodium* herbarium specimens, *M. adamantinum*, *M. australis*, *M. moniliforme*DAOM 226251 and 234317, and *M.* sp. from DAOM 239041 and O F201613 had ITS region sequences of 150–181 bp, consisting mostly of the highly conserved, 156-base-pair 5.8S gene and just 21–85 aligned ITS2 sites. The limited sequence lengths may be related to specimen age; with collection dates from 1963 to 2003; these were among the oldest specimens sequenced (Suppl. material 3: fig. S1).

### Phylogenetic analysis of concatenated LSU, SSU, *ef1-α* and *rpb2* data

Analysis of the concatenated data for 122 taxa supported a position for *Metacapnodiaceae* within class *Eurotiomycetes*, subclass *Chaetothyriomycetidae* (Fig. 3). The monophyly of *Metacapnodium* received strong support. Although with limited support, *Metacapnodiaceae* appeared as the sister group to *Pleostigmataceae*, and together, *Metacapnodiaceae* and *Pleostigmataceae* formed the sister clade to *Verrucariales* (Fig. 3). Unexpectedly, but reflecting that *Pleostigmataceae* was only established in 2021 (Muggia et al. 2021), *Sorocybe resinae*, *Sorocybe oblongispora*, and *Neosorocybe pini* appeared interspersed among other taxa in *Pleostigmataceae*, rather than in *Chaetothyriales* as predicted by earlier classification (Seifert et al. 2007; Wijayawardene 2020) (Figs 2, 3).

Trees from individual SSU, LSU and concatenated gene regions were consistent in showing a clade containing *Metacapnodiaceae*, *Pleostigmataceae*, *Verrucariales*, and *Chaetothyriales* (Fig. 3, Suppl. material 3: figs S2, S3). However, relationships varied among trees from single gene regions, without strong bootstrap support for the conflicting topologies (Suppl. material 3). In the *rpb2* gene tree, for example, two species representing *Phaeomoniellales*, *Chaetothyriomycetidae* appeared as a sister clade to two *Verrucariales* (Suppl. material 3: fig. S5). The odd relationships did not receive support and the inconsistencies in branching order probably reflect the limited number of sequenced loci for members of the *Chaetothyriomycetidae*.

### Taxonomy

In this section, we describe and compare 14 putative species of *Metacapnodiaceae*, including two new species and four new combinations of *Metacapnodium*. If specimens had at least 15 spores or conidia of the same type, we give sizes of conidia and ascospores as minimum length, mean length, maximum length × minimum width, mean width, maximum width, plus or minus one standard deviation of the mean. The number of spores measured is given as ‘N =’. If fewer than 15 spores were observed, we provide the observed size range. We report typical width of hyphal cells from middle cells of branches or main stems, and width of the narrowest tip cells. To distinguish variations in the size and shape of conidia, the Q value (length/width ratio) is applied.

#### Metacapnodiaceae

Taxon classificationFungiCapnodialesMetacapnodiaceae

S. Hughes & Corlett (Hughes 1972)

CB60802B-6BFC-577E-B99C-7E82778E8FE4

##### Classification.

Order *incertae sedis*, *Chaetothyriomycetidae*, *Eurotiomycetes*.

##### Morphological characters.

**Figure 4.**
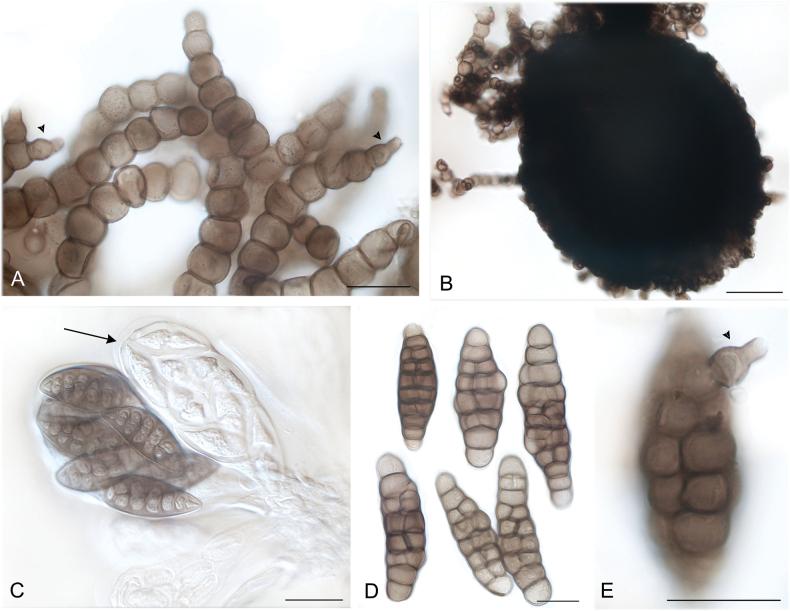
*Metacapnodium adamantinum* (OSC 169460). **A**. Moniliform hyphae with phialides (arrowheads); **B**. Ascoma; **C**. Asci (arrow); **D**. Ascospores; **E**. Ascospore bearing a phialide (arrowhead). Scale bars: 20 µm (**A, C–E**); 50 µm (**B**).

Dark, moniliform hyphae form a black or dark brown subiculum, usually on plant surfaces coated with insect honeydew. Hyphae taper smoothly towards their tips (e.g., Figs 1B, 4A). Cells are typically wider than long. Asexual states various but most species have a capnophialophora asexual form with dark-colored phialides bearing prominent collarettes.

*Metacapnodiaceae* comprises two genera:

*Metacapnodium* Speg. (Spegazzini 1918); IF3137

*Ophiocapnocoma* Bat. & Cif. 1963 (Batista and Ciferri 1963); IF3592.

##### Notes.

The number of septa per ascospore separates *Ophiocapnocoma*, with more than 13 transverse septa, from *Metacapnodium* with fewer than 13 septa (Sivanesan 1984). However, no sequence data are available for *Ophiocapnocoma*, and we are unable to test whether ascospore septation is diagnostic for clades.

###### Key to *Metacapnodiaceae* species considered here

Note: For descriptions and illustrations of named asexual forms mentioned in the key, refer to the introduction and to Fig. 1.

**Table:** 

1	Perithecia with ascospores present	**2**
–	Conidia present, ascospores not present	**6**
2	Ascospores more than 3-septate	**3**
–	Ascospores 3-septate	**5**
3	Ascospores dictyoseptate, usually with fewer than 13 transverse septa	**4**
–	Ascospores variable, some with more than 13 transverse septa, with occasional longitudinal septa, asexual form hormiokrypsis	***Ophiocapnocoma phloiophilia***
4	Ascospores with 8–13 transverse septa 2–5 longitudinal septa, capnophialophora is the only known asexual form	***M. adamantinum***
–	Ascospores with 5–6 transverse septa, 2–3 longitudinal septa, asexual forms, capnophialophora and highly branched, hyphal ‘gemmae’	***M. gemmiferum***
5	Only asexual form present is capnophialophora	***M. juniperi***
–	Capnobotrys or capnocybe asexual form present	**6**
6	Capnocybe present	**7**
–	Capnobotrys present	**9**
7	Conidia of capnocybe form usually 3-septate, occasionally 1–5 septate; internal cells of the synnema narrowly ellipsoidal to cylindrical, not subglobose or pyriform, phialides in capnophialophora asexual form not or only slightly constricted between venter and sub-cylindrical collarette (as in Fig. 1A)	**8**
–	Conidia in capnocybe asexual form regularly 3-septate; internal cells of the synnema inflated ellipsoidal, pyriform or subglobose in shape, phialides in capnophialophora asexual form strongly constricted between venter and a funnel-shaped collarette that widens at its tip; species known only from bark of *Nothofagus fusca* in New Zealand	***M. novae-zelandiae***
8	Conidia mostly 3-septate, occasionally 1–5 septate; forms dense subicula covering branches of conifers *Calocedrus*, *Chamaecyparis*, *Pseudotsuga* in western North America	***M. spongiosum***
–	Conidia 3-septate, rarely 1–5 septate; forms dense subicula covering branches of *Erica* species in Europe	***M. ericophilum***
9	Capnobotrys conidia multiseptated	***M. dingleyae***
–	Capnobotrys conidia 1-septate	**10**
10	Conidia markedly constricted at the septum, wall conspicuously warty	***M. australis***
–	Conidia not or only slightly constricted at the septum, wall without warts when septum first forms (may develop warts later)	**11**
11	Capnobotrys conidial proximal cell 1.2–1.5 × as long as distal cell, distal cell often cornute, the distal cornute protrusion superficially resembles proximal hilar appendix of a basidiospore	**12**
–	Capnobotrys conidia broad at base, gradually narrowing to tip; proximal cell averaging at least 1.5 times as long as distal cell and consistently, conspicuously wider; conidia not usually cornute	**13**
12	Hyphal cells up to 22 µm wide, light brown or brown, with fine warts; capnobotrys conidia often inequilateral, often cornute with light-colored apical protrusion, capnophialophora phialides with a funnel-shaped collarette that is constricted at its base and widest at its distal opening	***M. moniliforme* and *M. stanhughesii***
–	Hyphal cells up to 16 µm broad, dark olivaceous brown, with coarse, brown warts; capnophialophora phialides with a sub-cylindrical collarette that is at most slightly constricted at its base, not widening toward its distal opening	***M. atro-olivaceus***
13	Moniliform hyphae up to 30 µm wide, dark brown to brown, capnobotrys conidia proximal cells are ~2× the diameter of distal cells	***M. neesii***
–	Mature moniliform hyphae usually under 23 µm wide, dark olivaceous brown to blue green; capnobotrys conidia proximal cells ~1.5 × the diameter of distal cells	**14**
14	Mature cells up to 23 (–27 µm) wide, blue green, surface with fine warts; species known from small subicula on leaves of *Pieris japonica*	***M. vancouverensis***
–	Mature hyphal cells up to 18 µm wide, dark olivaceous brown; surface covered by coarse, dark-colored warts; capnophialophora phialides with a funnel-shaped collarette that is constricted at its base and widest at its distal opening (see fig. 19 N-R in (Hughes 1981)); species known from small subicula on dicot leaves, New Zealand, Hawaii, Chile	***M. pacificus***

#### Metacapnodium adamantinum

Taxon classificationFungiCapnodialesMetacapnodiaceae

S. Hughes & T.J. Atk.

F9BC60A6-1B73-5DE0-BF75-7481CA53BB98

Fig. 4

##### Specimen examined.

USA • Oregon, Coos County, vicinity of Eel Creek Campground, 2–4 km from Pacific Ocean Dunes, elevation: 5–25 m, 43.5883°N, 124.1900°W, on twigs of *Arctostaphylos* sp., Aug. 2000, B. McCune, OSC 169460.

##### GenBank accession number.

ITS, OR532928.

##### Description.

Subicula brown to dark brown, superficial. Mycelia of brown, moniliform, straight to curved hyphae, tapered towards the ends. Cells broader than long, (14–)20–25(–29) µm wide,12–22 µm long, distal, narrowest cells 10–12 µm wide.

**Sexual form**. Ascomata dark brown to black with short moniliform appendages scattered in the subicula, 450–500 µm long, 300–350 µm wide. Asci bitunicate, 8–spored, 120–160 × 30–40 µm. Ascospores hyaline to pale brown when immature and brown at maturity, diamond shaped, biseriate, in some cases germinating with phialides (Fig. 4E). Ascospores with 8–12 transverse septa, up to 5 longitudinal septa, and overall size (40)– 57.7–(90) +/– 11.3 × (15)–18.0–(27) +/– 2.4 µm, N = 30.

**Asexual form**. Capnophialophora – Phialides on ascospores or on hyphae, pale brown to brown, venter subglobose, 6–8 µm wide, collarette subcylindrical, 3–4 × 2–4 µm.

##### Hosts and geography.

The holotype grew on trunks of *Leptospermum scoparium* (*Myrtaceae*) in New Zealand and the host of specimen OSC 169460 was an *Arctostaphylos* sp. in Oregon, USA.

##### Notes.

Phylogenetically (Fig. 2), *M. adamantinum* forms a clade with *M. novae-zelandiae* and *M. ericophilum*, even though it apparently lacks the capnocybe form present in the two related species. Among taxa in *Metacapnodiaceae*, ascospores in two published species, *M. adamantinum* S. Hughes & T.J. Atk. and *M. gemmiferum* S. Hughes & T.J. Atk. are consistently dictyoseptate, distinguishing this from other species that have ascospores with transverse septa only. Hughes et al. (2012) mentioned an unpublished new *Metacapnodium* species on *Vaccinium* in Oregon, collected by Jeffrey Stone, which has dictyoseptate ascospores and an unknown relationship with *M. adamantinum*. *Metacapnodium adamantinum* can be distinguished from *M. gemmiferum* by having larger ascospores and more transverse and longitudinal septa (Hughes et al. 2012).

#### Metacapnodium atro-olivaceus

Taxon classificationFungiCapnodialesMetacapnodiaceae

(S. Hughes) Berbee & Aliabadi comb. nov.

2D35D849-820B-5348-93C7-E750C3B120D4

Index Fungorum: IF904052

Fig. 5

##### Basionym.

≡ *Capnobotrys atro-olivaceus* S. Hughes, [as ‘*atro-olivacea*’], New Zealand J. Bot. 19(2): 208 (1981).

##### Specimen examined.

New Zealand • South Island, Westland District, Granville Forest, Orwell Creek, on leaves of *Nothofagus fusca*, 2 Apr 1963, S.J. Hughes, from slides prepared by S.J. Hughes, from mixed collection, DAOM 106914 (*Capnobotrys atro-olivaceus* isotype).

##### Description.

The dark brown to black, velutinous subiculum of this specimen included abundant *Metacapnodium australis*. However, the several permanent slides associated with the specimen did contain *M. atro-olivaceus*. Mycelium of moniliform hyphae, olive-brown and densely verrucose throughout, warts coarse, prominent, dark olive-brown. Hyphae straight to curved, anastomosing, with lateral branching, tapering toward the ends, cells globose, as long as or broader than wide, to 16 μm wide, narrowing to 3.5–5 μm at the hyphal tips.

**Sexual form** unknown.

**Asexual forms**. Capnobotrys – Differentiated conidiophores not observed. Conidiogenous cells in irregularly arranged botryose clusters or in a unilateral series, brown and globose, elongating to become obpyriform during conidium formation. Conidia blastic, initially globose, then ovoid to ellipsoid, sometimes inequilateral, 11–13.5 × 7.5–9 µm, N = 8, Q = 1.5, olivaceous brown, smooth to verruculose, with one supramedial septum, slightly constricted at septum, proximal cell broader and slightly longer than the distal cell, with an inconspicuous scar that is located obliquely. Distal cells of conidia with apical wall thinning that is light olive brown in color.

Capnophialophora – Phialides olive-brown with a verrucous, subspherical venter 5–6 μm in width, and a subcylindrical collarette up to 3.5 × 2.5–3.5 μm. No phialidic conidia observed.

##### Hosts and Distribution.

Hughes (1981) reported this species from New Zealand, in bark of woody hosts and on leaves of dicots in several plant families including *Myrtaceae* and *Fagaceae*.

##### Notes.

**Figure 5.**
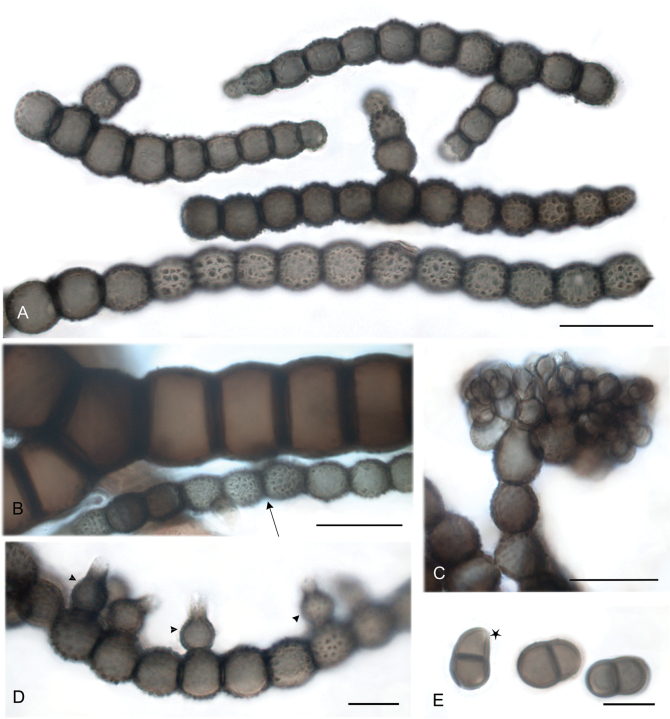
*Metacapnodium atro-olivaceus* (DAOM 106914). **A**. Moniliform hyphae; **B**. Olivaceous brown *M. atro-olivaceus* hypha (arrow), beneath much wider, bright brown *M. australis* hypha from the same mixed subiculum; **C**. Conidiogenous cell clusters of capnobotrys form; **D**. Phialides (arrowhead); **E**. Conidia of capnobotrys form, asterisk designates distal wall thinning. Scale bars: 20 µm (**A–C**); 10 μm (**D, E**).

*Metacapnodium atro-olivaceus* can be distinguished from other species with similar capnobotrys forms by its dark olive brown color, prominent, dark-colored verrucae, the subcylindrical rather than funnel-shaped collarettes on its phialides, and as exemplified by Fig. 5B, its mature hyphal cells are narrower than those of many other *Metacapnodium* species.

#### Metacapnodium australis

Taxon classificationFungiCapnodialesMetacapnodiaceae

(S. Hughes) Berbee & Aliabadi comb. nov.

B4E22545-7FF7-5B0B-A908-8DF7044FF099

Index Fungorum: IF903438

Fig. 6

##### Basionym.

≡ *Capnobotrys australis* S. Hughes, N.Z. Jl Bot. 19(2): 211 (1981).

##### Specimen examined.

New Zealand • South Island, Westland District, Granville Forest, Orwell Creek, on leaves of *Nothofagus fusca*., 2 Apr 1963, S.J. Hughes, from mixed collection, DAOM 106914 (*C. atro-olivaceus* isotype).

**Figure 6.**
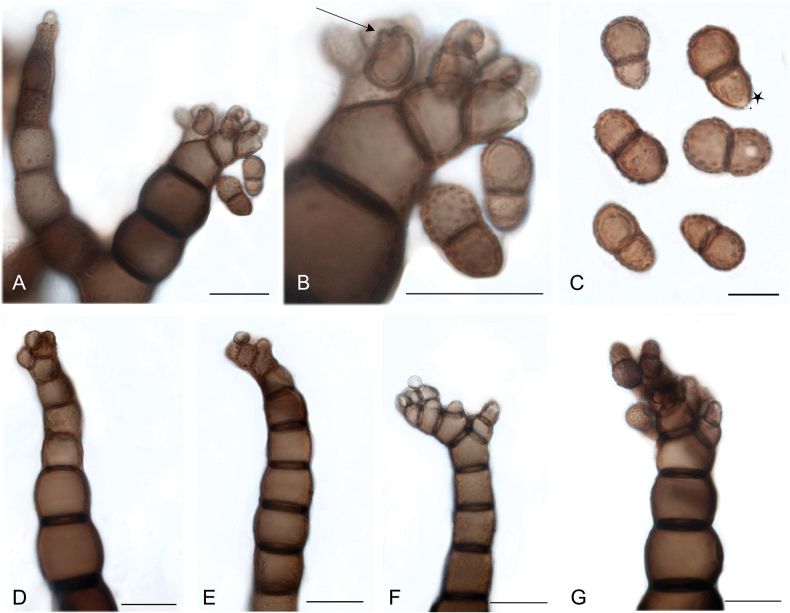
*Metacapnodium australis* (DAOM 106914), conidiogenous cells and conidia of capnobotrys form. **A**. Branched hyphae with conidiogenous cells; **B**. Enlarged view of conidiogenous cells from 6A showing conidium attachment at their proximal cells, arrow indicates denticles resulting from secession of conidia; **C**. Conidia, asterisk designates distal wall thinning; **D–G**. Hyphae with developing conidiogenous cells. Scale bars: 20 μm (**A, B, D–G**); 10 μm (**C**).

##### GenBank accession number.

ITS, OR532937.

##### Description.

Subiculum brown to dark brown to black, velutinous, intermingled with other sooty molds. Although DAOM 106914 is an isotype of *C. atro-olivaceus*, the specimen consists predominantly of *M. australis*. Mycelia of brown doliiform to moniliform hyphae, usually moniliform when mature, coarsely or sparsely verrucose throughout, hyphae are often curved, featuring branches that originate at right angles, then curve upwards, tapered towards their tips. Cells broader than long, 23–32 µm wide,18–25 µm long, narrowing to 6–7 µm wide at the hyphal tips.

**Sexual form** Unknown.

**Asexual forms**. Capnobotrys – Differentiated conidiophores not observed. Conidiogenous cells often unilateral, in clusters at the ends of erect hyphae or on sub-terminal branches, on terminal or penultimate cells or cells just below, bearing denticulate scars where successive conidia budded out, then seceded. Conidia initially subglobose, elongating to ovoid, coarsely verrucose, becoming 1–septate, constricted at supramedial septum. Conidia (12.0)–15.9–(18.5) +/– 2.4 × (7)–9–(–12) +/– 1.6 µm, N = 20, Q = 1.76. Proximal cell length is ~1.4 × distal cell length; proximal cell width ~1.3× distal cell width. Proximal cells subglobose, 9.1 +/– 1.4 × 9 +/– 1.6 µm; distal cell 6.8 +/– 1.2 × 7.2 +/– 1.33 µm; distal cells tapering towards tip, at tip, cell wall thins and is lighter brown in color, possibly a germ pore.

Capnophialophora – Not observed.

##### Host and distribution.

On bark of *Nothofagus* spp. from New Zealand (Hughes 1981).

##### Notes.

The mature hyphae of *M. australis* and *Capnobotrys atro-olivaceus* that are intermingled mycelia in the subiculum of DAOM 106914 differ in diameter and color. Hyphae of *M. australis* are up to 29 µm wide and are brown without olivaceous tints, while mature hyphae of *C. atro-olivaceus* are up to 16.2 µm wide and are “dull, dark olivaceous brown” (Hughes 1981).

In the ITS phylogeny, *M. australis* appears as sister to *M. dingleyae* (Fig. 2). Hughes (1981) distinguishes *M. australis* from three similar species: *Capnobotrys laterivecta*, *C. paucisporus*, and *M. dingleyae* that share a capnobotrys form and curved hyphae with upwardly curving or straight branches that arise at right angles, and more or less doliiform component cells. Most similar to *M. australis* is *C. laterivecta*, which can be distinguished from *M. australis* by its larger conidia and by the unusual attachment of its conidia by their long sides, just below their septa, to conidiogenous cells (Hughes 1981). *Metacapnodium australis* has smaller conidia and a larger number of conidiogenous cells at hyphal tips compared to *C. paucisporus*. *Metacapnodium dingleyae* can be easily distinguished by the presence of conidia with more than one septum (Hughes 1981). The prominent constrictions at the septa and coarse warts on capnobotrys conidia of *M. australis* help distinguish this species from others with capnobotrys forms, including *M. moniliforme*.

#### Metacapnodium dingleyae

Taxon classificationFungiCapnodialesMetacapnodiaceae

S. Hughes

07EB3FBF-C544-550C-9E89-1574BDD4BB5B

Fig. 7

##### Specimens examined.

Norway • Ålesund, Skinstadreset, on *Taxus baccata* L., 21 Jan. 1999, D. Holtan, O F65377; • Ålesund, Skodje, on *Taxus baccata* L., 21 Jan. 1999, D. Holtan, O F65671; • Ålesund, Fyllingsfjellet, Skodje, on *Taxus baccata* L., 8 May 1999, D. Holtan, O F66918; • Ålesund, Øvrestølen, on *Taxus baccata* L., 30 April 2001, D. Holtan, O F171421; • Ålesund, Glømmesætra, on *Taxus baccata* L., 30 April 2001, D. Holtan, O F171422; • Ålesund, Sætrevegen, on *Taxus baccata* L., 1 May 2001, D. Holtan, O F171424; • Ålesund, Sætreheia, on *Taxus baccata* L., 24 Nov. 2002, D. Holtan, O F201624; • Ålesund, Storberget, on *Taxus baccata* L., 25 Feb. 2003, D. Holtan, O F201720, O F201727; • Ålesund, Opskar, on *Taxus baccata* L., 2 Mar. 2003, D. Holtan, O F201744; • Hjelmeland, Randøy, on *Taxus baccata* L., 15 Mar. 2003, J.I. Johnson, O F281492; • Sykkylven, Birkeneslia, on *Taxus baccata* L., 26 Jul. 2010, D. Holtan & P.G. Larsen, O F293899.

**Figure 7.**
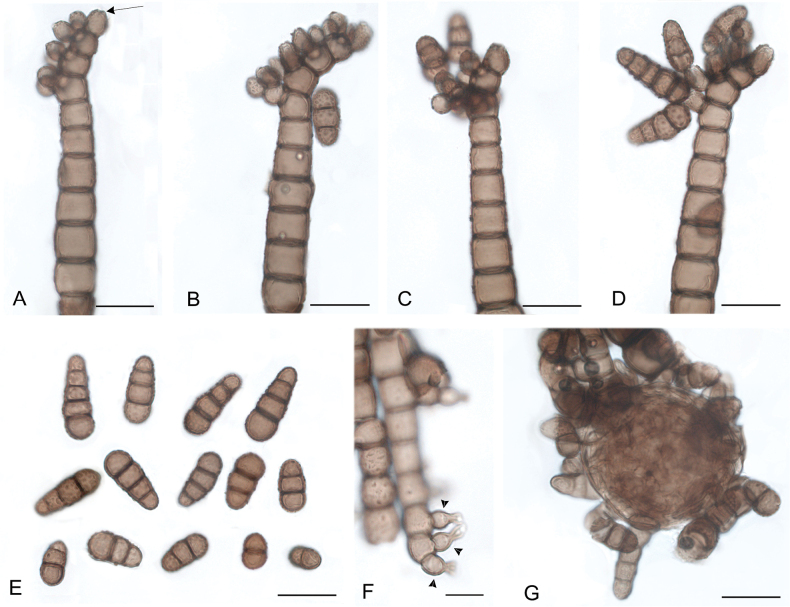
*Metacapnodium dingleyae* (O 201720). **A–E**. Capnobotrys form; **A, B**. Hyphae with conidiogenous cells; **C, D**. Initial and mature conidia; **E**. Conidia; **F**. Phialides (arrowheads); **G**. Ascoma. Scale bars: 20 μm (**A–E, G**); 10 μm (**F**).

##### GenBank accession numbers.

Collection O F201720 (ITS, OR532929); Collection O F281492 (ITS, OR532930); Collection O F293899 (ITS, OR532931).

##### Description.

Subicula thick, dark brown to black, superficial. Mycelia of moniliform to doliiform hyphae, brown when mature, smooth to slightly verrucose throughout, and verrucose distally, tapered towards their ends. Cells broader than long, up to 30 µm wide, narrowing to 6 µm at hyphal tips.

**Sexual form**. Ascomata scattered to crowded in some specimens, dark brown, semi–immersed in subicula, 120–200 × 90–150 µm, bearing moniliform appendages. No mature asci and ascospores observed.

**Asexual forms**. Capnobotrys – No distinctive conidiophores observed. Conidiogenous cells arise variously, unilaterally from cells at and just below the hyphal tip, in clusters at the tips of erect hyphae. Conidiogenous cells ovoid, brown, verruculose, bearing denticulate scars where successive conidia budded out, then seceded. Conidia ovoid, walls up to 2 µm thick, up to five septate, slightly constricted at the septa, deeply verrucose, brown to dark brown. Conidium size increases with number of septa: aseptate conidia (6)–8.5–(10.5) +/– 1.7 × (5)–6.8–8) +/– 1.5 µm, N = 20, the two-septate conidia (17)–18.6–(20) +/– 1.7 × (8)–9–(11) +/– 1.0 µm, N = 26, the less common five-septate conidia (38.0)–41.0–(44.0) +/– 3.2 × (10.0)–11–(12.0) +/– 0.7 µm, N = 3. Cells wider than long, proximal cells usually wider and longer than distal cells.

Capnophialophora – Phialides on distal cells of hyphae and (not illustrated) on capnobotrys conidia, light brown to brown, verruculose, subspherical venters 5–6 µm wide; collarettes deeply constricted at junction with venter, funnel–shaped, up to 4 µm long, opening to 2 µm wide. No phialidic conidia observed.

##### Hosts and distribution.

Reported from New Zealand on living trunks of woody gymnosperms: *Phyllocladus trichomanoides*, *Prumnopitys taxifolia*, *Dacrydium cupressinum* (Hughes 1981). Possibly also widespread on *Taxus baccata* (*Taxodiaceae*) in Europe: specimens from *Taxus* sp. from Norway examined here, also reported from England (Ellis 1976), Ireland (Hughes 1981) and Italy (Karadelev et al. 2017).

##### Notes.

The ITS phylogeny places three specimens (O F201720, O F281492, O F293899) in the same clade with strong support. The *M. dingleyae* clade appears as sister to *M. australis* with 51–83% bootstrap support (Fig. 2). Morphologically, *M. dingleyae* can be distinguished from other species producing a capnobotrys form by their conidia with more than one septum.

#### Metacapnodium ericophilum

Taxon classificationFungiCapnodialesMetacapnodiaceae

(Link) D. Hawksw. & S. Hughes

9B8DFF50-A0EB-541F-8A72-515943A2B2D0

Fig. 8

##### Specimen examined.

Spain • Comunidad de Madrid: Sierra de Guadarrama, Parque Regional de la Cuenca Alta del Manzanares, La Pedriza de Manzanares, on *Erica arborea* L., 22 Nov. 2004, D.L. Hawksworth, DAOM 234182a.

**Figure 8.**
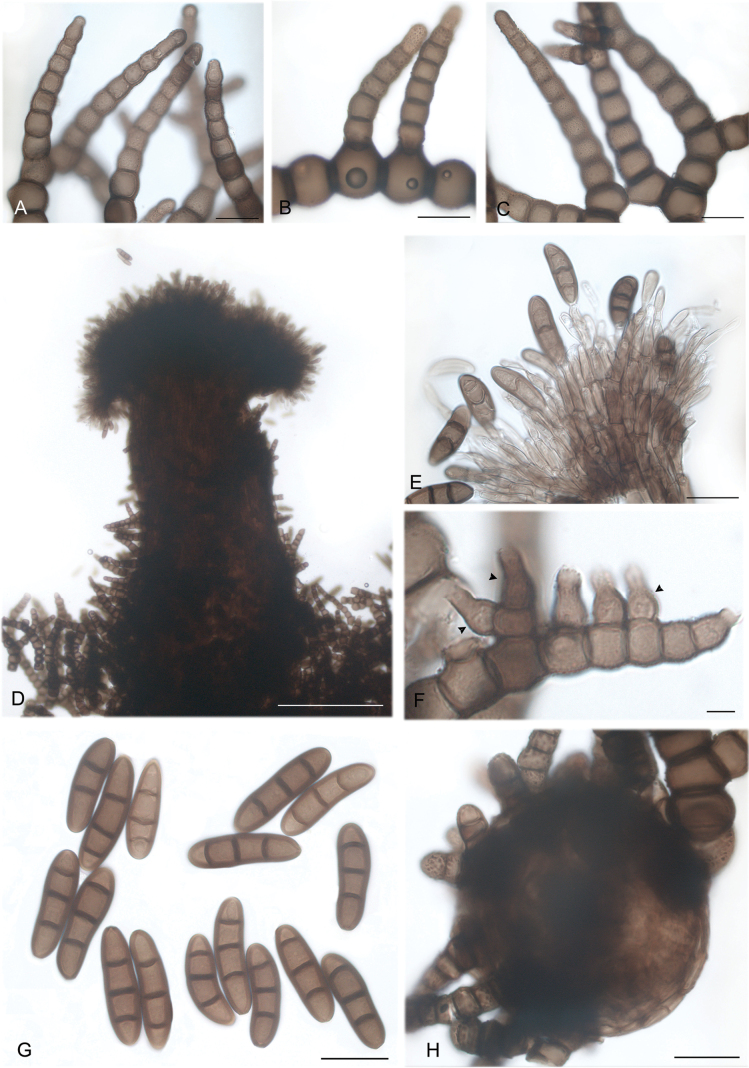
*Metacapnodium ericophilum* (DAOM 234182). **A–C**. Moniliform hyphae; **D, E**. Capnocybe form, synnema; **D**. Overview; **E**. Penicillate conidiogenous cells bearing conidia; **F**. Phialides with subcylindical collarettes (arrowheads); **G**. Conidia; **H**. Ascoma initial. Scale bars: 20 µm (**A–C, E, G–H**); 5 µm (**F**); 200 µm (**D**).

##### GenBank accession numbers.

ITS, OR532934; LSU, PP140683.

##### Description.

Subicula blackish, superficial, developing confluent thick and cracked cushions extending over host branches. Mycelia of doliiform and moniliform, brown to dark brown hyphae. Hyphae branching, straight, surface verrucose, tapering towards their tips. Hyphal cells to ~27 µm in diameter when mature, 5 µm in diameter at tip.

**Sexual form**. Ascomata scattered to crowded, dark brown to black, globose, some subglobose, semi–immersed in subicula, 90–180(–200) × 90–180 µm, bearing moniliform appendages. No mature asci or ascospores observed.

**Asexual forms**. Capnocybe – Synnemata up to 800 µm high, sparsely distributed throughout the subicula, their basal hyphae of ellipsoidal to globose cells, stipes 350–600 × 50–150 µm. Stipes of compact, fasciculate hyphae with cylindrical to ellipsoidal cells tapering to 6 µm towards the apex. Heads (capitula) of synnemata up to 300 µm wide. Terminal conidiogenous cells cylindrical, 10–16 × 5.5–7 µm, producing successive conidia blastically at their tips. Conidia fusiform, (2–) 3–septate, dark brown, (25)–33–(39) +/– 2.7 × (8)–9.2–(12) +/– 0.7 µm, Q = 3.6.

Capnophialophora – Phialides light brown to brown, subspherical venters 5–6 µm wide, near-cylindrical collarettes up to 4 µm by 2–4 µm. No phialidic conidia observed.

##### Hosts and distribution.

Hawksworth and Boluda (2020) reported the species from Spain and Portugal, commonly on *Erica arborea*, also on *Erica australis*, and perhaps present on other species of *Erica*.

##### Notes.

Phylogenetically, this species appears as sister to *M. spongiosum* in the ITS tree with 84–85% bootstrap support (Fig. 2) and ITS sequences of the two species only differ by about 2%. Both species produce capnocybe forms with similar, penicillate conidiogenous cells and their conidia are of similar shape, size and septation. Phialides in both species are somewhat unusual for *Metacapnodium* in that collarettes are subcylindrical rather than funnel-shaped. The two species differ in known geographical and host ranges: *M. ericophilum* forms dense subicula covering branches of *Erica* in Spain and Portugal, while *M. spongiosum* forms dense subicula covering branches of *Calocedrus decurrens* from California (Hughes 1966), and from Oregon, based on our re-identification of specimen OSC 135428 (Table 1).

Hughes (1976) recognized the similarity of these two species but considered *Antennaria ericophila* Link, the basionym of *M. ericophilum*, to be a *nomen dubium* due to lack of clarity about its application. Hawksworth and Boluda (2020) resolved the application of the name, epitypified and lectotypified *M. ericophilum*, and sequenced the ITS region of DAOM 234182, the same specimen we used for sequencing ITS and ef1-a gene regions. *M. ericophilum* was abundant on *Erica arborea* but not on nearby junipers, and Hawksworth and Boluda suggested this may indicate host specificity but alternatively, it could reflect a fungal requirement for honeydew from particular scale insects, and only indirectly for the plant genus.

#### Metacapnodium juniperi

Taxon classificationFungiCapnodialesMetacapnodiaceae

(W. Phillips & Plowr.) Speg.

B983455C-42D0-5E1E-AECF-13188BD54B56

Fig. 9

##### Specimen examined.

Scotland • VC–96 – East Inverness–shire, On *Juniperus* sp., 15 Feb. 2023, leg. Sophie Furze, E01043363.

##### GenBank accession number.

ITS, OR532938.

##### Description.

Subicula dark brown to black and spongy, 0.3 to 14 mm in diameter according to Hughes (1972). Mycelium of moniliform, brown to dark brown hyphae, hyphae mainly smooth throughout but verrucose at the distal parts, anastomosing, branched, straight or curved, tapering to their tips. Cells broader than long, to 27 µm wide, narrowing to 6 µm wide at hyphal tips.

**Figure 9.**
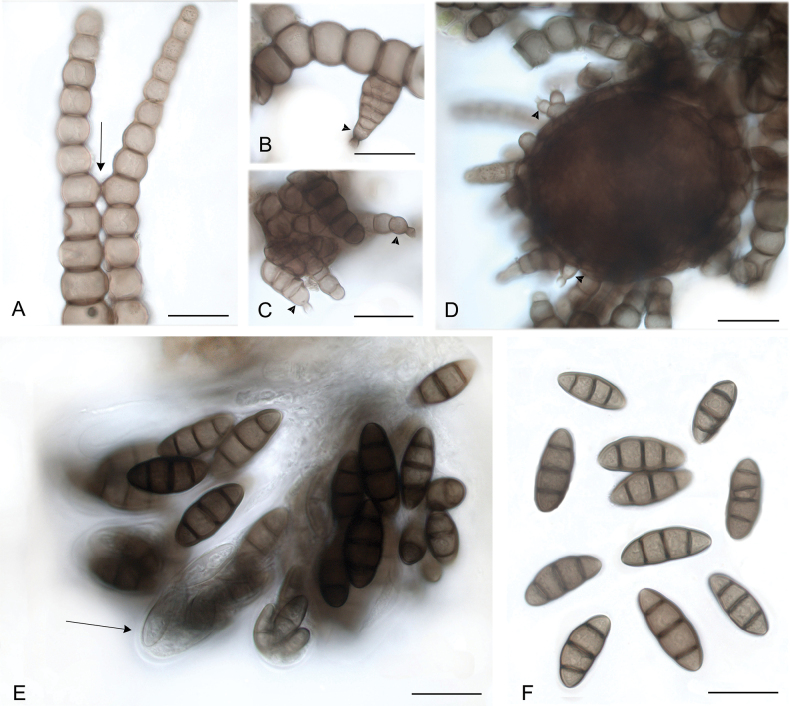
*Metacapnodium juniperi* (E01043363). **A**. Moniliform hyphae, undergoing anastomosis (arrow); **B, C**. Phialides (arrowheads) on ascoma initials; **D**. Ascoma bearing phialides (arrowhead) on appendages; **E**. Asci (arrow); **F**. Ascospores. Scale bars: 20 µm.

**Sexual form**. Ascomata are abundant, semi-immersed in the subiculum, ostiolate, dark brown to black, 120–210 × 90–130 µm. Asci bitunicate, ellipsoid, 58–82 × 21–27 µm, 8–spored. Ascospores 3–septate, dark brown when matured, ellipsoid, sometimes slightly constricted at the septa, (20)–22.9–(26) +/– 2.4 × (8)–9–(11) +/– 1.0 µm, N = 45.

**Asexual form**. Capnophialophora –The only known asexual form for the species. Phialides appear on hyphae, ascomata initials, ascomata appendages, and (not illustrated, but see fig. 6J in Hughes (1972)), on ascospores. Venters light brown to brown, hemispherical to subspherical, 4.5–7 µm wide; collarettes pale brown, funnel-shaped to ellipsoidal 2.5–3.5 µm long with narrow basal constriction, 2.5–4 µm wide at their openings. No phialidic conidia observed.

##### Hosts and distribution.

*Metacapnodium juniperi*, the type species of *Metacapnodium*, is known from small subicula growing over juniper twigs in Scotland.

##### Notes.

In 2023, Sophie Furze kindly made a specimen available to us by collecting the specimen from East Inverness–shire, Scotland, a site close to the type locality in Scotland, from juniper, the type host, and then depositing the specimen in RBGE. Its sequenced ITS region falls among other species of *Metacapnodium*, as expected for the type species of its genus. (Fig. 2).

#### Metacapnodium moniliforme

Taxon classificationFungiCapnodialesMetacapnodiaceae

(L.R. Fraser) S. Hughes

CA184EF0-6723-513E-8F83-E5304E29BA00

Fig. 10

##### Specimens examined.

Usa • Hawaii, On *Cheirodendron trigynum*, Jun. 1990, C. Inada, DAOM 234317; Australia, New South Wales, on unidentified leaves, 17 Aug. 1999, K. Seifert, DAOM 226251.

##### GenBank accession numbers.

Collection DAOM 234317 (ITS, OR532936); Collection DAOM 226251 (ITS, OR532935).

##### Description.

Subicula dark brown to black, effuse, superficial on leaves or twigs, typically less than 1 cm in diameter although sometimes becoming confluent. Mycelium of moniliform hyphae. Hyphae pale brown to brown, when mature, finely verrucose, deep constrictions between cells. Hyphae are erect, curved, smooth to finely verrucose, branching, then growing upwards, narrowing towards their tips. Most cells as broad as or broader than long, subglobose to doliiform, when mature up to 21 µm wide, tip cells narrowing to 5 µm.

**Figure 10.**
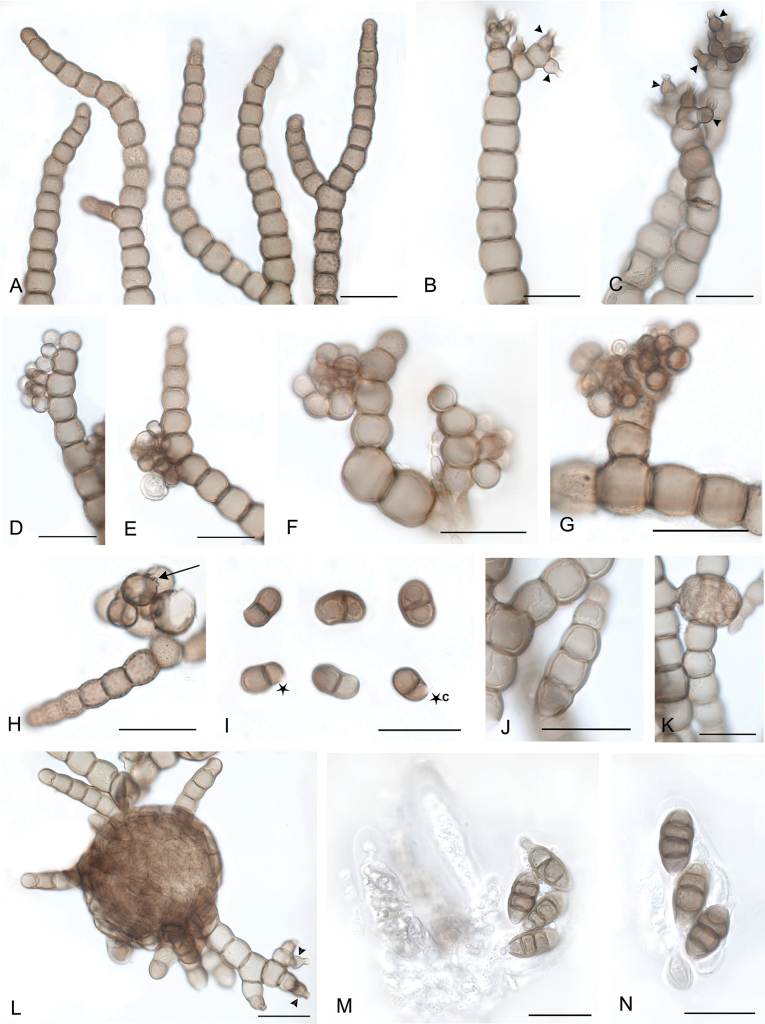
*Metacapnodium moniliforme* (DAOM 226251). **A**. Moniliform hyphae; **B, C**. Moniliform hyphae with phialides (arrowheads); **D–I**. Capnobotrys form; **D–G**. Conidiogenous cells; **H**. Conidiogenous cell with denticles (arrow) from successive production and secession of conidia; **I**. Conidia, asterisks designate distal wall thinning; c designates a cornute conidium; **J**. Conidium of capnosporium form; **K**. Ascoma initial; **L**. Ascoma bearing phialides (arrowheads) on appendages; **M**. Immature and mature asci; **N**. Ascospores. Scale bars: 20 µm.

**Sexual form**. Ascomata scattered, semi-immersed in subicula, dark brown, globose (immature), subglobose to obovoid, (50–)70–180 µm high and 50–120 µm wide, ostiolate, having straight to curved appendages (Fig. 10K, L). Asci bitunicate, 8–spored, ellipsoid to clavate, 50–70 × 12–25 µm. Ascospores irregular or biseriate within asci, ellipsoid, light brown to brown, mainly 3(–4) septate, (17)–19.8–(23) +/– 2.6 × (8)–8.75–(10) +/– 0.7 µm (DAOM 226251).

**Asexual forms**. Capnobotrys – No distinctive conidiophores observed. Conidiogenous cells unilateral or in botryose clusters at sides or tips of moniliform hyphae, globose to subglobose, brown to dark brown, bearing denticulate scars where successive conidia budded out, then seceded. Conidia brown, initially smooth walled, subglobose, elongating to become ellipsoid ovoid to allantoid, often cornute and inequilateral, with a single median or supramedial septum. Conidia (9)–14–(20) +/- 3.4 × (5)–7.7–(11) +/- 1.9 µm, Q = 1.8 (DAOM 226251) or (9)–12.3–(14.5) +/- 1.7 × (7)–7.9–(9) +/- 0.8 µm, Q = 1.6 (DAOM 234317, not illustrated). Proximal cells 8.0 +/– 1.9 × 7.7 +/– 1.9 µm, longer and wider than distal cells 6.1 +/– 1.2 × 6.3 +/– 1.9 µm. Proximal cells sometimes with inconspicuous, oblique scar from secession from basal or diagonal attachment to their conidiogenous cell. Distal cell with an apical patch of thinner, paler wall that sometimes protrudes, resembling the proximal hilar appendix of a basidiospore.

Capnophialophora – Phialides single or in small clusters, with light brown to brown subspherical venters 4–6 µm wide, bearing a funnel-shaped collarette 2.5–4.5 µm long with a constriction at its base, 2–3 µm wide at its opening.

Capnosporium – Rarely observed, but for examples of sizes, a conidium with two septa was 25 × 12 µm; one with three septa was 32 × 9 µm, and one with four septa was 36 × 12 µm (DAOM 226251).

##### Hosts and distribution.

Reported on small twigs and leaves of varied plants including ferns and dicots. Widely distributed according to Hughes (1981); known from New Zealand, Australia, Chile, and perhaps elsewhere if *M. cf. moniliforme*UBC F33050 from Hawaii, and *M. aff. moniliforme* E 01043362 from Scotland could all be considered conspecific.

##### Notes.

The ITS phylogeny shows that the two specimens identified as *M. moniliforme*, DAOM 234317, DAOM 226251, *M. cf. moniliforme*UBC F33050 (described below) and *M. aff. moniliforme* E 01043362 form a clade with 97% bootstrap support (Fig. 2). The average sizes of the capnobotrys conidia varied with lengths from 10 to 14 µm. Hughes (1981) reported that conidia may almost double in length, walls becoming verrucose, at the time of germination.

The *M. moniliforme* group and *M. neesii* appear as sister groups in trees from ITS, concatenated genes, and *ef1-α*, although without strong statistical support. Conidiogenous cells of capnobotrys in *M. neesii* and *M. moniliforme* are similar in shape, in arrangement, and in the oblique positioning of conidia on the conidiogenous cell. Conidia in both are 1-septate. However, in *M. neesii*, the distal cell of a conidium is often less than half the length of the proximal cell (Hughes 1979) while in *M. moniliforme*, the distal cell is between 2/3 and 3/4 of the length of the proximal cell. According to Hughes (1981), the capnobotrys conidia of *M. guava* are smaller but otherwise similar in shape to those of *M. moniliforme*. However, sizes of conidia of *M. guava* given by Hughes (1981) fall within the range of sizes of conidia *M. moniliforme* measured here. Hughes (1981) suggested that *M. guava* capnobotrys conidia do not develop the cornute morphology seen in *M. moniliforme*. To resolve the differences between the two species, a combination of comparative morphology and barcode sequencing of additional collections would be useful.

#### Metacapnodium cf. moniliforme

Taxon classificationFungiCapnodialesMetacapnodiaceae

(L.R. Fraser) S. Hughes

7F56F133-71A3-5186-AD2F-6750A204A2C9

Fig. 11

##### Specimens examined.

USA • Kauai’i, Pihea Trail, at the Pu’u O Kila lookout point, Alt: 1260 m, 22.1474°N, 159.6311°W, on leaves of *Metrosideros cf. polymorpha*, 23 April 2016, J. Dee, UBC F33050, cultured as DAOMC 252865, CCCM F128.

##### GenBank accession numbers.

ITS, OR532924; SSU, PP836272; LSU, PP140680; *ef1-α*, OR820948.

##### Description.

Subicula dark brown to black, effused, superficial. Mycelium of moniliform hyphae, hyphae pale brown to brown when mature, smooth to finely verrucose, with deep constrictions at septa. Hyphae erect, straight to curved, hyphal branches grow upwards, narrow towards their tips. Cells broader than long, subglobose to doliiform, 15–25(–28) µm wide, narrowing to 6–10 µm at hyphal tips.

**Sexual form** unknown.

**Asexual forms**. Capnobotrys – Differentiated conidiophores not observed. Conidiogenous cells in whorls or semi–whorled clusters at the sides or tips of moniliform hyphae, globose to subglobose, brown to dark brown, bearing denticulate scars where successive conidia budded out, then seceded. Conidia brown, smooth walled, allantoid, ovoid, or ellipsoid in shape, brown, smooth walled, sometimes cornute and inequilateral, with a single median or supramedial septum, not or only slightly constricted at septa. On leaves, conidia (9)–10.8–(14) +/– 1.7 × (5)–7–(10) +/– 1.8 µm, N = 15, Q = 1.57, proximal cell 6.5 +/– 0.8 × 7.0 +/– 1.84 µm; distal cell 4.3 +/– 0.96 × 5.9 +/– 2 µm. Proximal cells 1.57× as long as distal cells and 1.2× as wide. A few much larger, dark brown, verrucose, thick-walled conidia also present, 15–18 × 10–11 µm. Conidia in culture similar but slightly narrower, (8)–10–(12) +/– 1.0 × (5)–5.8–(7) +/– 0.6 µm, N = 30, Q = 1.8. Proximal cells longer and wider than distal cells; proximal cells 6.0 +/– 0.8 × 5.8 +/– 0.6 µm, distal cell 4.2 +/– 0.4 × 4.1 +/– 0.4 µm. Proximal cells from leaves and cultures sometimes with an inconspicuous, oblique scar resulting from secession from basal or diagonal attachment to a conidiogenous cell; distal cells bear an apical wall thinning, light brown in color, sometimes protruding to resemble a basidiospore’s hilar appendix.

**Figure 11.**
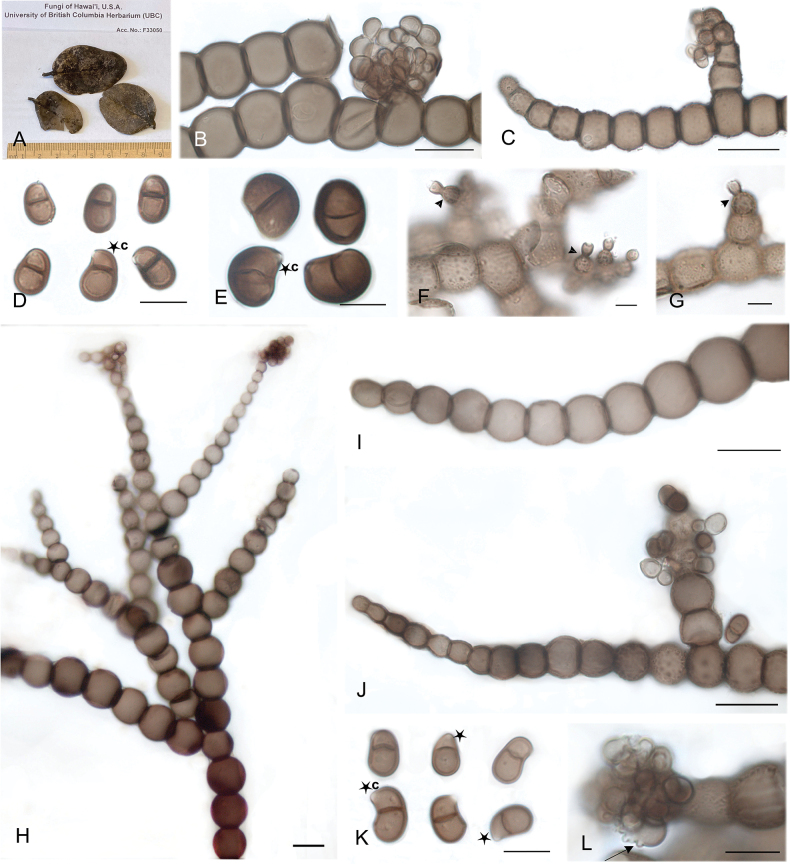
*Metacapnodium cf. moniliforme* (UBC F33050). **A–G**. Wild collected material; **A**. leaves of *Metrosideros* sp. covered by mycelia of *M. cf. moniliforme* and other sooty molds; **B–E**. Capnobotrys form; **B, C**. Moniliform hyphae with clusters of conidiogenous cells and conidia; **D, E**. Conidia of two size classes from the same subiculum, asterisks designating distal wall thinning; c designating cornute conidia; **F, G**. phialides (arrowheads); **H–L**. *Metacapnodium cf. moniliforme* in culture (UBCCCCM F128); **H**. Moniliform hyphae with terminal clusters of conidiogenous cells (capnobotrys form); **I**. Moniliform hypha; **J–L**. Capnobotrys form; **J**. Conidiogenous cells and conidia; **K**. Conidia, asterisks designate distal wall thinning; c designates cornute conidium; **L**. Conidiogenous cell with denticles (arrow) from successive production and secession of conidia. Scale bars: 20 µm (**B, C, H–J**); 10 µm (**D, E, K, L**); 5 µm (**F, G**).

Capnophialophora – (Fig. 11F, G) Phialides, found on leaves but not in cultures, on tips or sides of hyphae, with a light brown to brown, subspherical venter 4–6 µm wide; bearing a funnel–shaped collarette up to 3 µm long, constricted at its base and 2 µm wide at its opening.

##### Host and distribution.

Known only from leaves of *Metrosideros cf. polymorpha* from Hawaii. However, additional basic systematic research may place this slightly divergent specimen in *M. moniliforme*, a species with a broad host range.

##### Notes.

This specimen’s ITS sequence clusters with the other two *M. moniliforme* specimen sequences with 96% or more bootstrap support but is 1.7 to 2.5% different from the other *M. moniliforme*ITS sequences. Morphologically, it is similar to *M. moniliforme*. However, no *Capnosporium* form was observed.

#### Metacapnodium aff. moniliforme

Taxon classificationFungiCapnodialesMetacapnodiaceae

(L.R. Fraser) S. Hughes

08461097-D5AD-57DC-8419-4B78C36BFE67

##### Specimen examined.

Scotland • VC–98–Argyllshire– Benmore Botanic Garden, on *Rhododendron* sp., 13 Mar 23, Rebecca Yahr, E 01043362.

##### GenBank accession numbers.

ITS, OR532939; LSU, PP140684.

##### Description.

Like *M. moniliforme*, this specimen has both a capnobotrys and a capnophialophora form.

**Sexual form** unknown.

**Asexual form**. Capnobotrys – Mode of conidiogenesis, conidium shapes similar to two DAOM*M. moniliforme* samples described above. Conidium sizes (9)–12.45–(17) +/– 1.7 × (6)–8–(10) +/– 1.2 µm, N = 30, Q = 1.5 slightly larger than two DAOM*M. moniliforme* samples.

##### Notes.

In the ITS phylogeny, *M. aff. moniliforme* forms a monophyletic group with *M. moniliforme* and *M. cf. moniliforme* with 97% bootstrap support (Fig. 2). Material of this specimen is too limited for more extensive description and analysis, but its sequence divergence from other specimens suggests it might represent a new, possibly cryptic species.

#### Metacapnodium novae-zelandiae

Taxon classificationFungiCapnodialesMetacapnodiaceae

(S. Hughes) Aliabadi & Berbee, 2025 comb. nov.

F2B56356-1FB9-5E21-8E06-594997CE4EB3

Index Fungorum: IF903439

Fig. 12

##### Basionym.

≡ *Capnocybe novae-zelandiae* S. Hughes, N.Z. Jl Bot. 4: 345 (1966).

##### Specimen examined.

New Zealand • South Island, Canterbury District, Woolshed Hill, Hawdon Valley, on bark of *Nothofagus fusca*, 16 May 1963, S.J. Hughes (*Capnocybe novae-zelandiae*DAOM 97302a isotype).

##### Description.

Subicula dark brown to black, superficial, spongy. Mycelia of doliiform and moniliform, brown to dark brown hyphae, smooth throughout but roughened at the distal parts. Hyphae anastomosing, branched, straight and curved, tapering towards their distal tips. Cells subglobose to doliiform, to 32 µm wide, narrowing to 6 µm at hyphal tips.

**Sexual form** unknown.

**Figure 12.**
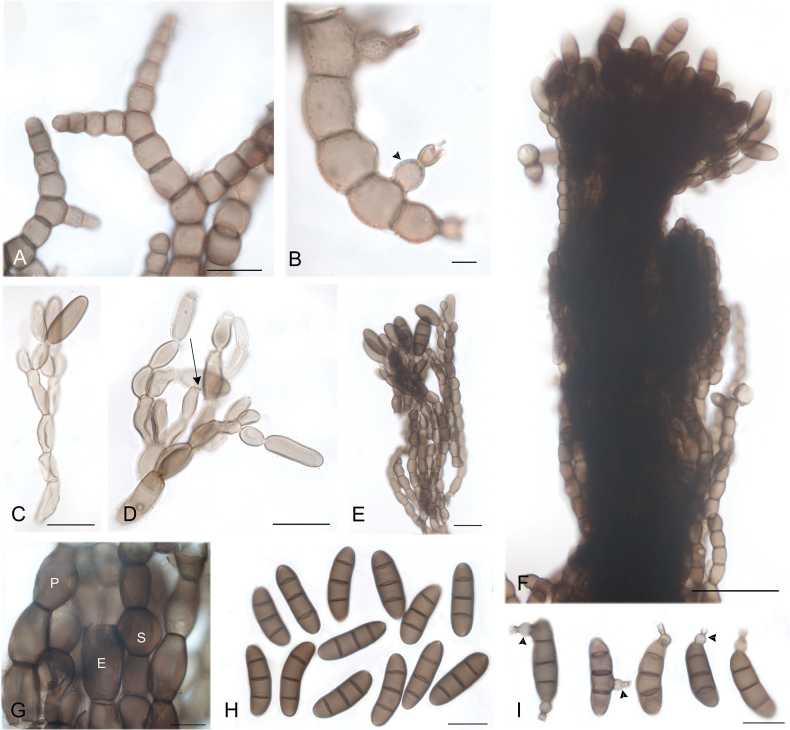
*Metacapnodium novae–zelandiae* (DAOM 97302a). **A**. Moniliform, branched hyphae; **B**. Phialides (arrowhead); **C–I**. Capnocybe form; **C–E**. Synnematous hyphae from the head of a synnema, showing penicillate branching of elliptical cells, constricted at septa; **D**. Denticles (arrow) where conidia seceded; **F**. Overview of synnema; **G**. Shapes of cells of basal internal synnematous hyphae (E = ellipsoid, P = pyriform, S = subglobose); **H**. Conidia; **I**. Conidia bearing phialides (arrowheads). Scale bars: 20 μm (**A, C–E, G–I**); 5 μm (**B**); 50 μm (**F**).

**Asexual forms**. Capnocybe – Synnemata sparsely distributed throughout subicula, erect, cylindrical, up to 2.5 mm high, with a slimy head (Fig. 12F). Stipes 100–150 µm wide, of dense, compact, fasciculate hyphae of cylindrical to ellipsoidal cells that taper to 5.5–6 µm towards the hyphal tips. Internal hyphal cells at synnema base globose, ellipsoidal to pyriform, to 40 µm wide. Heads of synnemata, up to 350 µm wide, their hyphae with penicillate branching. Terminal cells almost ellipsoid, 10–16 long and 5.5–7 wide, bearing denticulate scars where successive conidia budded out, then seceded. Conidia 3–septate (although Hughes (1966) reported rare 2-septate conidia) ellipsoid, rounded at both ends, straight to somewhat curved, dark brown, thick-walled, paler at their apices, (31)–35.8–(39) +/– 2.7 × (9)–11–(13) +/– 1.0 µm, N = 20, Q = 3.2.

Capnophialophora – Phialides from tips or sides of hyphae or from conidia, with light brown to brown, roughened, subspherical venters 5–6 µm wide; each bearing a funnel-shaped collarette up to 5 µm long with a deep constriction < 2 µm wide at its base, expanding to 3–4 µm at its opening. Phialidic conidia not observed.

##### Hosts and distribution.

Collected from the bark of *Nothofagus fusca* in New Zealand, from a mixture with other sooty mold species, probably on insect honeydew (Hughes 1966).

##### Notes.

Specimen DAOM 97302a appears to be a split of the holotype specimen, PDD 24945. We could not obtain a sequence from DAOM 97302a but morphologically, its capnocybe form is similar to *M. ericophilum* and *M. spongiosum*. Hughes (1966) distinguishes *M. novae-zelandiae* by the funnel-shaped collarettes of its phialides and by elliptical cells, strongly constricted at the septa in the penicillate hyphae of its synnema, compared with *M. spongiosum* with its nearly cylindrical collarettes in phialides, and cylindrical cells, only slightly constricted at the septa, in the penicillate hyphae of the synnema. These same characters distinguish *M. novae-zelandiae* from *M. ericophilum*.

Consistent with Hughes’ (1966) study, two different sexual forms can be found in the subiculum of the DAOM 97302a but it is unclear which, if either, is the sexual form of *M. novae-zelandiae*. The specimen also bears many gemma-like, tight aggregations of highly branched hyphae, much as described by Hughes et al. (2012) for *M. gemmiferum*. One of the sexual forms in this collection resembles *M. gemmiferum*; however, it is not fully consistent with that species because longitudinal septations are rare in its ascospores.

#### Metacapnodium pacificus

Taxon classificationFungiCapnodialesMetacapnodiaceae

(S. Hughes) Aliabadi & Berbee, 2025 comb. nov.

BF8F837B-9EC5-5C19-8EFA-62EB2C6D15CA

Index Fungorum: IF904053

Fig. 13

##### Basionym.

≡ *Capnobotrys pacificus* S. Hughes [as ‘*pacifica*’], New Zealand J. Bot. 19(2): 218 (1981).

##### Specimen examined.

New Zealand • Wellington, Mt. Ruapehu, On leaves of *Coprosma tenuifolia*, Cheeseman, 11 Oct 1966, J.M. Dingley, *Capnobotrys pacificus*DAOM 117157c a (ex type).

##### Description.

Subicula not observed, as the specimen was available as a microscopic slide. Mycelium of moniliform hyphae, olive-brown, finely to coarsely verrucose throughout, verrucosity most prominent at the hyphal tips. Hyphae straight to curved, with lateral branching, tapering toward the ends, cells subglobose, equal to or broader than long, to 18 μm wide, narrowing to 5 μm wide at the hyphal tips.

**Sexual form** unknown.

**Figure 13.**
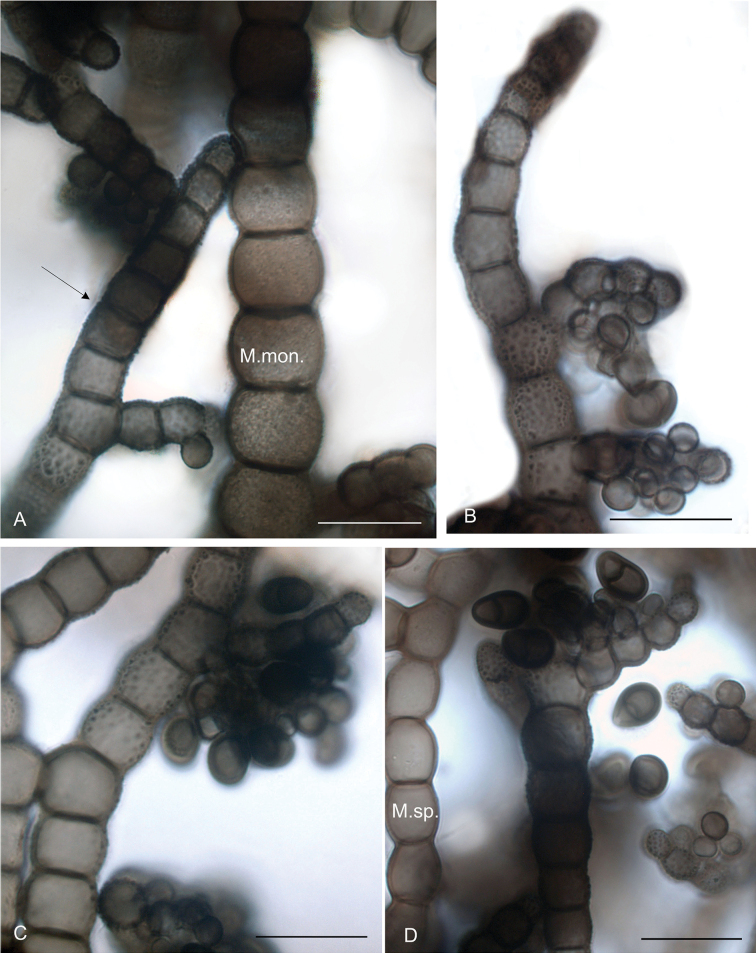
*Metacapnodium pacificus* (DAOM 117157c). **A**. Intermingled in a subiculum, moniliform hypha of *M. pacificus* (at left, arrow); hyphae of *M. moniliforme* (at right, M.mon.) with larger, rounder cells; **B–D**. Capnobotrys form, clusters of conidiogenous cells; **D**. Mature, septate conidia near clusters of conidiogenous cells; at the far left (M.sp.), a light brown hypha, possibly of a different *Metacapnodium* species. Scale bars: 20 μm.

**Asexual forms**. Capnobotrys – Differentiated conidiophores not observed. Conidiogenous cells in botryose clusters, often on the concave underside of short 2–5 septate, lateral branches. Conidia olivaceous brown, initially globose, then ovoid to pyriform, approaching triangular due to narrowing from the proximal cell to the tip of the distal cell, sometimes inequilateral, bearing one, supramedial septum, proximal cells are broader than distal cells (Fig. 13D). Conidia measured 10–12 × 7–8 µm, N = 14, Q = 1.4.

Capnophialophora – No capnophialophora observed in specimen.

##### Hosts and distribution.

On leaves of woody dicots, collected in New Zealand, Chile, Hawaii (Hughes 1981).

##### Notes.

Hughes (1981): figs 19N-R illustrate capnophialophora in DAOM 105684b and DAOM 106093d. Hughes (1981) reports that phialides, when present, have constrictions at the base of the collarette, distinguishing this species from the otherwise similar *M. atro-olivaceus*. Darker color, coarse warts on hyphal surfaces, and narrower hyphae distinguish *M. pacificus* from *M. moniliforme* (Hughes, 1981) and several other species with capnobotrys forms.

#### Metacapnodium spongiosum

Taxon classificationFungiCapnodialesMetacapnodiaceae

S. Hughes & Sivan.

2D0C91E2-3D0F-50FB-9786-884EA1D68D24

Fig. 14

##### Specimens examined.

Usa • Oregon, Douglas County, Bureau of Land Management, North Myrtle Creek Research Natural Area, on stem of *Calocedrus decurrens* (Torr.) Florin, 27 Oct. 1997, Trappe, J., OSC 61673; • Oregon, Douglas County, Roseburg, from yard of Greg Sundquist; Plant Clinic No. 09-47, on *Calocedrus decurrens* (Torr.) Florin, 20 Jan. 2009, Raini Rippy, OSC 135428.

**Figure 14.**
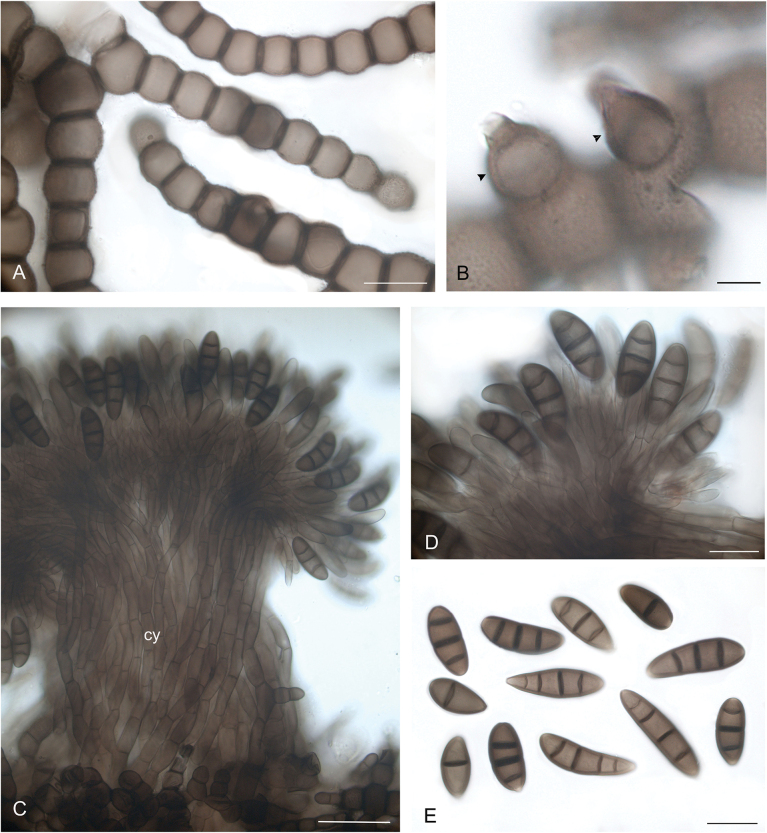
*Metacapnodium spongiosum* (OSC61673). **A**. Moniliform hyphae; **B**. Phialides (arrowheads); **C–E**. Capnocybe form, synnema; **C**. Overview, cy = cylindrical internal hyphae; **D**. Tips of synnematous hyphae with conidia; **E**. Conidia. Scale bars: 20 µm (**A, D, E**); 5 µm (**B**); 50 µm (**C**).

##### GenBank accession number.

Collection OSC 135428, ITS, OR532927.

##### Description.

Subicula black, superficial, and thick. Hoerl (1939) describes subicula as 13–19 mm thick, and illustrates one extending more than 12 cm over a branch.

**Sexual form**. Not observed.

**Asexual forms**. Capnocybe – Synnemata sparse throughout the subicula, hyphae loosely compacted at their bases. The stipe is composed of fasciculate hyphae that are closely compacted with somewhat ellipsoidal to cylindrical cells, branching in a penicillate manner towards the apex. Conidia ellipsoid with a conspicuous clear area at their tips, 1–5, mostly 3–septate, dark brown. Conidia from OSC 135428, (30)–35.8–(38) +/– 3.0 × (9)–10.85–(12) +/– 1.3 µm, N = 20 (3–septate); OSC 61673 (25)–35–(39) +/– 3.9 × (9)–12–(14) +/– 1.0 µm, N = 18 (3–septate) 39–45 × 10.5–12 µm (4–septate).

Capnophialophora – Phialides similar to *M. ericophilum*, collarettes subcylindrical rather than flaring.

##### Host and distribution.

USA, California and Oregon, on branches of conifers *Calocedrus decurrens*, *Chamaecyparis lawsoniana*, *Hesperocyparis sargentii* (Hughes 1981).

##### Notes.

Hughes (1966) describes conidia of *M. spongiosum* as having from 1–5 septa, while conidia of *M. novae-zelandiae* are three, rarely two septate. In *M. novae-zelandiae*, the collarettes of the phialides are funnel-shaped and their bases narrow to 1.8–2.0 µm, not subcylindrical as in *M. spongiosum* (Hughes 1966). Within the phylogenetic ITS tree, *Metacapnodium spongiosum* OSC 135428 appears as the sister to *M. ericophilum*, both of which have a capnocybe form. Hughes’ (1966) descriptions of *M. spongiosum* are based in part on specimens from the Mediterranean region. The Mediterranean specimens may be better identified as *M. ericophilum*, a species that Hughes considered a synonym of *M. spongiosum*.

#### Metacapnodium stanhughesii

Taxon classificationFungiCapnodialesMetacapnodiaceae

Berbee & Aliabadi sp. nov.

B0648159-CDBD-5209-9993-A133B4EFB7A5

Index Fungorum: IF902895

Fig. 15

##### Typification.

Canada • British Columbia Province, Vancouver, The University of British Columbia, 2075–2099 Main Mall, 49.26504°N, 123.25290°W, on bark of *Taxus* sp., 2 July 2021, F. Aliabadi & L. Le Renard (**holotype**: UBC F35817).

##### Etymology.

Named in honor of mycologist *Stanley J. Hughes*.

##### GenBank accession numbers.

ITS, OR532926; LSU, PP140681; *ef1-α*, OR820949.

##### Description.

Subicula dark brown to black, velutinous, thin, up to 2–4 mm thick, covering the bark of a trunk of *Taxus* sp. and intermingled with other sooty molds. Mycelium of moniliform hyphae, hyphae brown to dark brown, constricted at septa, surfaces finely verrucose throughout, cell wall thickness of 0.5–1 μm. Hyphae curved, straight, occasionally anastomosing, narrowing towards their tips. Cells usually broader than long, subglobose to doliiform, 13–21 × 10–16 μm, narrowing to 5–7 μm at hyphal tips.

**Sexual form** unknown.

**Asexual forms**. Capnobotrys – Differentiated conidiophores not observed. Conidiogenous cells in botryose clusters laterally or terminally on hyphal tips or at ends of short, 1–2 celled lateral branches, spherical, subspherical, ellipsoid, 3–10 × 3–9 μm, light brown to dark brown, bearing denticulate scars where successive conidia budded out, then seceded. Conidia brown to dark brown, smooth walled, initially subglobose, then ellipsoid or ovoid, occasionally cornute or allantoid, with a single median or supramedial septum, not or only slightly constricted at septa. Conidia (10)–12.7–(15) +/– 1.3 × (6)–7.7–(9) +/– 0.9 µm, N = 30, Q = 1.7. Proximal cells longer and wider than distal cells; proximal cell 7.0 +/- 0.9 × 7.7 +/- 0.8 µm; distal cell 5.7 +/- 0.9 × 7.0 +/- 0.7 µm. Proximal cell: distal cell length ratio, 1.3, width ratio 1.2. Proximal cells sometimes with inconspicuous oblique scar resulting from secession from diagonal attachment to a conidiogenous cell; apex of distal cells shows wall thinning, light in color, which sometimes protrudes slightly.

Capnophialophora – Phialides scanty, occasionally at hyphal tips, with a verruculose, brown, subspherical venter, 4–5 × 5 μm, bearing a funnel-shaped, pale brown collarette 2–3 μm long, with a narrow constriction at its base, 2.5–3 μm wide at its opening. No phialidic conidia observed.

Capnosporium – Branched, erect hyphae bear individual conidia at or near their tip cells. Capnosporium conidia ellipsoid, obclavate, mainly straight, occasionally curved, 2–6 septate, sessile, 19–21 × 8–10 μm (2–septate), 27–34 × 8–12 μm (3–septate), 33–42(–47) × 11–12 μm (4–septate) μm.

##### Host and distribution.

Known only from a single collection on *Taxus* sp. in shaded, landscaped area next to Chemistry Building on University of British Columbia campus. The subiculum, first noticed by Ludovic Le Renard, changed little over five years.

##### Notes.

**Figure 15.**
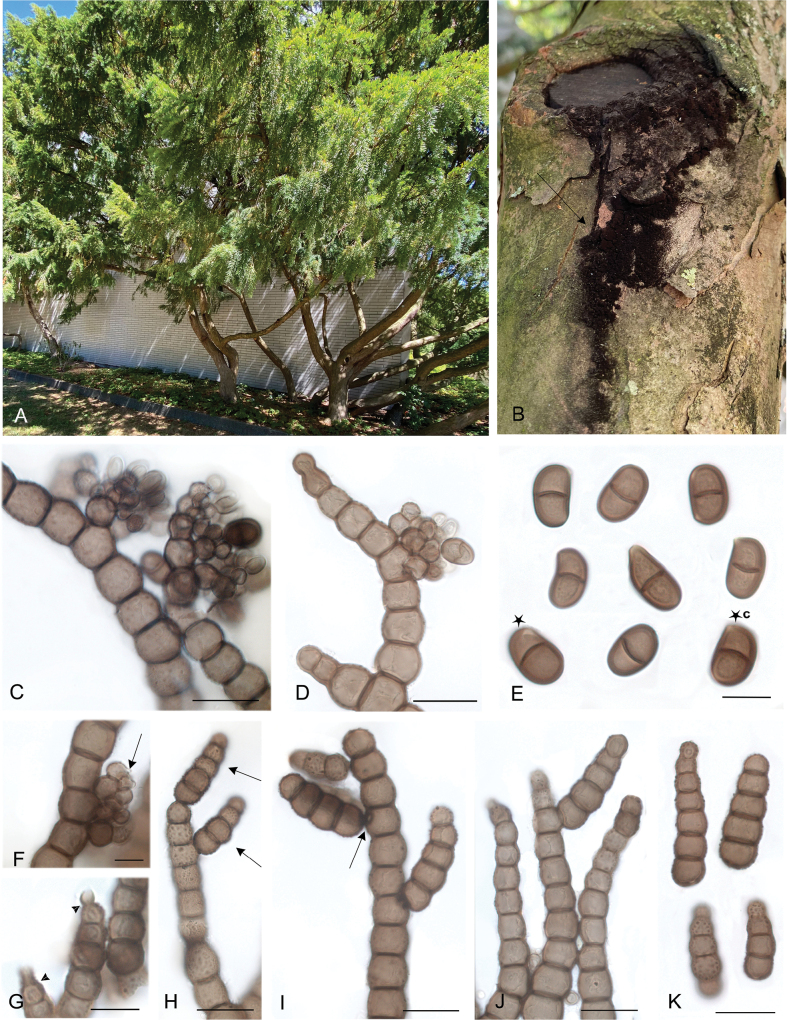
*Metacapnodium stanhughesii* (UBC F35817, holotype). **A**. *Taxus* sp., host of *M. stanhughesii*; **B**. Trunk of *Taxus* sp. colonized by *M. stanhughesii* (arrow); **C–F**. Capnobotrys form; **C, D, F**. Moniliform hyphae with conidiogenous cell clusters; **E**. Conidia, asterisks designate distal wall thinning; c designates one of the cornute conidia; **F**. Denticles (arrow) on conidiogenous cell; **G**. Phialides (arrowheads); **H–K**. Capnosporium form; **H**. Conidia (arrows); **I**. Pores (arrow); **K**. Capnosporium conidia showing variation in septation. Scale bars: 20 μm (**C, D, H–K**);10 μm (**E, G**); 5 μm (**F**).

Among the described *Metacapnodium* species, *M. moniliforme* is similar to *M. stanhughesii* in morphology. Phylogenetically, *M. stanhughesii* cannot be included in *M. moniliforme* because the ITS tree and the concatenated region tree show *M. stanhughesii* as branching outside of a clade consisting of *M. neesii* and *M. moniliforme*. *Metacapnodium guava* is also similar to *M. stanhughesii* in that it shares capnophialophora, capnobotrys, and capnosporium forms (Hughes, 1981). While the dimensions of conidia in *M. stanhughesii* fall between those of *M. moniliforme* and *M. guava*, *M. stanhughesii* (Fig. 15E) and *M. moniliforme* (Fig. 10I) exhibit cornute conidia with a slight point at one end, which were not seen in *M. guava* (Hughes, 1981). Cornute and allantoid conidial morphology arise in *Capnobotrys lechlerianus* (Hughes and Seifert 2012), but the hyphal walls of *Capnobotrys lechlerianus* are smooth rather than verrucose as in *M. stanhughesii* and *M. moniliforme*. Hughes and Seifert (2012) used the absence of capnophialophora and capnosporium forms, and the smaller dimensions of conidia to distinguish *Capnobotrys lechlerianus* from the capnobotrys form of *M. moniliforme* (Hughes and Seifert 2012).

#### Metacapnodium vancouverensis

Taxon classificationFungiCapnodialesMetacapnodiaceae

Aliabadi & Berbee sp. nov.

AA2661F2-617F-5D73-8F7F-50481025B015

Index Fungorum: IF902896

Fig. 16

##### Typification.

Canada • British Columbia Province, Vancouver, The University of British Columbia, 6246–6298 Agricultural Road (49.2664°N, 123.2534°W), on leaves of *Pieris japonica*, 22 July 2021, F. Aliabadi (**holotype**: UBC F35816).

##### Etymology.

The epithet named after the city where the species was first found.

##### GenBank accession number.

ITS, OR532925.

##### Description.

**Figure 16.**
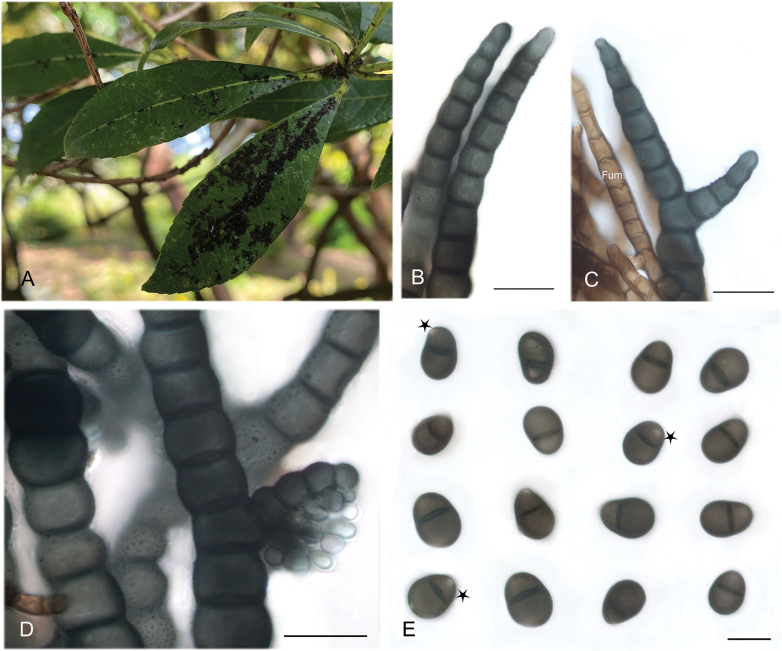
*Metacapnodium vancouverensis* (UBC F35816). **A**. Leaf surfaces of *Pieris japonica* colonized by *Metacapnodium vancouverensis* and other sooty molds; **B**. Tapering hyphae of doliiform cells; **C**. Mixed bluish hyphae of *M. vancouverensis* (to the right) with brown *Fumiglobus pieridicola* (Fum) hyphae to the left; **D, E**. Capnobotrys form; **D**. Cluster of conidiogenous cells; **E**. Conidia (asterisks designate distal wall thinning). Scale bars: 20 μm (**B–D**); 10 μm (**E**).

Subicula black, superficial in 2–3 mm, cushion–shaped patches and tufted mycelia on upper surfaces of leaves of *Pieris japonica*, intermingled with *Fumiglobus pieridicola* and other fungi (Fig. 16A). Mycelia of moniliform hyphae. Hyphae blue green, turning to light brownish green when mature, smooth to finely verrucose throughout, fine warts most abundant at hyphal tips, constrictions at septa of the widest of the mature cells. Hyphae straight to curved, branching, then growing upwards, anastomosing, tapering towards their tips. Upright hyphae develop lateral branches of 60–100 μm. Mature cells usually broader than long, subglobose to doliiform, (10–)12–23(–27) μm wide and 10–18(–21) μm long, narrowing to 6–8 μm at hyphal tips (Fig. 16B, C).

**Sexual form** unknown.

**Asexual forms**. Capnobotrys – Differentiated conidiophores not observed. Conidiogenous cells uniseriate or in clusters of 20–30 μm, often crowded on concave side of curved, downward pointing, 1–6 septate branches, globose, subglobose to ovoid, pale green to bright blue–green. Conidiogenous cells bud either terminally or laterally from hyphae. Conidia arise on new growing points that develop in succession on a conidiogenous cell. Conidia pyriform, allantoid, approaching triangular due to narrowing from the proximal cell to the tip of the distal cell, bearing a single supramedial septum, (9)–11.5–(4.5) +/– 1.3 × (7)–8.4–(10) +/– 0.9 μm, N = 30, Q = 1.4 (Fig. 16E). Proximal cell 6.9 +/- 0.9 × 8.4 +/- 0.9 μm; distal cell 4.7 +/- 0.9 × 6.4 +/- 0.89 μm, ratio of proximal to distal cell length 1.5; ratio of proximal to distal cell width 1.3. Proximal cells sometimes with inconspicuous, oblique scar from secession from basal or diagonal attachment to its conidiogenous cell. Distal cell with an apical patch of thinner, paler wall that sometimes protrudes slightly.

Capnophialophora – Unknown.

##### Host and distribution.

Known only from *Pieris japonica* leaves, from landscape planting at the University of British Columbia, Vancouver BC Canada. Under a dissecting microscope, small subicula could be distinguished by their color from intermingled *Fumiglobus pieridicola*, a sooty mold in *Capnodiales*.

##### Notes.

In ITS trees, *M. vancouverensis* does not group closely with other species. *M. vancouverensis* hyphae share unusually dark, brownish pigmentation with *M. pacificus* and *M. atro-olivaceus* but appears blue–green to olivaceous rather than dark olivaceous brown as in the other two species. Like *M. pacificus*, *M. vancouverensis* regularly produces conidia on the concave side of short, downward pointing lateral branches. Conidia in both species have an outline approaching a triangle, due to their relatively large width relative to length (low Q) and the tapering of the distal cell towards its tip. However, *M. pacificus* has narrower hyphae with denser verrucae. The specimens of *M. atro-olivaceus* and *M. pacificus* were from the 1960’s and composed of dense mixtures of *Metacapnodiaceae* species, and it was not feasible to obtain sequences from them.

#### Metacapnodium

Taxon classificationFungiCapnodialesMetacapnodiaceae

sp.

D9A8BC75-7776-50FD-B586-9E2823336693

Fig. 17

##### Specimen examined.

USA • New Hampshire, Coos County, Mount Washington quad. (NAD 27), Mount Washington Auto Road. 44.28323°N, 071.26138°W, on *Betula papyrifera*, 25 Sept 2003, J.L. Crane, DAOM 239041; Norway • Ålesund, Skodje: Heggebakklia, on *Taxus baccata* L., 11 Dec. 2002, D. Holtan, O F201613.

**Figure 17.**
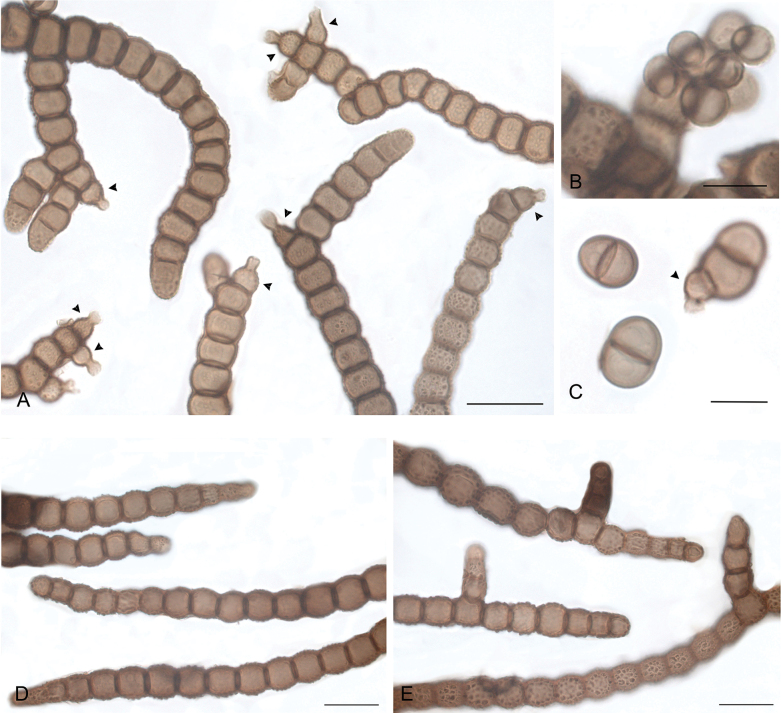
*Metacapnodium* sp. (DAOM239041). **A**. Moniliform hyphae bearing phialides (arrowheads show phialides); **B**. Conidiogenous cells cluster (capnobotrys form); **C**. Conidia (arrowhead shows phialide). *Metacapnodium* sp. (O F201613). **D**. Moniliform hyphae; **E**. Hyphae with lateral branch. Scale bars: 20 μm (**A, D, E**); 10 μm (**B, C**).

##### GenBank accession numbers.

Collection DAOM 239041 (ITS, OR532933); Collection O F201613 (ITS, OR532932).

##### Description.

Subiculum black, about 3.5 cm wide, covering a piece of birch bark (DAOM 239041); covering two fragments, each ~2–3 cm long of bark of *Taxus baccata*, (O F201613). Mycelium of moniliform hyphae with anisotomous branching and slight constrictions at septa. Hyphae taper towards their tips and are brown to dark brown when mature, verrucose throughout, cells as broad as or broader than long, 9–17(–18) µm wide, tip cells are 4–7 µm wide, smooth to finely verrucose. Hyphae are erect or curved; branches grow upwards. Branches with 4–5 septa measured from 35–40 in length; branches with 12 or more septa measured up to 110 µm in length.

**Sexual form** unknown.

**Asexual forms**. Capnobotrys – Present only in DAOM 239041. Conidiogenous cells in whorls or clusters at the lateral hyphae, globose, subglobose, and ellipsoid, 5.5–7 × 4.5–7 µm, brown to dark brown. Only four conidia found, these ellipsoid, brown, smooth walled, 1-septate, 9–13 × 6–9 (–10) µm.

Capnophialophora – Present only in DAOM 239041. Phialides at the sides and tips of hyphae and conidia, light brown to brown with spherical to hemispherical venters 4–5(–6) µm wide, bearing a collarette up to 2.5–3 µm long and 3 µm wide that arises from a deep constriction.

##### Host and distribution.

Collected from bark from disparate hosts and localities, from *Betula* from eastern USA and from *Taxus* from Norway.

##### Notes.

ITS sequences of *Metacapnodium* sp. (DAOM 239041) and *Metacapnodium* sp. (O F201613) are 98.9% identical (Suppl. material 3: fig. S1). While their hyphal sizes and surface ornamentation are similar, their collection localities are from different continents and their plant hosts are distantly related. *Metacapnodium* sp. DAOM 239041 and O F201613 might represent the same or different new species, but in the absence of more distinctive morphological and molecular characters, identification or species delimitation will have to wait for additional collections.

#### Ophiocapnocoma phloiophilia

Taxon classificationFungiCapnodialesMetacapnodiaceae

(E.E. Fisher) S. Hughes

7BCF3CE4-E29E-53E6-8CC0-921469C6E648

Fig. 18

##### Specimen examined.

Usa • Oregon, Douglas Co., Creek Research Natural Area, on *Calocedrus decurrens* stem, 27 Oct 1997, J. Trappe, OSC 61673.

**Figure 18.**
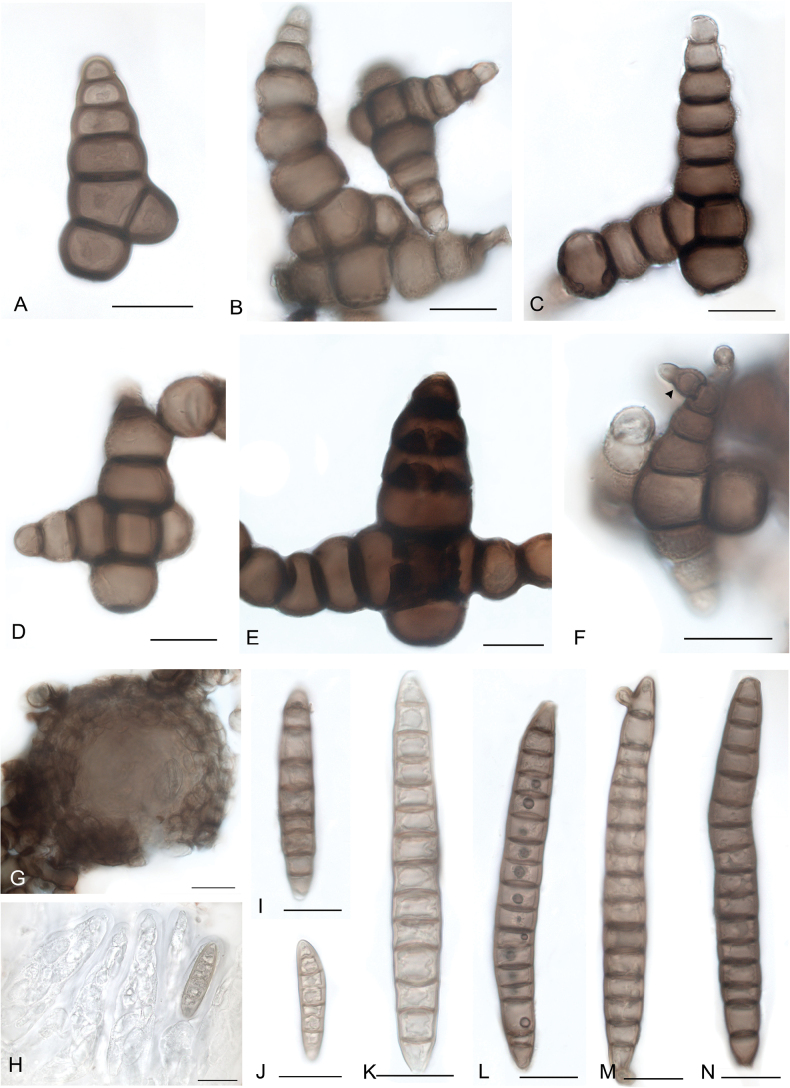
*Ophiocapnocoma phloiophilia* (OSC 61673). **A–E**. Young and mature porospores (hormiokrypsis); **F**. Porospore bearing phialides (arrowhead); **G**. Perithecium; **H**. Asci; **I–N**. Ascospores. Scale bars: 20 µm.

##### Description.

Subicula dark brown to black, forming spongy cushions, up to 10 mm thick and up to 75 mm in diameter (Hughes 1967). Mycelium of moniliform, anastomosing, branched hyphae with deep constrictions at septa. Hyphae tapering towards their tips; brown to dark brown when mature, finely verrucose (not illustrated).

**Sexual form**. Perithecia superficial, scattered, subglobose and dark brown to black. Asci obclavate, 100–180 × 20–40 µm. Ascospores are ellipsoidal to fusiform, curved or straight, yellowish brown to brown, to 22–septate, according to Hughes (1967), up to 15 septa found in OSC 61673.

**Asexual forms**. Hormiokrypsis – Conidiophores thick, robust, darker than somatic hyphae. Conidia are porospores, arising from a pore in the thickened distal cell wall at the tip of a conidiophore (not illustrated). Hormiokrypsis porospores are multiseptate and often consist of a main axis with one or two arms. They display a wide range of sizes and shapes. Developing porospores elongate from subglobose to ellipsoid, becoming obclavate while also developing transverse septa. Mature porospores consist of an obclavate, straight main axis comprising four to nine cells, and two, septate arms that originate from the supra–basal cell of the main axis and taper towards their tips. Basal cells somewhat hemispherical, up to 25 µm wide, main axis length up to 100 µm. Lateral arms perpendicular to, and generally shorter and narrower than, the main axis.

Capnophialophora – Phialides form on hyphae, on hormiokrypsis porospores and on ascospores.

##### Host and distribution.

Hughes (1967) reports this species from New Zealand, Australia, and Hawaii and western USA, growing on stems of various woody plants, often in mixtures of sooty mold species.

##### Notes.

*Ophiocapnocoma phloiophilia* can be distinguished by its hormiokrypsis porospores, which are unique to this species. We were unsuccessful in separating *O. phloiophilia* for sequencing from among other, intermingled fungi present in specimen OSC 61673, which included *M. spongiosum*, described above. This leaves the phylogenetic relationship between *Metacapnodium* and *Ophiocapnocoma* unresolved.

## Discussion

### Phylogenetic position of *Metacapnodiaceae* in *Ascomycota*

Analysis of concatenated LSU, SSU, *rpb2* and *ef1-α* genes shows that *Metacapnodiaceae* belongs in class *Eurotiomycetes*, subclass *Chaetothyriomycetidae*, consistent with phylogenies from ITS (Hawksworth and Boluda 2020) and LSU data (Sugiyama et al. 2020; Marasinghe et al. 2023). While *Metacapnodiaceae* appeared as the sister group to *Pleostigmataceae* in Fig. 3 from concatenated data, and although the ITS phylogeny in Fig. 2 was therefore rooted to show *Metacapnodiaceae* and *Pleostigmataceae* as sister groups, support for this relationship was not strong and it was not mirrored in individual gene trees (Suppl. material 3: figs S2–S5). Because of the lack of certainty about relationships among families, we chose not to propose a new order for *Metacapnodiaceae*.

More extensive sampling from taxa and sequences from multiple loci from *Chaetothyriomycetidae* would likely improve resolution. This is not yet accomplished because the organisms are intractable and any potential economic value they have has yet to be monetized. In nature, the epiphyllous, lichenized, lichenicolous, and rock-dwelling taxa that predominate in *Chaetothyriomycetidae*, like *Metacapnodium* species, grow in species mixtures, making it difficult to separate them for sequence analysis. Few cultures are available; like *Metacapnodium* species, other members of the *Chaetothyriomycetidae* grow slowly if at all in pure culture. Muggia et al. (2021) successfully cultured more than 29 isolates of alpine, endolithic *Pleostigmataceae* species. The isolates only grew to 1–3 mm in three to five months but that was enough for sequencing of ITS and/or LSU regions, supporting erection of new family *Pleostigmataceae*. Isolates of *Sorocybe* spp. (also *Pleostigmataceae*, Figs 2, 3) grow more rapidly although “even after 3 mo, the colonies are rarely more than 2 cm diam” (Seifert et al. 2007), while *Neosorocybe pini* reaches 2 cm in diameter after two weeks. *Sorocybe* and *Neosorocybe* species may serve as the best available models for further study of this difficult clade.

To grow, *Metacapnodium* species on plant surfaces share environmental challenges with *Pleostigmataceae* and *Verrucariales*. Gueidan et al. (2007, 2008) pointed out that although *Verrucariales* and related clades vary, their ancestral state is reconstructed as rock-inhabiting and non-lichenized. Muggia et al. (2021) noted that most rock-associated *Pleostigmataceae* and allied lichen-associated taxa are highly melanized, perhaps in adaptation to surface exposure to variation in temperature and humidity, and to high UV radiation. *Metacapnodium* spp. are similarly highly melanized, perhaps for the same reason. The lichenicolous *Pleostigmataceae* do not appear to damage their host lichens but perhaps gain nutrients instead as commensals, absorbing lichen secretions (Muggia et al. 2021) perhaps as *Metacapnodiaceae* absorb secretions from honeydew on plants.

### Fossil interpretation

We reinterpret fossils from Baltic and Bitterfeld amber described as *Metacapnodium succinum* (Rikkinen et al. 2003) to provide a minimum age of 35 Ma for the divergence of stem *Metacapnodiaceae* from *Pleostigmataceae* assuming that phylogenies Figs 2, 3 correctly reconstruct relationships. More conservatively, the fossils provide a minimum age for the older, better supported divergence between the *Metacapnodium* clade and *Chaetothyriales*. Reinterpretation is necessary because *Metacapnodium* was classified with morphologically similar sooty molds in *Capnodiales*, *Dothideomycetes* until Hawksworth and Boluda (2020) and Sugiyama et al. (2020) showed that it belongs in *Chaetothyriomycetidae*. Lacking accurate phylogenetic information, fossils of *Metacapnodium succinum* in amber were reasonably but incorrectly used to date *Capnodiales* (Beimforde et al. 2014; Samarakoon et al. 2019) rather than the split between *Metacapnodium* and its sister taxon.

Further, the minimum age of the *Metacapnodium* crown group had been presumed to be Cretaceous, 100 Ma (Beimforde et al. 2014; Samarakoon et al. 2019), based on fossils of mycelium fragments, which, on close examination lack diagnostic *Metacapnodium* characters.

Mycelium morphology in these Cretaceous fossils (Schmidt et al. 2014, plates II, III) suggests a mixture of species with different types of moniliform hyphae; none of which display consistent tapering of hyphae towards their tips. They may represent any of a number of unrelated lineages of *Ascomycota* that share moniliform hyphae of cells that are as long or slightly longer than wide. Examples of extant taxa with moniliform morphology include the mycelium of rock-inhabiting *Pleostigmataceae* (Muggia et al. 2021), the branched chains of conidia in *Torula mackenziei*, *Pleosporales* (Li et al. 2017), hyphae of saxicolous lichens like *Dermatocarpon tomentulosum*, *Verrucariales* (Anja 2006) and *Reichlingia leopoldii*, *Arthoniales* (Aptroot and J. M. Sipman 2001), and hyphae on plant surfaces from the sooty mold *Fumiglobus pieridicola*, *Capnodiales* (Bose et al. 2014).

A more convincing minimum age for the *Metacapnodium* stem lineage, 35 Ma, comes from a fossil from Baltic amber, Eocene in age, 55–35 Ma (Rikkinen et al. 2003, specimen V. Arnold, no. 1371, fig. 1). This fossil, beautifully re-photographed by Schmidt et al. 2014 (plate VI, figs 1–4) shares *Metacapnodium* characters common to the monophyletic clade analyzed here: hyphal cells broader than wide, cells up to 13 µm broad (unusually wide), and hyphae of regularly shaped, bead-like, moniliform cells that narrow gradually towards hyphal tips. These characters are unusual among other ascomycete groups and absent in other sooty molds.

A second fossil, from Bitterfeld amber, late Oligocene to early Miocene in age, 24–22 Ma, in Rikkinen et al. 2003 (specimen GZG.BST.27291, figs 2, 3) and in Schmidt et al. 2014 (plate VI, fig. 5) bears phialides consistent with identification of the capnophialophora form of a *Metacapnodium* species. The phialides arise at the sides or tip of hyphal cells that are moniliform, ~15 µm broad, and broader than long. The phialides appear to be about 10 µm long, brown in color with a hemispherical venter ~7.3 µm long and a prominent collarette ~2.3 µm long, which narrows slightly at its opening.

Because the characters that distinguish *Metacapnodium* are widely shared by species in the genus, they most likely evolved among ancestors in the stem taxon that preceded the first divergence of crown *Metacapnodium*. This implies that fossils with *Metacapnodium* morphology provide a minimum age for the divergence of *Metacapnodium* from its closest sister group and not to the common ancestor of the crown group (Pirie and Doyle 2012). Critical re-evaluation of calibrating fossils is a step toward ensuring accurate minimum age estimates. This will likely lead to overall underestimates of actual ages of clades but may stimulate the search for older, equally well-preserved fossils.

It is yet unclear when and how *Metacapnodiaceae* evolved and spread across the globe, and the existence of genetically closely related species spread tens of thousands of kilometers apart adds to the mystery of their evolution and current distribution. Gymnosperms in the genus *Sciadopitys* are interpreted as the producers of the resin of Bitterfeld amber (Wolfe et al. 2016), and they have an extensive fossil record in the North Hemisphere (Hofmann et al. 2021). Nowadays restricted to a single species in Asia, *S. verticillata*, the study of *Sciadopitys*-bearing fossil deposits has the potential to track the spread of *Metacapnodiaceae* in northern latitudes.

### Relationships within *Metacapnodiaceae*

In this study, we expand the number of *Metacapnodium* species represented by at least fragments of barcode sequences from two to 10 species, add a second living culture of a *Metacapnodium* species to a national culture collection, provide morphological and sequence comparisons of specimens identified by experts, notably S. Hughes, and a preliminary key to species. These resources will, we hope, scaffold further systematic studies of a fascinating and challenging genus.

*Metacapnodium* appears monophyletic in our ITS tree. It encompasses the type species *M. juniperi*, two pleomorphic species with sexual and capnocybe asexual forms, three pleomorphic species with sexual and capnobotrys asexual forms, and four species (*M. australis*, *M. stanhughesii*, *M. vancouverensis*, and *M.* sp. DAOM 239041) known only by their capnobotrys asexual forms. This evidence for monophyly supports the wisdom of conserving *Metacapnodium* against other names for sexual and asexual forms (May 2024).

Ideally, molecular systematic analysis and species delimitation would be based on comparison of multiple specimens of the same species and sequences for multiple loci from each specimen. Species pairs such as *M. ericophilum* and *M. spongiosum* or *M. moniliforme* and *M. stanhughesii* are difficult to separate without ITS sequence data, and we could only presume that specimens differing by 2% or more in their ITS sequences were likely to be different species. Recent, rich collections of specimens in this genus are few and far between. Limiting our opportunities for critical comparisons within and between species, we encountered high failure rates when amplifying or sequencing DNA from minute, carefully selected patches of mycelium that were more than 20 years old.

To address many unknowns in biology of fungi such as *Metacapnodiaceae*, taxonomy and delimitation of fungal species is important. New Zealand would be a good choice for further systematic studies aimed at species delimitation. The known diversity of *Metacapnodiaceae* is much higher in New Zealand than in other parts of the world, due in part to many years of systematic studies by S. Hughes, and prior work there will facilitate additional research. We look forward to future systematic studies involving the collection and culturing of fresh specimens, then sequencing of ITS regions for initial barcoding, and phylogenomic analysis, leading to broader insight into species diversity, host preferences, ecology and biogeography.

## Supplementary Material

XML Treatment for Metacapnodiaceae

XML Treatment for Metacapnodium adamantinum

XML Treatment for Metacapnodium atro-olivaceus

XML Treatment for Metacapnodium australis

XML Treatment for Metacapnodium dingleyae

XML Treatment for Metacapnodium ericophilum

XML Treatment for Metacapnodium juniperi

XML Treatment for Metacapnodium moniliforme

XML Treatment for Metacapnodium cf. moniliforme

XML Treatment for Metacapnodium aff. moniliforme

XML Treatment for Metacapnodium novae-zelandiae

XML Treatment for Metacapnodium pacificus

XML Treatment for Metacapnodium spongiosum

XML Treatment for Metacapnodium stanhughesii

XML Treatment for Metacapnodium vancouverensis

XML Treatment for Metacapnodium

XML Treatment for Ophiocapnocoma phloiophilia

## Additional information

### Use of AI

No use of AI was reported.

### Author ORCIDs

Faezeh Aliabadi https://orcid.org/0009-0003-1855-2735

Ludovic Le Renard https://orcid.org/0000-0002-1147-4653

Mary L. Berbee https://orcid.org/0000-0002-9850-1174

### Data availability

The data underpinning the analysis reported in this paper were deposited in the Dryad Data Repository at https://doi.org/10.5061/dryad.3bk3j9m0v (Aliabadi et al. 2026).

## Supplementary materials

Supplementary material 1 Accession data and references for previously published DNA sequences Data type docx Explanation note Accession information from JGI and NCBI for previously published sequences used in this study. https://binary.pensoft.net/file/1552579This dataset is made available under the Open Database License (http://opendatacommons.org/licenses/odbl/1.0/). The Open Database License (ODbL) is a license agreement intended to allow users to freely share, modify, and use this Dataset while maintaining this same freedom for others, provided that the original source and author(s) are credited. Faezeh Aliabadi, Ludovic Le Renard, Mary L. Berbee

Supplementary material 2 Alignments used in this study Data type txt Explanation note Contains alignments in nexus format (1) for the ITS region used for the phylogeny in Fig. 2, (2) a concatenated alignment of SSU, LSU, *RPB2* and *ef1-α* used for Fig. 3, and individual alignments of the SSU, LSU, *RPB2* and *ef1-α* used for single locus phylogenies in Suppl.material 3: figs S2–S5. https://binary.pensoft.net/file/1552580This dataset is made available under the Open Database License (http://opendatacommons.org/licenses/odbl/1.0/). The Open Database License (ODbL) is a license agreement intended to allow users to freely share, modify, and use this Dataset while maintaining this same freedom for others, provided that the original source and author(s) are credited. Faezeh Aliabadi, Ludovic Le Renard, Mary L. Berbee

Supplementary material 3 Details of phylogenetic analyses Data type pdf Explanation note **table SS2** outlines characteristics of the alignments, methods and parameters applied in the analyses; **fig. S1** shows pairwise distances among ITS sequences; distmat calculated uncorrected distances as 1-(# nucleotide matches/number of shared sites), expressed as a percentage; number of sites is given for each sequence; distances involving sequences from newly described species are colored; **figs S2**–**S5** show single-locus maximum likelihood phylogenies; numbers at nodes represent transfer bootstrap percentages over 50%; **fig. S2** is from SSU data; **fig. S3** is from LSU data; **fig. S4** is from *ef1-α* data; **fig. S5** is from *rpb2* data. https://binary.pensoft.net/file/1552581This dataset is made available under the Open Database License (http://opendatacommons.org/licenses/odbl/1.0/). The Open Database License (ODbL) is a license agreement intended to allow users to freely share, modify, and use this Dataset while maintaining this same freedom for others, provided that the original source and author(s) are credited. Faezeh Aliabadi, Ludovic Le Renard, Mary L. Berbee
