# Supplementary material for: Taxonomy and phylogeny of the epiphytic sooty molds in family Metacapnodiaceae (class Eurotiomycetes, subclass Chaetothyriomycetidae)
Source: MycoKeys. 2026 Mar 4;129:163–212. doi: 10.3897/mycokeys.129.178067 (PMC13291639; doi:10.3897/mycokeys.129.178067)
Supplement: Supplementary material 1 — Accession data and references for previously published DNA sequences [file mycokeys-129-163-s001.docx]

**Table S1**: Accession data and references for previously published sequences

| species | Locus accession numbers and references to published sequences* |  |  |  |  |
| --- | --- | --- | --- | --- | --- |
|  | SSU | ITS | LSU | ef1a | rpb2 |
| *Aaosphaeria arxii* | jgi\|Aaoar1\|Locus38v1rpkm3327.5vi8:1055-1720 EST cluster; CBS 175.79 (1) |  | jgi\|Aaoar1\|Locus34v1rpkm3777.14:1289-2168 EST cluster; CBS 175.79 (1) | jgi\|Aaoar1\|403805 Transcript ID; CBS 175.79 (1) | jgi\|Aaoar1\|388997 Transcript ID; CBS 175.79 (1) |
| *Alternaria alternata* | jgi\|Altalt1\|scaffold_8:2512932-2520743 Assembly EST cluster; MPI-PUGE-AT-0064 (2) |  | MH877754; CBS 102602 (3) |  |  |
| *Alternaria brassicicola* | KC584515; CBS 118699 ATCC 96836 (4) |  | KC584258; CBS 116528 (4) | jgi\|Altbr1\|10405 Transcript ID; ATCC 96836 (5) | jgi\|Altbr1\|3555 Transcript ID; ATCC 96836 (5) |
| *Amauroascus aureus* |  |  | NG_057025 (6) |  |  |
| *Antennularia pulmonalis* |  |  | MH866351 (3) |  |  |
| *Anthracothecium nanum* |  |  | FJ358271 (7) |  |  |
| *Anthracothecium prasinum* |  |  | KT808552 (7) |  |  |
| *Ascocoryne cylichnium* |  |  | JN086709 (8) |  |  |
| *Ascocoryne sarcoides* | FJ176830; AFTOL-ID_1834 |  | MH867123 (3) | jgi\|Ascsa1\|9222 Transcript ID; NRRL 50072 (9) | jgi\|Ascsa1\|6561 Transcript ID; NRRL 50072 (9) |
| *Aspergillus arachidicola* |  |  | jgi\|Aspara19utr\|TRINITY_DN8538 EST cluster; CBS 117612 (10) |  |  |

| *Aspergillus campestris* | | Aspcam1Aspcam1\|Locus23v1rpkm11238.20; Aspcam1\|Locus11v1rpkm20647.41; Aspcam1\|Locus9v1rpkm26357.93 3 EST clusters; IBT 28561 (10) | |  | | OL898485; IBT 28561 | | jgi\|Aspcam1\|269095 Transcript ID; IBT 28561 (10) | jgi\|Aspcam1\|260244 Transcript ID; IBT 28561 (10) |
| --- | --- | --- | --- | --- | --- | --- | --- | --- | --- |
| *Aspergillus candidus* CBS 102.13 | | jgi\|Aspcand1\|TRINITY_DN5152_c1_g1_i1 EST cluster; CBS 102.13 (10) | |  | | OL898495; CBS 102.13 | | jgi\|Aspcand1\|114762 Transcript ID; CBS 102.13 (10) | jgi\|Aspcand1\|106764; CBS 102.13 (10) |
| *Aureobasidium pullulans* var. *pullulans* | | jgi\|Aurpu_var_pul1\|Locus20v1rpkm3514_83 EST clusters; EXF-150; CBS 100280 (11) | |  | | MH878516; CBS 590.75 (3) | | jgi\|Aurpu_var_pul1\|348177 Transcript ID; EXF-150; CBS 100280 (11) | jgi\|Aurpu_var_pul1\|368090 Transcript ID; EXF-150; CBS 100280 (11) |
| *Auxarthron zuffianum* | |  | |  | | MH876219 (3) | |  |  |
| *Botrytis cinerea* | | JGI\|Botci1\|Supercontig_1_189:15082-52320 Assembly; strain B05.10 (12) (13) | |  | | MH877779; CBS 125.58 (3) | | jgi\|Botci1\|13066 Transcript ID; strain B05.10 (12) (13) | jgi\|Botci1\|11688 Transcript ID; strain B05.10 (12) (13) |
| *Capnodium coffeae* | |  | |  | | MH868489 (3) | |  |  |
| *Capnodium gardeniarum* | |  | |  | | MH878118 (3) | |  |  |
| *Capronia fungicola* | |  | |  | | NG_058761 (7) | |  |  |
| *Capronia mansonii* | |  | |  | | NG_068979 (14) | |  |  |
| *Capronia munkii* | |  | |  | | EF413604 (15) | |  |  |
| *Capronia semiimmersa* | | KF155206; CBS_273.37 (16) | |  | | jgi\|Capse1\|scaffold_11:1-682 Assembly; CBS 273.37 (17) | | jgi\|Capse1\|358429 Transcript ID; CBS 273.37 (17) | jgi\|Capse1\|359679; CBS 273.37 (17) |
| *Ceramothyrium carniolicum* | |  | |  | | EF413628 (15) | |  |  |
| *Ceramothyrium linnaeae* | |  | |  | | MH874144 (3) | |  |  |
| *Ceramothyrium podocarpi* | |  | |  | | NG_042751 (18) | |  |  |
| *Chaetothyriomycetidae sp EF1591* | |  | | LC603025 (19) | | LC603025 (19) | |  |  |
| *Chrysosporium merdarium* | |  | |  | | MH870882 (3) | |  |  |
| *Cladophialophora carrionii* | |  | |  | | NG_055741 (20) | |  |  |
| *Cladosporium fulvum* | | jgi\|Clafu1\|scf7180000127145:490-991 Assembly; CBS 131901 (5, 21) | |  | |  | | jgi\|Clafu1\|185393 Transccript ID; CBS 131901 (5, 21) | jgi\|Clafu1\|190802; CBS 131901 (5, 21) |
| *Cladosporium halotolerans* | |  | |  | | MH876948 (3) | |  |  |
| *Cladosporium oxysporum* | |  | |  | | NG_069948 (3) | |  |  |
| *Cladosporium sphaerospermum* | | jgi\|Clasph1\|scaffold_22:2844-3603 Assembly; UM 843 (21) | |  | | jgi Clasph1\|scaffold_22:4716-5585 Assembly; UM 843 (21) | | jgi\|Clasph1\|8413 Transcript ID; UM 843 (21) | jgi\|Clasph1\|4070 Transcript ID; UM 843 (21) |
| *Coccidioides posadasii* | | CP075075; strain 'Silveira' (22) | |  | | MH877756; CBS 113855 | | jgi\|Cocpos1\|3569 Transcript ID; strain 'Silveira' (22) | jgi\|Cocpos1\|9850 Transcript ID; strain 'Silveira' (22) |
| *Coleophoma parafusiformis* | |  | |  | | KU728534 (23) | |  |  |
| *Cyphellophora europaea* | | KF155207 CBS 120392 [note, GB CBS accession wrong in GB? CBS 120393?] (23) | |  | | KC455259 CBS 101466 (24) | | jgi\|Cypeu1\|120774 Transcript ID CBS 101466 (17) | jgi\|Cypeu1\|120097 Transcript ID CBS 101466 (17) |
| *Cyphellophora musae* | |  | |  | | NG_068524 (25) | |  |  |
| *Cyphellophora reptans* | |  | |  | | NG_067426 (3) | |  |  |
| *Delphinella strobiligena* | |  | |  | | MH872074 (3) | |  |  |
| *Dothiora europaea* | |  | |  | | NG_064093 (3) | |  |  |
| *Dothiora prunorum* | |  | |  | | NG_070590 (3) | |  |  |
| *Epibryon bryophilum* | | EU940017 M2 S. Huhtinen 03/14 (TUR) (26) | |  | | EU940090 M2 S. Huhtinen 03/14 (TUR) (26) | |  |  |
| *Elsinoe banksiigena* | |  | |  | | NG_064552 (27) | |  |  |
| *Elsinoe heveae* | |  | |  | | MH869112 (3) | |  |  |
| *Elsinoe sicula* | |  | |  | | NG_069692 (3) | |  |  |
| *Endocarpon adscendens* | |  | | ON228403 (28) | | EF643751 (29) | |  |  |
| *Endocarpon pallidulum* | | DQ823104 AFTOL-ID 661 (30) | | DQ826735; AFTOL-ID 661 DQ823104 (30) | | DQ823097; AFTOL-ID 661 (30) | | jgi\|Endpal1\|1558 Transcript ID; voucher unknown (31) | jgi\|Endpal1\|7961; voucher unknown (31) |
| *Endocarpon pusillum* | | EF689837; AFTOL-ID 2279 (29) | |  | | jgi\|EndpusZ1\|scaffold_878 878:5386-4779 Assembly; Z07020 (HMAS-L-300199) (32) | | jgi\|EndpusZ1\|6407 Transcript ID; Z07020 (HMAS-L-300199)(32) | jgi\|EndpusZ1\|4315 Z07020 (HMAS-L-300199) (32) |
| *Endosporium aviarium* | |  | |  | | NG_059195 (33) | |  |  |
| *Endosporium populi-tremuloides* | |  | |  | | NG_064317 (33) | |  |  |
| *Exophiala dermatitidis* | |  | |  | | MH878057 (3) | |  |  |
| *Exophiala viscosa* | | jgi\|EurotioJF033F_1\|TRINITY_DN6262_c40_g1_i1:797-1676 EST cluster; CBS 148801 (34) | |  | | OR088060; strain JF 03-3F CBS 148801 | | EurotioJF033F_1\|615742 Transcript ID; CBS 148801 (34) | EurotioJF033F_1\|640286 Transcript ID; CBS 148801 (34) |
| *Exophiala xenobiotica* | | jgi\|Exoxe1\|scaffold_11 Assembly; CBS 118157 (17) | |  | | XR_001230708; CBS 118157 | | jgi\|Exoxe1\|144880 Transcript ID; CBS 118157 (17) | jgi\|Exoxe1\|140216; CBS 118157 (17) |
| *Fonsecaea erecta* | | KF155210; CBS 12576 (16) | |  | | MH875212; CBS 125759 (3) | | jgi\|Foner1\|10683 Transcript ID; CBS 125763 (35) | jgi\|Foner1\|73 Transcript ID; CBS 125763 (35) |
| *Fonsecaea monophora* | |  | |  | | MH878004 (3) | |  |  |
| *Fonsecaea multimorphosa* | | NG_062823; CBS 980.96 (36) | |  | | NG_057983; CBS 980.96 (16) | | jgi\|Fonmu1\|8999 Transcript ID; CBS 102226 (17) | jgi\|Fonmu1\|8092 Transcript ID; CBS 102226 (17) |
| *Fraxinicola fraxini* | |  | |  | | NG_070939 (37) | |  |  |
| *Fumiglobus pieridicola* | |  | |  | | NG_060400 (38) | |  |  |
| *Fusicladium proteae* | |  | |  | | NG_057940 (39) | |  |  |
| *Heterophaeomoniella pinifoliorum* | |  | |  | | NG_064185 (3) | |  |  |
| *Hortaea werneckii* | |  | |  | | MH875816 (3) | |  |  |
| *Knufia perforans* | |  | |  | | NG_042586 (40) | |  |  |
| *Knufia petricola* | |  | |  | | FJ358249 (7) | |  |  |
| *Lophiostoma macrostomum* v1.0_jgi | |  | |  | | EU552141 (41) | |  |  |
| *Malbranchea albolutea* | |  | |  | | MH872808 (3) | |  |  |
| *Metacapnodium ericophilum* | |  | | MW248523; K(M) 255493 (42) | |  | |  |  |
| *Metacapnodium ericophilum (as Hormiscium_ericae)* | |  | | MW376663; strain LUGO:ECC16120704 (43) | | MW376730; strain LUGO:ECC16120704 (43) | |  |  |
| *Metacapnodium neesii* JCM 39119 | | LC576695 (44) | | LC576698 (44) | | LC576694 (44) | | LC576697 (44) | LC576696 (44) |
| *Metacapnodium* sp. (as *Scorias* sp.) | |  | | MH93026; Voucher FLAS-F-64980 | |  | |  |  |
| *Minimelanolocus curvatus* | |  | |  | | NG_070383 (45) | |  |  |
| *Minutiella simplex* | |  | |  | | MN232928 (46) | |  |  |
| *Minutiella pruni-avium* | |  | |  | | MN232926 (46) | |  |  |
| *Mycosphaerella eumusae* CBS 114824 | |  | |  | | jgi\|Myceu1\|scaffold_734:5777-7378 Assembly; CBS 114824 (47) | |  |  |
| *Neoasbolisia phylicae* | |  | |  | | NG_068683 (48) | |  |  |
| *Neosorocybe pini* | |  | | NG_074422 (49) | | MT223916 (49) | |  |  |
| *Nothophaeomoniella ekebergiae* | |  | |  | | NG_076751 | |  |  |
| *Paraphaeomoniella capensis* | |  | |  | | NG_057814 (50) | |  |  |
| *Penicillium arizonense* | |  | |  | | jgi\|Penar1\|contig_57:385-1245 Assembly; CBS 141311 | |  |  |
| *Penicillium thymicola* | | jgi\|Penth1\|TR23632\|c0_g2_i1_Penth1\|TR23632\|c0_g2_i1 EST clusters; DAOMC 180753 (51) | |  | | jgi\|Penth1\|TR970\|c0_g1_i1:293-1172 EST cluster; DAOMC 180753 (51) | | jgi\|Penth1\|196475 Transcript ID; DAOMC 180753 (51) | jgi\|Penth1\|197328 Transcript ID Transcript ID; DAOMC 180753 (51) |
| *Phacidium lauri* | |  | |  | | NG*_*070581 (3) | |  |  |
| *Phaeomoniella chlamydospora* | | AB278179; CBS 239.74 (52) | |  | | NG_066265; CBS 229.95 (3) | | jgi\|Phach1\|5140 Transcript ID; UCR-PC4 (53) | jgi\|Phach1\|1316 Transcript ID; UCR-PC4 (53) |
| *Phragmocapnias asiticus* | |  | |  | | JN832612 (54) | |  |  |
| *Phragmocapnias betle* | |  | |  | | JN832606 (54) | |  |  |
| *Phragmocapnias siamensis* | |  | |  | | NG_070828 (54) | |  |  |
| *Piedraia hortae* | | AY016349; CBS 480.64 (55) | |  | | AY016366; CBS 480.64 (55) | | jgi\|Pieho1_1\|256169 Transcript ID; CBS 480.64 (1) | jgi\|Pieho1_1\|252915 Transcript ID; CBS 480.64 (1) |
| *Pleostigma alpinum* | | KT263370; isolate A1025 specimen holotype GZU:LMCC0348 (56) | |  | | KT263337; isolate A1025 KT270649; isolate A945 (56) | |  |  |
| *Pleostigma frigidum* | | KT270704; isolate A980, from holotype GZU LMCC0317 (56) | |  | | KT270672; isolate A980, from holotype GZU LMCC0317 (56) | |  |  |
| *Pleostigma lichenophilum* | | KT270726 A926 (56) | |  | | KT270655; isolate A952 holotype LMCC0289  (56) | |  |  |
| *Pleostigmataceae sp.* | |  | | OQ920989; isolate L3258  (57) | | OQ955703  isolate L3258 (57) | |  |  |
| *Pleostigmataceae* sp. | |  | | OQ921052; isolate L3809 (57) | | OQ955706; isolate L3809 (57) | |  |  |
| *Polychaeton citri* | | jgi\|Polci1\|Locus187v1rpkm630_21 EST cluster; CBS 116435 (1) | |  | | OM238161 CBS 116435 | | jgi\|Polci1\|284695 Transcript ID; CBS 116435 (1) | jgi\|Polci1\|287784 Transcript ID; CBS 116435 (1) |
| *Protoventuria alpina* | |  | |  | | EU035446 (58) | |  |  |
| *Pseudoanungitea vaccinii* | |  | |  | | MH107958 (59) | |  |  |
| *Pyrenula aspistea* | |  | |  | | EF411063 (15) | |  |  |
| *Pyrenula confinis* | |  | |  | | OP584266 (60) | |  |  |
| *Pyrenula nitida* | |  | |  | | AY607737 (61) | |  |  |
| *Pyrenula ochraceoflavens* | |  | |  | | OP584269 (60) | |  |  |
| *Rasutoria pseudotsugae* | |  | |  | | EF114704 (62) | |  |  |
| *Rhinocladiella mackenziei* | |  | |  | | AF050288 CBS 650.93 (63) | | jgi\|Rhima1\|4389 Transcript ID; CBS 650.93 (64) | jgi\|Rhima1\|2697 Transcript ID; CBS 650.93 (64) |
| *Scorias leucadendri* | |  | |  | | JQ044456 (65) | |  |  |
| *Scorias spongiosa* | |  | |  | | MH866910 (3) | |  |  |
| *Septonema fasciculare* | |  | |  | | MH876104 (3) | |  |  |
| *Sorocybe resinae* | |  | | EU030275; DAOM 239134 (66) | | EU030277; DAOM 239134  (66) | |  |  |
| Sorocybe oblongispora | |  | | NR_166300 (67) | | MN114118 | |  |  |
| *Staurothele immersa* | |  | |  | | EF643777 (29) | |  |  |
| *Staurothele rupifraga* | |  | |  | | EU598693 (68) | |  |  |
| *Thysanorea yunnanensis* | |  | |  | | NG_070385 (45) | |  |  |
| *Trichomerium eucalypti* | |  | |  | | NG_058525 (69) | |  |  |
| *Trichomerium syzygii* | |  | |  | | MT223936 (49) | |  |  |
| *Triposporium deviatum* | |  | |  | | KY853537 (70) | |  |  |
| *Venturia cerasi* | |  | |  | | EU035452 (58) | |  |  |
| *Venturia inaequalis* | | EF114737 ATCC 60070 (62) | |  | | EU035460 CBS 535.76 (58) | | jgi\|Venin1\|21805 Transcript ID; voucher unspecified (71) | jgi\|Venin1\|14545 Transcript ID; voucher unspecified (71) |
| *Venturia maculiformis* | |  | |  | | MH868784 (3) | |  |  |
| *Veronaea botryosa* | |  | |  | | MH875936 (3) | |  |  |
| *Verrucaria luchunensis* | |  | | OM228830 (72) | | OM228830 (72) | |  |  |
| *Verrucaria modica* | |  | | NR_173806 (73) | |  | |  |  |
| *Verrucaria muralis* | | EF689878; AFTOL-ID 2265 (29) | | KY697147; DUKE Heidmarsson 2216 (74) | | KY773262; DUKE Heidmarsson 2216 (74) | |  |  |
| *Verrucula inconnexaria* | | EF689892; AFTOL-ID 2307 (29) | | EU006538; AFTOL-ID 2307  (75) | | EF643821; AFTOL-ID 2307 (29) | |  |  |
| *Willeya laevigata* | |  | |  | | NG_059966 (76) | |  |  |
| *Willeya protrudens* | |  | |  | | NG_059964 (76) | |  |  |
| *Zasmidium cellare* | |  | |  | | NG_057791 (77) | |  |  |
|  |  | |  | |  | |  |  |  |

*****Accession numbers beginning with two letters are from GenBank; accession numbers beginning with 'NG_' are GenBank reference sequences, and accession numbers beginning with 'jgi' are either transcript IDs or EST cluster IDs from the Joint Genome Institute (JGI)'s databases <https://genome.jgi.doe.gov/portal/>. Transcripts may be accessed by searching via JGI's home page for the genome of the species/strain. EST clusters may also be accessed via the JGI species/strain home page by using a BLAST search through the organism's EST clusters data, using the sequence provided in Supplementary Material 2 as a query. Fungal strain identifiers follow the sequence accession number.

**References to previously published data**

1. Haridas S, Albert R, Binder M, Bloem J, LaButti K, Salamov A, et al. 101 Dothideomycetes genomes: A test case for predicting lifestyles and emergence of pathogens. Stud Mycol. 2020;96:141-53:<https://doi.org/10.1016/j.simyco.2020.01.003>.

2. Mesny F, Miyauchi S, Thiergart T, Pickel B, Atanasova L, Karlsson M, et al. Genetic determinants of endophytism in the *Arabidopsis* root mycobiome. Nat Commun. 2021;12(1):7227:<https://doi.org/10.1038/s41467-021-27479-y>.

3. Vu D, Groenewald M, de Vries M, Gehrmann T, Stielow B, Eberhardt U, et al. Large-scale generation and analysis of filamentous fungal DNA barcodes boosts coverage for kingdom fungi and reveals thresholds for fungal species and higher taxon delimitation. Stud Mycol. 2019;92:135-54:<https://doi.org/10.1016/j.simyco.2018.05.001>.

4. Woudenberg JH, Groenewald JZ, Binder M, Crous PW. *Alternaria* redefined. Stud Mycol. 2013;75(1):171-212:<https://doi.org/10.3114/sim0015>.

5. Ohm RA, Feau N, Henrissat B, Schoch CL, Horwitz BA, Barry KW, et al. Diverse lifestyles and strategies of plant pathogenesis encoded in the genomes of eighteen Dothideomycetes fungi. PLoS Pathog. 2012;8(12):e1003037:<https://doi.org/10.1371/journal.ppat.1003037>.

6. Pitt JI, Lantz H, Pettersson OV, Leong SL. *Xerochrysium* gen. nov. and *Bettsia*, genera encompassing xerophilic species of *Chrysosporium*. IMA Fungus. 2013;4(2):229-41:<https://doi.org/10.5598/imafungus.2013.04.02.08>.

7. Gueidan C, Villaseñor CR, de Hoog GS, Gorbushina AA, Untereiner WA, Lutzoni F. A rock-inhabiting ancestor for mutualistic and pathogen-rich fungal lineages. Stud Mycol. 2008;61:111-9:<https://doi.org/10.3114/sim.2008.61.11>.

8. Han JG, Hosoya T, Sung GH, Shin HD. Phylogenetic reassessment of Hyaloscyphaceae sensu lato (Helotiales, Leotiomycetes) based on multigene analyses. Fungal Biol. 2014;118(2):150-67:<https://doi.org/10.1016/j.funbio.2013.11.004>.

9. Gianoulis TA, Griffin MA, Spakowicz DJ, Dunican BF, Alpha CJ, Sboner A, et al. Genomic analysis of the hydrocarbon-producing, cellulolytic, endophytic fungus *Ascocoryne sarcoides*. PLoS Genet. 2012;8(3):e1002558:<https://doi.org/10.1371/journal.pgen.1002558>.

10. Kjærbølling I, Vesth TC, Frisvad JC, Nybo JL, Theobald S, Kuo A, et al. Linking secondary metabolites to gene clusters through genome sequencing of six diverse *Aspergillus* species. Proc Natl Acad Sci U S A. 2018;115(4):E753-e61:<https://doi.org/10.1073/pnas.1715954115>.

11. Gostinčar C, Ohm RA, Kogej T, Sonjak S, Turk M, Zajc J, et al. Genome sequencing of four *Aureobasidium pullulans* varieties: biotechnological potential, stress tolerance, and description of new species. BMC Genomics. 2014;15:549:<https://doi.org/10.1186/1471-2164-15-549>.

12. Staats M, van Kan JA. Genome update of *Botrytis cinerea* strains B05.10 and T4. Eukaryot Cell. 2012;11(11):1413-4:<https://doi.org/10.1128/ec.00164-12>.

13. Amselem J, Cuomo CA, van Kan JA, Viaud M, Benito EP, Couloux A, et al. Genomic analysis of the necrotrophic fungal pathogens *Sclerotinia sclerotiorum* and *Botrytis cinerea*. PLoS Genet. 2011;7(8):e1002230:<https://doi.org/10.1371/journal.pgen.1002230>.

14. Lumbsch HT, Schmitt I, Lindemuth R, Miller A, Mangold A, Fernandez F, et al. Performance of four ribosomal DNA regions to infer higher-level phylogenetic relationships of inoperculate euascomycetes (Leotiomyceta). Mol Phylogenet Evol. 2005;34(3):512-24:<https://doi.org/10.1016/j.ympev.2004.11.007>.

15. Geiser DM, Gueidan C, Miadlikowska J, Lutzoni F, Kauff F, Hofstetter V, et al. Eurotiomycetes: Eurotiomycetidae and Chaetothyriomycetidae. Mycologia. 2006;98(6):1053-64:<https://doi.org/10.3852/mycologia.98.6.1053>.

16. Vicente VA, Najafzadeh MJ, Sun J, Gomes RR, Robl D, Marques SG, et al. Environmental siblings of black agents of human chromoblastomycosis. Fungal Diversity. 2014;65(1):47-63:<https://doi.org/10.1007/s13225-013-0246-5>.

17. Teixeira MM, Moreno LF, Stielow BJ, Muszewska A, Hainaut M, Gonzaga L, et al. Exploring the genomic diversity of black yeasts and relatives (Chaetothyriales, Ascomycota). Stud Mycol. 2017;86:1-28:<https://doi.org/10.1016/j.simyco.2017.01.001>.

18. Crous PW, Summerell BA, Shivas RG, Burgess TI, Decock CA, Dreyer LL, et al. Fungal Planet description sheets: 107-127. Persoonia. 2012;28:138-82:<https://doi.org/10.3767/003158512x652633>.

19. Baba T, Hirose D. Slow-growing fungi belonging to the unnamed lineage in Chaetothyriomycetidae form hyphal coils in vital ericaceous rhizodermal cells in vitro. Fungal Biology. 2021;125(12):1026-35:<https://doi.org/10.1016/j.funbio.2021.07.003>.

20. Attili-Angelis D, Duarte A, Pagnocca F, Nagamoto N, De Vries M, Stielow J, et al. Novel *Phialophora* species from leaf-cutting ants (tribe *Attini*). Fungal Diversity. 2014;65:65-75:<https://doi.org/10.1007/s13225-013-0275-0>.

21. de Wit PJ, van der Burgt A, Ökmen B, Stergiopoulos I, Abd-Elsalam KA, Aerts AL, et al. The genomes of the fungal plant pathogens *Cladosporium fulvum* and *Dothistroma septosporum* reveal adaptation to different hosts and lifestyles but also signatures of common ancestry. PLoS Genet. 2012;8(11):e1003088:<https://doi.org/10.1371/journal.pgen.1003088>.

22. Neafsey DE, Barker BM, Sharpton TJ, Stajich JE, Park DJ, Whiston E, et al. Population genomic sequencing of *Coccidioides* fungi reveals recent hybridization and transposon control. Genome Res. 2010;20(7):938-46:<https://doi.org/10.1101/gr.103911.109>.

23. Crous PW, Groenewald JZ. They seldom occur alone. Fungal Biol. 2016;120(11):1392-415:<https://doi.org/10.1016/j.funbio.2016.05.009>.

24. Réblová M, Untereiner WA, Réblová K. Novel evolutionary lineages revealed in the Chaetothyriales (fungi) based on multigene phylogenetic analyses and comparison of its secondary structure. PLoS One. 2013;8(5):e63547:<https://doi.org/10.1371/journal.pone.0063547>.

25. Gao L, Ma Y, Zhao W, Wei Z, Gleason ML, Chen H, et al. Three New Species of Cyphellophora (Chaetothyriales) Associated with Sooty Blotch and Flyspeck. PLoS One. 2015;10(9):e0136857:<https://doi.org/10.1371/journal.pone.0136857>.

26. Stenroos S, Laukka T, Huhtinen S, Döbbeler P, Myllys L, Syrjänen K, et al. Multiple origins of symbioses between ascomycetes and bryophytes suggested by a five‐gene phylogeny. Cladistics. 2010;26(3):281-300:<https://doi.org/10.1007/s13225-013-0275-0>.

27. Crous PW, Wingfield MJ, Burgess TI, Hardy GESJ, Gené J, Guarro J, et al. Fungal Planet description sheets: 716&#8211;784. Persoonia - Molecular Phylogeny and Evolution of Fungi. 2018;40(1):239-392:<https://doi.org/10.3767/persoonia.2018.40.10>.

28. Malicek J. Lichens recorded during the bryological and lichenological days in the Kokořín region. Bryonora (Praha). 2022;69

29. Gueidan C, Roux C, Lutzoni F. Using a multigene phylogenetic analysis to assess generic delineation and character evolution in Verrucariaceae (Verrucariales, Ascomycota). Mycological Research. 2007;111(10):1145-68:<https://doi.org/https://doi.org/10.1016/j.mycres.2007.08.010>.

30. James TY, Kauff F, Schoch CL, Matheny PB, Hofstetter V, Cox CJ, et al. Reconstructing the early evolution of Fungi using a six-gene phylogeny. Nature. 2006;443(7113):818-22:<https://doi.org/10.1038/nature05110>.

31. McDonald TR, Mueller O, Dietrich FS, Lutzoni F. High-throughput genome sequencing of lichenizing fungi to assess gene loss in the ammonium transporter/ammonia permease gene family. BMC Genomics. 2013;14:225:<https://doi.org/10.1186/1471-2164-14-225>.

32. Wang YY, Liu B, Zhang XY, Zhou QM, Zhang T, Li H, et al. Genome characteristics reveal the impact of lichenization on lichen-forming fungus *Endocarpon pusillum* Hedwig (Verrucariales, Ascomycota). BMC Genomics. 2014;15:34:<https://doi.org/10.1186/1471-2164-15-34>.

33. Tsuneda A, Davey M, Hambleton S, Currah R. *Endosporium*, a new endoconidial genus allied to the Myriangiales. Botany. 2008;86(9):1020-33:<https://doi.org/10.1139/B08-054>.

34. Carr EC, Barton Q, Grambo S, Sullivan M, Renfro CM, Kuo A, et al. Characterization of a novel polyextremotolerant fungus, *Exophiala viscosa*, with insights into its melanin regulation and ecological niche. G3 Genes|Genomes|Genetics. 2023;13(8):jkad110:<https://doi.org/10.1093/g3journal/jkad110>.

35. Vicente VA, Weiss VA, Bombassaro A, Moreno LF, Costa FF, Raittz RT, et al. Comparative genomics of sibling species of *Fonsecaea* associated with human chromoblastomycosis. Front Microbiol. 2017;8:1924:<https://doi.org/10.3389/fmicb.2017.01924>.

36. Najafzadeh MJ, Vicente VA, Sun J, Meis JF, de Hoog GS. *Fonsecaea multimorphosa* sp. nov, a new species of Chaetothyriales isolated from a feline cerebral abscess. Fungal Biol. 2011;115(10):1066-76:<https://doi.org/10.1016/j.funbio.2011.06.007>.

37. Shen M, Zhang J, Zhao L, Groenewald J, Crous P, Zhang Y. Venturiales. Studies in Mycology. 2020;96:185-308:<https://doi.org/10.1016/j.simyco.2020.03.001>.

38. Bose T, Reynolds DR, Berbee ML. Common, unsightly and until now undescribed: *Fumiglobus pieridicola* sp. nov., a sooty mold infesting *Pieris japonica* from western North America. Mycologia. 2014;106(4):746-56:<https://doi.org/10.3852/13-288>.

39. Crous PW, Summerell BA, Swart L, Denman S, Taylor JE, Bezuidenhout CM, et al. Fungal pathogens of Proteaceae. Persoonia. 2011;27:20-45:<https://doi.org/10.3767/003158511x606239>.

40. Tsuneda A, Hambleton S, Currah R. The anamorph genus *Knufia* and its phylogenetically allied species in *Coniosporium*, *Sarcinomyces*, and *Phaeococcomyces*. Botany. 2011;89(8):523-36:<https://doi.org/10.1139/b11-041>.

41. Marincowitz S, Crous PW, Groenewald JZ, Wingfield MJ. Microfungi occurring on Proteaceae in the fynbos: CBS-KNAW Fungal Biodiversity Centre; 2008.

42. Hawksworth DL, Boluda CG. The enigma of Link's *Sphaeria ericophila*: nomenclature, taxonomy, molecular phylogeny, and implications for the placement of *Metacapnodium*. 2020:<https://doi.org/10.13341/j.jfr.2020.8005>.

43. Alonso Díaz J, Rigueiro Rodríguez A. Catálogo da macromicobiota das montañas do Courel (Galicia, NO España). Universidade de Santiago de Compostela, Lugo. 2020

44. Sugiyama J, Nam K-O, Hosoya T. *Metacapnodium neesii*: a new combination for a metacapnodiaceous sooty mould and its phylogenetic position inferred from DNA sequences. 2020:<https://doi.org/10.13341/j.jfr.2020.8002>.

45. Liu XY, Udayanga D, Luo ZL, Chen LJ, Zhou DQ, Su HY, et al. Backbone tree for Chaetothyriales with four new species of *Minimelanolocus* from aquatic habitats. Fungal Biol. 2015;119(11):1046-62:<https://doi.org/10.1016/j.funbio.2015.08.005>.

46. Bien S, Damm U. *Arboricolonus simplex* gen. et sp. nov. and novelties in *Cadophora*, *Minutiella* and *Proliferodiscus* from *Prunus* wood in Germany. MycoKeys. 2020;63:119:<https://doi.org/10.3897/mycokeys.63.46836>.

47. Chang TC, Salvucci A, Crous PW, Stergiopoulos I. Comparative genomics of the sigatoka disease complex on banana suggests a link between parallel evolutionary changes in *Pseudocercospora fijiensis* and *Pseudocercospora eumusae* and increased virulence on the banana host. PLoS Genet. 2016;12(8):e1005904:<https://doi.org/10.1371/journal.pgen.1005904>.

48. Abdollahzadeh J, Groenewald J, Coetzee M, Wingfield M, Crous P. Evolution of lifestyles in Capnodiales. Studies in mycology. 2020;95(1):381-414:<https://doi.org/10.1016/j.simyco.2020.02.004>.

49. Crous PW, Wingfield MJ, Schumacher RK, Akulov A, Bulgakov TS, Carnegie AJ, et al. New and Interesting Fungi. 3. Fungal Systematics and Evolution. 2020;6(1):157-231:<https://doi.org/10.3114/fuse.2020.06.09>.

50. Crous PW, Wood AR, Okada G, Groenewald JZ. Foliicolous microfungi occurring on Encephalartos. Persoonia. 2008;21:135-46:<https://doi.org/10.3767/003158508x380612>.

51. Nguyen HDT, McMullin DR, Ponomareva E, Riley R, Pomraning KR, Baker SE, et al. Ochratoxin A production by *Penicillium thymicola*. Fungal Biol. 2016;120(8):1041-9:<https://doi.org/10.1016/j.funbio.2016.04.002>.

52. Yaguchi T, Sano A, Yarita K, Suh MooKyu SM, Nishimura K, Udagawa S-i. A new species of *Cephalotheca* isolated from a Korean patient. Mycotaxon. 2006;96:309-22

53. Morales-Cruz A, Amrine KC, Blanco-Ulate B, Lawrence DP, Travadon R, Rolshausen PE, et al. Distinctive expansion of gene families associated with plant cell wall degradation, secondary metabolism, and nutrient uptake in the genomes of grapevine trunk pathogens. BMC Genomics. 2015;16(1):469:<https://doi.org/10.1186/s12864-015-1624-z>.

54. Chomnunti P, Schoch CL, Aguirre-Hudson B, Ko-Ko TW, Hongsanan S, Jones EB, et al. Capnodiaceae. Fungal Divers. 2011;51(1):103-34:<https://doi.org/10.1007/s13225-011-0145-6>.

55. Lumbsch HT, Lindemuth R. Major lineages of Dothideomycetes (Ascomycota) inferred from SSU and LSU rDNA sequences. Mycological Research. 2001;105(8):901-8:<https://doi.org/10.1017/S0953756201004385>.

56. Muggia L, Fleischhacker A, Kopun T, Grube M. Extremotolerant fungi from alpine rock lichens and their phylogenetic relationships. Fungal Divers. 2016;76:119-42:<https://doi.org/10.1007/s13225-015-0343-8>.

57. Cometto A, Leavitt SD, Grube M, De Hoog S, Muggia L. Tackling fungal diversity in lichen symbioses: molecular and morphological data recognize new lineages in Chaetothyriales (Eurotiomycetes, Ascomycota). Mycological Progress. 2023;22(8):53:<https://doi.org/10.1007/s11557-023-01901-9>.

58. Crous PW, Schubert K, Braun U, de Hoog GS, Hocking AD, Shin HD, et al. Opportunistic, human-pathogenic species in the Herpotrichiellaceae are phenotypically similar to saprobic or phytopathogenic species in the Venturiaceae. Stud Mycol. 2007;58:185-217:<https://doi.org/10.3114/sim.2007.58.07>.

59. Crous P, Schumacher RK, Wingfield MJ, Akulov A, Denman S, Roux J, et al. New and interesting fungi. 1. Fungal Systematics and Evolution. 2018;1(1):169-215:<https://doi.org/10.3114/fuse.2018.01.08>.

60. Miranda-González R, Bungartz F, Lücking R, Gaya E, de Oliveira Mendonça C, Viñas-Portilla C, et al. Phylogeny of the *Pyrenula ochraceoflava* group (Pyrenulaceae) reveals near-cryptic diversification and the inclusion of the *Mazaediothecium album* aggregate. The Bryologist. 2022;125(4):541-57:<https://doi.org/10.1639/0007-2745-125.4.541>.

61. Schmitt I, Mueller G, Lumbsch HT. Ascoma morphology is homoplaseous and phylogenetically misleading in some pyrenocarpous lichens. Mycologia. 2005;97(2):362-74:<https://doi.org/10.3852/mycologia.97.2.362>.

62. Winton LM, Stone JK, Hansen EM, Shoemaker RA. The systematic position of Phaeocryptopus gaeumannii. Mycologia. 2007;99(2):240-52:<https://doi.org/10.3852/mycologia.99.2.240>.

63. Untereiner WA, Naveau FA. Molecular systematics of the Herpotrichiellaceae with an assessment of the phylogenetic positions of *Exophiala dermatitidis* and *Phialophora americana*. Mycologia. 1999;91(1):67-83:<https://doi.org/10.1080/00275514.1999.12060994>.

64. Moreno LF, Ahmed AAO, Brankovics B, Cuomo CA, Menken SBJ, Taj-Aldeen SJ, et al. Genomic understanding of an infectious brain disease from the desert. G3 (Bethesda). 2018;8(3):909-22:<https://doi.org/10.1534/g3.117.300421>.

65. Crous PW, Summerell B, Shivas RG, Romberg M, Mel'nik V, Verkley G, et al. Fungal Planet description sheets: 92–106. Persoonia-Molecular Phylogeny and Evolution of Fungi. 2011;27(1):130-62:<https://doi.org/10.3767/003158511X617561>.

66. Seifert KA, Hughes SJ, Boulay H, Louis-Seize G. Taxonomy, nomenclature and phylogeny of three cladosporium-like hyphomycetes, *Sorocybe resinae,* *Seifertia azaleae* and the *Hormoconis* anamorph of *Amorphotheca resinae*. Stud Mycol. 2007;58:235-45:<https://doi.org/10.3114/sim.2007.58.09>.

67. Crous PW, Wingfield MJ, Lombard L, Roets F, Swart WJ, Alvarado P, et al. Fungal Planet description sheets: 951-1041. Persoonia. 2019;43:223-425:<https://doi.org/10.3767/persoonia.2019.43.06>.

68. Savić S, Tibell L, Gueidan C, Lutzoni F. Molecular phylogeny and systematics of *Polyblastia* (Verrucariaceae, Eurotiomycetes) and allied genera. Mycol Res. 2008;112(Pt 11):1307-18:<https://doi.org/10.1016/j.mycres.2008.05.002>.

69. Crous PW, Wingfield MJ, Burgess TI, Carnegie AJ, Hardy G, Smith D, et al. Fungal Planet description sheets: 625-715. Persoonia. 2017;39:270-467:<https://doi.org/10.3767/persoonia.2017.39.11>.

70. Hernández-Restrepo M, Gené J, Castañeda-Ruiz RF, Mena-Portales J, Crous PW, Guarro J. Phylogeny of saprobic microfungi from Southern Europe. Stud Mycol. 2017;86:53-97:<https://doi.org/10.1016/j.simyco.2017.05.002>.

71. Deng CH, Plummer KM, Jones DAB, Mesarich CH, Shiller J, Taranto AP, et al. Comparative analysis of the predicted secretomes of Rosaceae scab pathogens *Venturia inaequalis* and *V. pirina* reveals expanded effector families and putative determinants of host range. BMC Genomics. 2017;18(1):339:<https://doi.org/10.1186/s12864-017-3699-1>.

72. Orange A, Chhetri SG. Verrucariaceae from Nepal. The Lichenologist. 2022;54(3-4):139-74:<https://doi.org/10.1017/S0024282922000160>.

73. Pykälä J, Launis A, Myllys L. Taxonomy of the *Verrucaria kalenskyi*-*V. xyloxena* species complex in Finland. 2019:<https://doi.org/10.1127/nova_hedwigia/2019/0553>.

74. Heiðmarsson S, Gueidan C, Miadlikowska J, Lutzoni F. Multi-locus phylogeny supports the placement of *Endocarpon pulvinatum* within *Staurothele* s. str.(lichenised ascomycetes, Eurotiomycetes, Verrucariaceae). Phytotaxa. 2017;306(1):37-48:<https://doi.org/> 10.11646/phytotaxa.306.1.3.

75. Navarro-Rosinés P, Roux C, Gueidan C. La genroj *Verrucula* kaj *Verruculopsis* (Verrucariaceae, Verrucariales). Bull Soc Linn Provence. 2007;58:133-80

76. Gueidan C, Truong VD, Lu NT. Phylogeny and taxonomy of *Staurothele* (Verrucariaceae, lichenized ascomycetes) from the karst of northern Vietnam. The Lichenologist. 2014;46(4):515-33:<https://doi.org/10.1017/S0024282914000048>.

77. Arzanlou M, Groenewald J, Gams W, Braun U, Shin H-D, Crous PW. Phylogenetic and morphotaxonomic revision of *Ramichloridium* and allied genera. Studies in Mycology. 2007;58(1):57-93:<https://doi.org/10.3114/sim.2007.58.03>.
