# Supplementary material for: Taxonomy and phylogeny of the epiphytic sooty molds in family Metacapnodiaceae (class Eurotiomycetes, subclass Chaetothyriomycetidae)
Source: MycoKeys. 2026 Mar 4;129:163–212. doi: 10.3897/mycokeys.129.178067 (PMC13291639; doi:10.3897/mycokeys.129.178067)

**Table S2**. Phylogenetic analyses of concatenated and individual loci; details and parameters						
	ITS	SSU, LSU, *ef1-a*, *rpb2* concatenated	SSU	LSU	*ef1-a*	*rpb2*
No. of sequences	34	122	32	121	28	25
No. of *Metacapnodium* sequences	20	7	2	7	3	1
Length of alignment	545	3910	915	801	1395	799
Invariant sites	51.19%	see single loci	80%	51.19%	codon1/2/3: 74%/84%/17%	codon1/2/3: 33%/48%/7%
No. of patterns	329	1977	252	464	codon1/2/3: 134/89/377	codon1/2/3: 214/183/264
gaps/missing data in alignment[1]	25.86%	4% for LSU, ~80% for other loci	19.64%	3.59%	10%	14%
raxmlGUI log-likelihood, best tree of 20 from random starting trees	-4196	-38,641	-3235	-12968	-10,840	-12,035
raxmlGUI bootstrap autoMRE replicate #		250	1000	550	1000	400
Model substitution (AIC)[2,3]	BIC TN93FE+I+G4	TIM2+I+G4	TIM1+I+G4	GTR+I+G4	GTR+FO+I+G4m	GTR+I+G
MrBayes # of generations	10,000,000	10,000,000				
Bayesian statistics	Burnin 25%, average potential scale reduction factor = 1.003, split frequency average 0.004, all ESS >140	Burnin 25%, average potential scale reduction factor = 1.000, split frequency avarage 0.009, all ESS >286				
Figures	Figure 2	Figure 3	Supplementary material 3. Figure S2	Supplementary material 3. Figure S3	Supplementary material 3. Figure S4	Supplementary material 3. Figure S5
Input	Supplementary material 2. Six alignments	Supplementary material 2. Six alignments	Supplementary material 2. Six alignments	Supplementary material 2. Six alignments	Supplementary material 2. Six alignments	Supplementary material 2. Six alignments
Distances	Supplementary material 3, S1					
[1]High proprtions of missing data from loci other than LSU was due to taxon sampling limitations						
[2]TIM3: AC=CG, AT=GT; TIM2: AC=AT, CG=GT; TIM1 AC=GT, AT=CG; all TIM models assume unequal base frequencies; I: maximum likelihood estimate of proportion of invariant sites; g4m: uses mean of four discrete rate categories to approximate a gamma distribution of rate heterogeneity; GTR: General time reversable model; FO uses a maximum likelihood estimate of base frequencies						
[3] I: maximum likelihood estimate of proportion of invariant sites; g4m: uses mean of four discrete rate categories to approximate a gamma distribution of rate heterogeneity; GTR: General time reversable model; FO uses a maximum likelihood estimate of base frequencies						

Figure S1. Pairwise distance matrix among ITS sequences *Metacapnodium* ITS sequences

	sp as Scorias sp MH930326	adamantinum OR532928	neesii LC576698	cf moniliforme OR532924	aff moniliforme OR532939	moniliforme OR532935	moniliforme OR532936	dingleyae OR532931	dingleyae OR532929	dingleyae OR532930	australis com nov OR532937	vancouverensis sp nov OR532925	sp OR532932	sp OR532933	stanhughesii sp nov OR532926	ericophilum as Hormiscium ericae MW376663	ericophilum MW248525	ericophilum OR532934	M. spongiosum OR532927	juniperi OR532938	
Specimen	FLAS-F-64980	OSC169460		UBC F33050	E01043362	DAOM 226251	DAOM 234317	O F293899	O F201720	O F281492	DAOM 106914	UBC F35816	O F201613	DAOM 239041	UBC F35817		DAOM 234182	DAOM 234182	OSC 135428	E 01043363	
Specimen collection date	4/1/17	8/1/00	na	4/3/16	3/13/23	8/17/99	6/1/90	7/26/10	2/2/03	3/15/03	1963-04-026	7/22/21	12/11/02	9/25/03	7/2/21	na	11/4/22	11/4/22	1/20/09	2/15/23	
#base pairs*	483	150	479	484	280	181	162	365	400	405	181	400	399	181	405	479	346	402	359	377	
	0.0	4.0	14.4	18.6	11.2	9.5	10.0	11.1	11.1	11.0	5.0	11.4	9.9	7.8	10.6	15.2	11.8	11.9	9.9	11.1	sp as Scorias sp MH930326
		0.0	4.0	6.7	2.3	5.3	6.2	3.5	3.3	3.3	3.3	4.7	4.0	4.0	4.0	6.0	5.5	6.0	2.2	4.1	adamantinum OSC169460 OR532928
			0.0	9.1	4.7	5.5	6.2	4.7	4.8	4.8	1.7	5.0	5.0	3.3	4.0	12.8	11.1	11.8	11.0	6.0	neesii LC576698
				0.0	5.4	1.7	2.5	8.5	9.1	9.1	7.2	9.8	9.6	6.6	9.8	14.1	14.2	14.1	12.4	10.0	cf moniliforme OR532924
					0.0	1.6	1.6	6.1	6.1	6.1	0.8	6.5	5.8	0.8	5.4	10.5	10.5	10.5	9.4	7.2	aff moniliforme OR532939
						0.0	1.2	2.7	5.0	5.0	5.5	5.5	3.9	5.0	7.2	8.8	8.6	8.8	2.9	6.0	moniliforme OR532935
							0.0	3.4	4.9	4.9	5.6	6.2	4.9	5.6	6.8	9.3	9.3	9.3	2.9	6.6	moniliforme OR532936
								0.0	0.0	0.0	0.0	5.8	5.2	0.7	4.7	12.7	11.3	12.7	11.6	6.9	dingleyae OR532931
									0.0	0.0	0.6	5.5	5.3	2.8	5.0	12.6	11.7	12.9	11.6	7.1	dingleyae OR532929
										0.0	0.6	5.5	5.3	2.8	5.0	12.6	11.7	12.9	11.6	7.0	dingleyae OR532930
											0.0	2.8	2.2	3.3	2.2	5.5	5.3	6.1	1.4	3.3	australis com nov OR532937
												0.0	3.5	3.3	5.3	11.6	10.8	11.6	11.0	6.0	vancouverensis sp nov OR532925
													0.0	1.1	5.5	10.6	10.8	10.6	9.6	6.5	sp OR532932
														0.0	5.0	6.6	6.0	6.6	1.4	4.0	sp OR532933
															0.0	12.1	10.9	11.8	10.7	5.4	stanhughesii sp nov OR532926
																0.0	0.0	0.3	2.0	10.8	ericophilum as Hormiscium ericae MW376663
																	0.0	0.0	2.1	9.9	ericophilum MW248525
																		0.0	2.0	10.8	ericophilum OR532934
																			0.0	10.7	novae zelandiae com nov OR532927
																				0	juniperi OR532938

*Where fewer than total number of bp in the sequence record, data from gene regions 5' and 3' to the ITS regions have been excluded from alignment.

Figure S2. SSU 32 taxa 915 sites

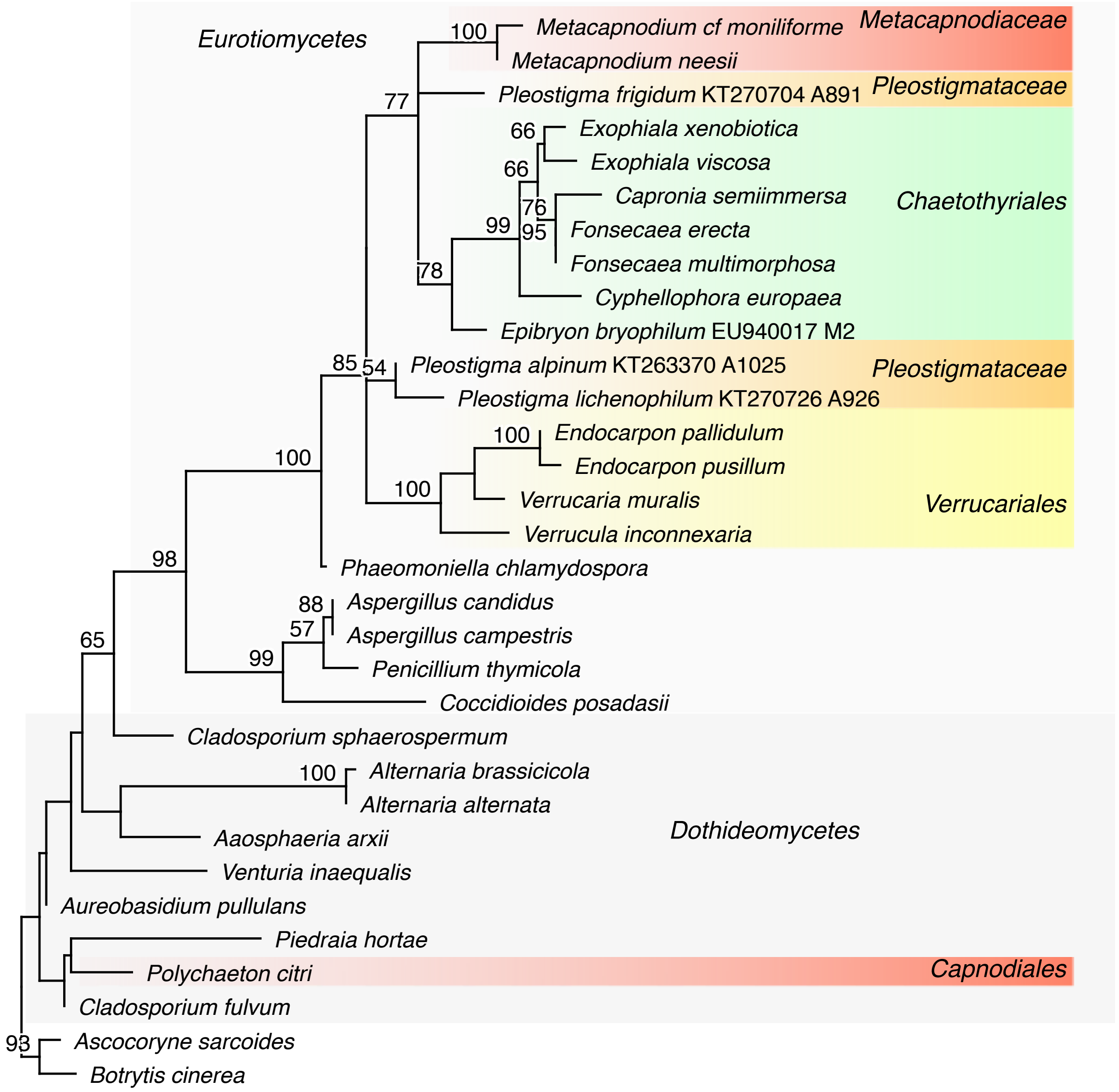

Figure S3. LSU 121 taxa 801 sites

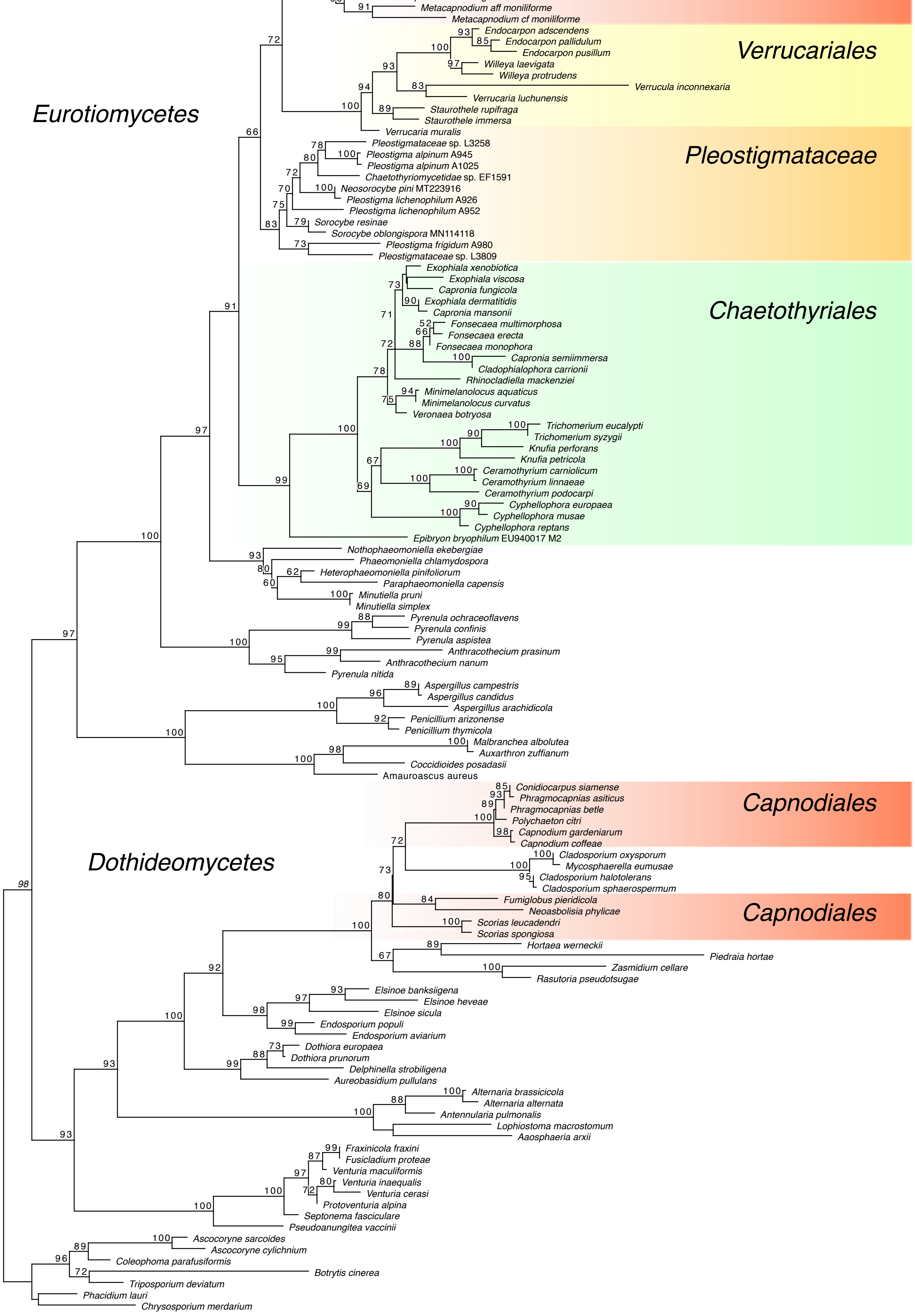

Figure S4. *ef1-alpha* 28 taxa, 1395 sites

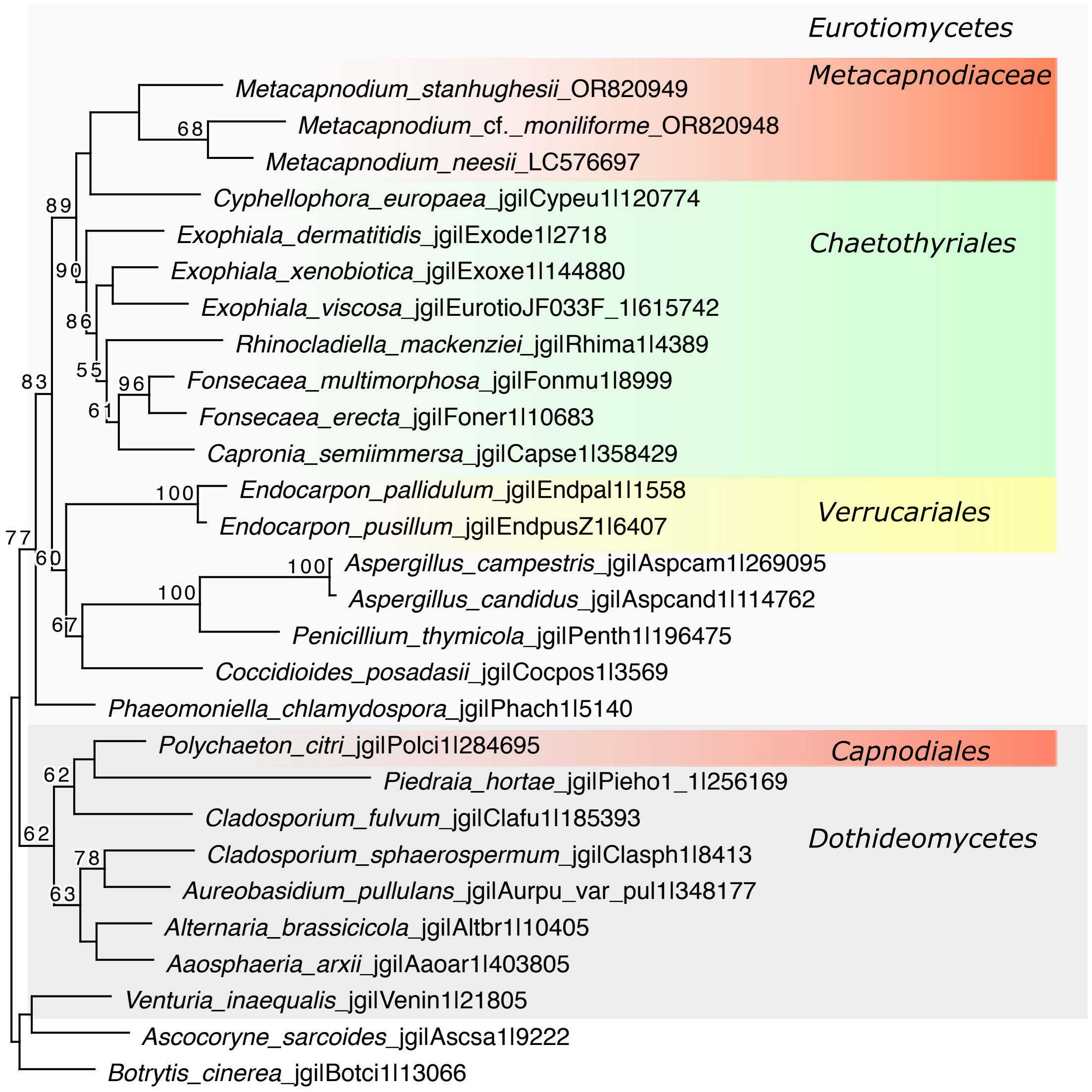

Figure S5. *Rpb2* 25 taxa,799 sites

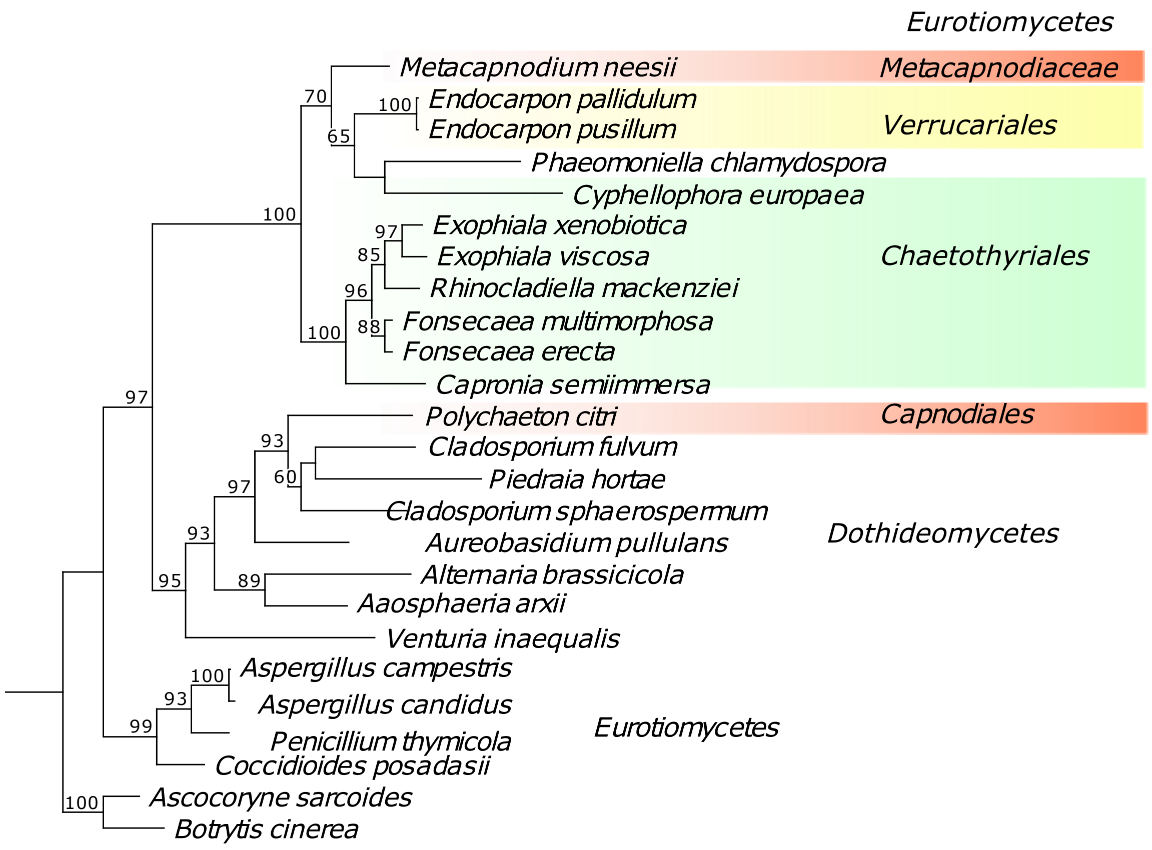

Supplement: Supplementary material 3 — Details of phylogenetic analyses [file mycokeys-129-163-s003.pdf]
